# Enzymatic Synthesis of Modified RNA Containing 5‑Methyl- or 5‑Ethylpyrimidines or Substituted 7‑Deazapurines and Influence of the Modifications on Stability, Translation, and CRISPR-Cas9 Cleavage

**DOI:** 10.1021/acschembio.5c00692

**Published:** 2025-11-06

**Authors:** Tania Sanchez-Quirante, Erika Kužmová, Miguel Riopedre-Fernandez, Sebastian Golojuch, Pavel Vopálenský, Veronika Raindlová, Afaf H. El-Sagheer, Tom Brown, Michal Hocek

**Affiliations:** † Institute of Organic Chemistry and Biochemistry, 89220 Czech Academy of Sciences, Flemingovo nam. 2, 16610 Prague 6, Czech Republic; ‡ Department of Organic Chemistry, Faculty of Science, Charles University in Prague, Hlavova 8, 12843 Prague 2, Czech Republic; § Department of Chemistry, Chemistry Research Laboratory, 6396 University of Oxford, Oxford OX1 3TA, United Kingdom; ∥ School of Chemistry and Chemical Engineering, University of Southampton, Highfield, Southampton SO17 1BJ, United Kingdom

## Abstract

A set of modified 5-methyl- and 5-ethylpyrimidine (uracil and cytosine) and 7-methyl-, 7-ethyl-, and 7-unsubstituted 7-deazapurine (deazaadenine and deazaguanine) ribonucleoside triphosphates was synthesized and used for enzymatic synthesis of base-modified RNA using *in vitro* transcription (IVT). They all were good substrates for T7 RNA polymerase in the IVT synthesis of model 70-mer RNA, mRNA encoding *Renilla* luciferase, and 99-mer single-guide RNA (sgRNA). The effect of modifications in the particular RNA on the stability and efficiency in *in vitro* and *in cellulo* translation as well as in CRISPR-Cas9 gene cleavage was quantified. In the *in vitro* translation assay, we observed moderately enhanced luciferase production with 5-methyluracil and -cytosine, while any 7-deazaadenines completely inhibited the translation. Surprisingly, *in cellulo* experiments showed a significant enhancement of translation with mRNA containing 7-deazaguanine and moderate enhancement with 5-methyl- or 5-ethylcytosine. Most of the modifications had a minimal effect on the efficiency of the gene cleavage in CRISPR-Cas9 except for 7-alkyl-7-deazaadenines that completely inhibited the cleavage. The results are important for further design of potential base-modified RNA therapeutics.

## Graphical abstract

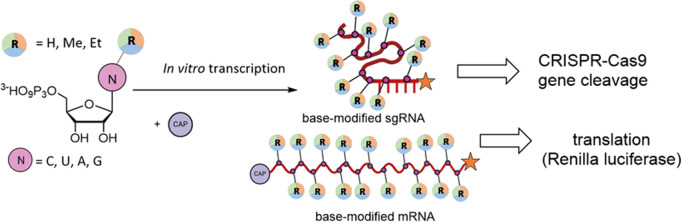

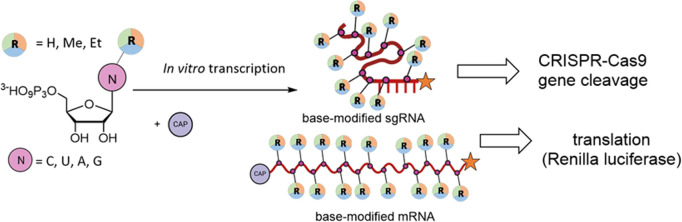

## Introduction

RNA therapeutics are currently revolutionizing^1^ modern medicine. The most prominent examples are mRNA vaccines that played a pivotal role in combating the SARS-CoV-2 pandemic.^2^ However, mRNA medicines in general show a groundbreaking potential in treatment of other diseases.^3–5^ Eukaryotic mRNAs contain a number of natural base modifications^6,7^ that modulate their stability^8^ and translational efficacy. Therefore, the presence of base-modified nucleotides is also crucial for mRNA therapeutics. The SARS-CoV-2 mRNA vaccines have N1-methylpseudouridine^9^ replacing all uridines in the sequence to decrease the immune response and increase the translation capacity through modulation of secondary structure formation. Apart from the obvious modification of 5′-caps of mRNA,^10^ there have been many studies of other base-modified nucleotides in mRNA vaccines and mRNAs in general,^11^ mostly focusing on the naturally occurring RNA bases, i.e., pseudouridine,^12,13^ 5-methylcytosine,^14–16^ 5-hydroxymethylcytosine,^16^ 5-methyluridine,^16,17^ *N* ^6^-methyladenosine,^18^ or 2,6-diaminopurine,^19^ some of which have shown increased translational efficacy.^15,16^ There is certainly a lot of space for studying other (natural or non-natural) modified nucleobases in mRNA as they can modulate stability, splicing, formation of secondary structures, recognition by proteins, etc.

CRISPR-Cas9 is a promising gene-editing tool for applications in biotechnology and in future medicines.^20^ It uses single-guide RNA (sgRNA), commonly ∼100 nt long RNA, that plays a critical role in target recognition and Cas9 complex formation. However, concerns over specificity and immunogenicity have driven efforts to chemically modify sgRNA.^21,22^ Most studies focused on increasing the stability of sgRNA through phosphate and/or sugar modifications,^23^ while much less attention has been paid to nucleobase modifications, mostly relying on testing some naturally occurring RNA nucleobases,^24^ i.e., *N*-methylpseudouridine,^25^ *N* ^6^-methyladenosine, or thiouridine.^26^ Interestingly, a conversion of 5-carboxycytidine to dihydrouridine was used to switch off the gene editing.^27^ Also, in this field, a systematic study of the influence of base modifications can shed some light on the interactions and lead to more efficient gene-editing systems.

*In vitro* transcription (IVT) with the T7 RNA polymerase (RNAP) is a common method of choice for enzymatic synthesis of RNA.^28,29^ However, the incorporation of base-modified nucleotides during IVT gives only uniformly modified transcript and is limited to ribonucleoside triphopshates (rNTPs), which are accepted by RNA polymerases.^30,31^ Previously, we reported that T7 RNAP has only a limited capacity for incorporation of nucleotides bearing bulkier substituents and base-modified guanosines.^32^ This limitation can be mitigated by the use of engineered DNA polymerases (e.g., TGK)^15,33–35^ that can accept modified rNTPs bearing bulkier modifications,^15^ reactive groups,^36^ or even small substituents at the position 2 of adenine^37^ and can be successfully used for the synthesis of even site-specifically or hypermodified RNA.

In this work, we systematically study how small alkyl groups (methyl and ethyl) at position 5 of pyrimidine and at position 7 of 7-deazapurine nucleobases affect rNTP substrate activity in IVT for the enzymatic synthesis of longer RNA, including mRNA and sgRNA. We also examine their impact on RNA stability and translation efficiency *in vitro* and *in cellulo* as well as on CRISPR-Cas9 gene editing. Although the translation and CRISPR-Cas9 DNA cleavage are completely different biological processes, they both involve interaction of RNA not only with the complementary nucleic acid (anticodon or target DNA) but also with initiation or elongation factors, ribosome, or Cas9 protein where modifications at the “major-groove edge” of nucleobases may play a significant role in their regulation. As mentioned above, 5-methylcytosine and 5-methyluracil are rare natural RNA nucleobases, while the ethyl derivatives are non-natural. Previously, we have shown an interesting stimulating effect of 5-ethyluracil in DNA on transcription by bacterial RNA polymerase^38^ and 5-ethyluridine was also incorporated into RNA by chemical synthesis and reported to be compatible with siRNA.^39^ For 7-deazapurines, we explored how the absence of *N* ^7^ nitrogen, important for hydrogen bonding and secondary structure formation, will influence the stability and performance of the RNA in translation and gene editing.

## Results and Discussion

### Synthesis of Modified Nucleoside Triphopshates

For our intended IVT construction of base-modified RNA, we designed and synthesized a small portfolio of modified nucleoside triphosphates (NTPs) derived from 5-substituted pyrimidines and 7-substituted 7-deazapurines. In the pyrimidine series (uridine and cytidine), we synthesized 5-methyl and 5-ethyl derivatives, while in the 7-deazaadenosine and 7-deazaguanosine series, we synthesized and studied not only the corresponding 7-methyl and 7-ethyl but also the 7-unsubstituted derivatives. The modified NTPs were synthesized from the corresponding nucleosides by triphosphorylation (Figure 1). The methyl NTPs (**C** ^**Me**^ **TP** (**5**),^40^ **A** ^**Me**^ **TP** (**7**),^32^ and **G** ^**Me**^ **TP** (**8**)^32^) were prepared from the corresponding methyl-substituted nucleoside (**1**,**3**,**4**) according to previously reported procedures using the sequence of reaction with POCl_3_, followed by (NHBu_3_)_2_H_2_P_2_O_7_ and triethylammonium bicarbonate (TEAB). **U** ^**Me**^ **TP** (**6**) was prepared analogously from known 5-methyluridine (**2**). The synthesis of ethyl-substituted nucleosides (**N** ^**Et**^, **13**–**16**) was performed through catalytic hydrogenation of previously reported^32^ 5-ethynylpyrimidine or 7-ethynyl-7-deazapurine nucleosides (**N** ^**E**^, **9**–**12**). The corresponding **N** ^**Et**^ **TPs** (**17**–**20**) were prepared by an analogous triphosphorylation in 6–30% yields. The unsubstituted deazapurine nucleoside triphosphates (**A** ^**H**^ **TP** (**23**) and **G** ^**H**^ **TP** (**24**)) were prepared by the triphosphorylation of 7-deazaadenosine (**21**) or 7-deazaguanosine (**22**) obtained by catalytic hydrogenation of the corresponding 7-iodo derivatives.^41^ All new nucleosides and nucleotides (Section 1.7 in the SI) were characterized by NMR spectroscopy and mass spectrometry (Figures S24–S29 in the SI).

**Figure 1:**
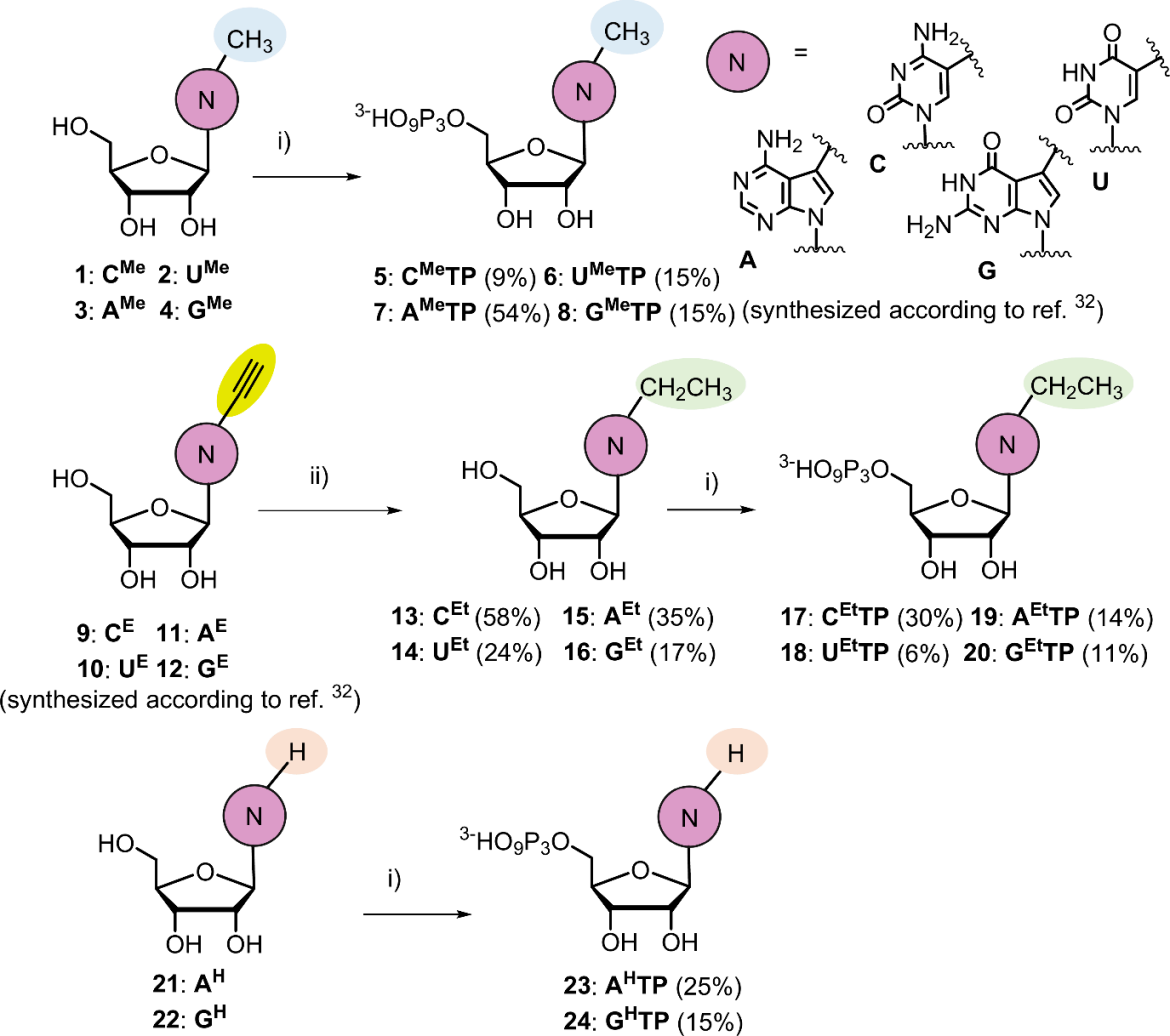
Synthesis of modified nucleosides and modified nucleoside triphosphates. Reagents and conditions: (i) 1: POCl_3_, PO­(OMe)_3_, 0 °C; 2: (NHBu_3_)_2_H_2_P_2_O_7_, Bu_3_N, DMF, 0 °C; 3: 2 M TEAB. (ii) H_2_ (1 atm.), 10% Pd/C, 5–12 h, 22 °C. For details, see Sections 1.1–1.7 in the SI.

### Enzymatic RNA Synthesis

In this study, three different types of modified RNAs of varying lengths were synthesized via *in vitro* transcription (IVT). Details of all sequences used in this study are listed in Table S1 and Table S2 in the SI: 71 nt long RNA (**cap71RNA_N** ^**R**^) to test capping efficiency, the second 99 nt long sgRNA to evaluate its stability and CRISPR-Cas cleavage efficiency (**sgRNA_N** ^**R**^), and the third mRNA encoding for *Renilla* Luciferase to study its translation efficiency *in vitro* and *in cellulo* using HeLa S3 cells (**mRNA_N** ^**R**^) (Figure 2A). All IVTs were performed using a HiScribe T7 high Yield RNA synthesis Kit in a final volume of 10 μL at 37 °C for 3 or 4 h. After transcription, the DNA template was removed by treatment with DNase I. EDTA was then added to inactivate the T7 RNA polymerase by chelating Mg^2+^ ions. All samples were purified using Monarch RNA Cleanup Kit columns (details in Section 2.3.3 in the SI). To enable visualization and analysis by denaturing PAGE, the RNA samples were post-transcriptionally labeled using T4 RNA ligase and pCp-ATTO-488, which attached the ATTO-488 labeled cytidine at the 3′-end (General Procedure (IV), Section 2.3.2 in the SI).^42^ Standard SYBR Gold staining was not practical for the visualization of some of the base-modifed RNAs because modified 7-deazaguanines quench fluorescence of intercalating dyes.^43^ In all cases, we used one of the modified **N** ^**R**^ **TP** instead of the corresponding natural NTP. Since we suspected that modified deazapurine nucleobases could potentially influence the absorbance-based concentration measurements for RNA containing these nucleobases, we performed primer extension (PEX)^15^ to synthesize **98RNA_nat**, **98RNA_A** ^**H**^, and **98RNA_G** ^**H**^ using engineered TGK DNA polymerase and primer containing a Cy5 fluorescent label at the 5′-end. Fluorescence intensity of samples with different concentrations were measured to confirm the accuracy of the concentration determination by UV absorbance and demonstrate that deazapurines do not significantly change the absorbance as compared to their natural counterparts (Figures S20 and S21, Table S12, and Section 2.9 in the SI).

**Figure 2:**
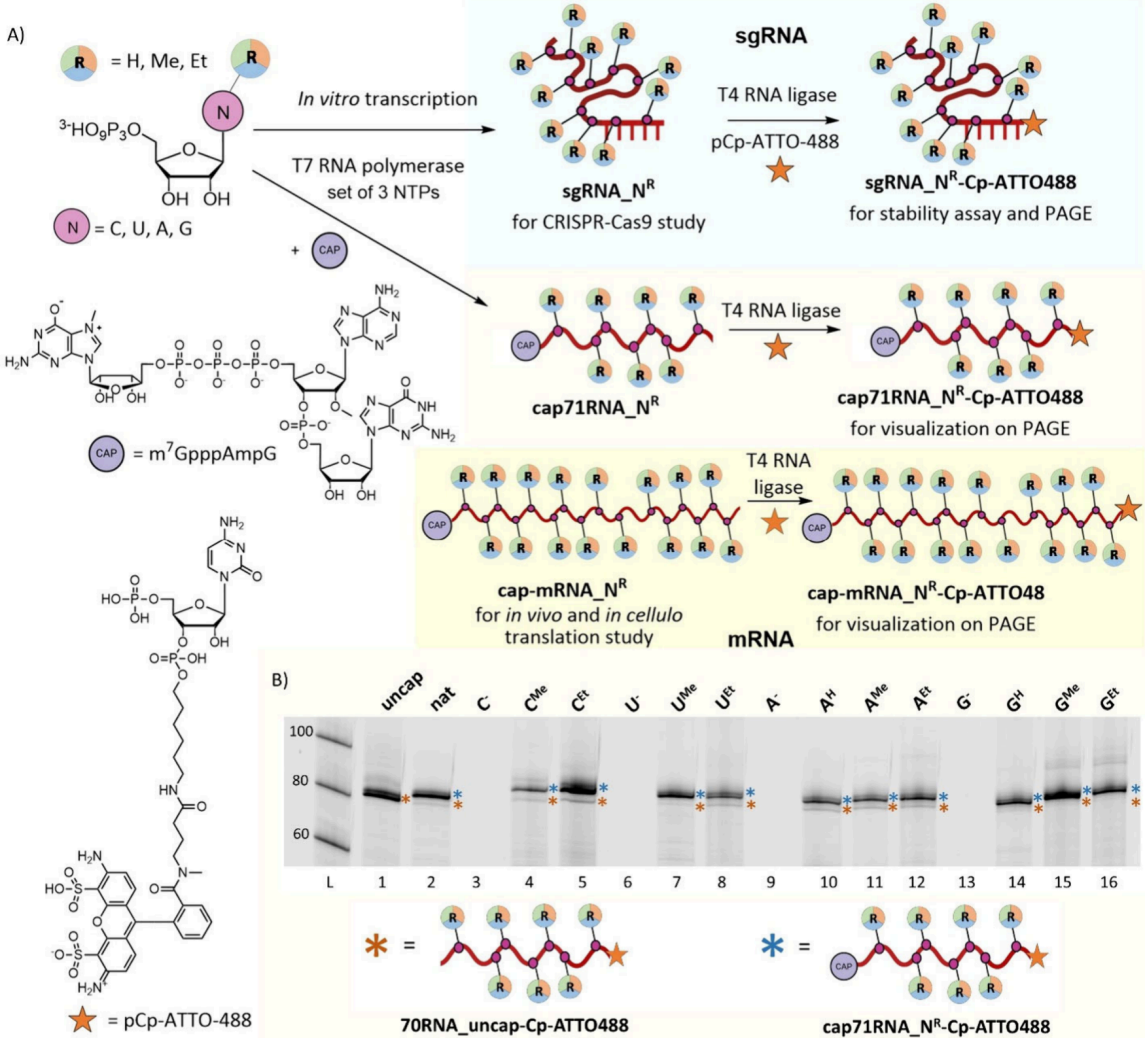
(A) General scheme for the synthesis of modified RNAs (**cap71RNA_N** ^**R**^, **sgRNA_N** ^**R**^, **cap-mRNA_N** ^**R**^, **cap71RNA_N** ^**R**^ **-Cp-ATTO488**, **sgRNA_N** ^**R**^ **-Cp-ATTO488**, and **cap-mRNA_N** ^**R**^ **-Cp-ATTO488**; **R** = **H**, **Me**, **Et**) via in vitro transcription (IVT). Reactions were performed using T7 RNA polymerase and DNA templates encoding the respective sequences. For the synthesis of natural RNA, the four natural NTPs were used, while for the incorporation of one modification, three natural NTPs and the corresponding modified **N^R^TP** were used. 3′ end labeling of RNA using T4 RNA ligase 1 and pCp-ATTO-488 enables site-specific attachment of a fluorescent tag for RNA visualization. m^7^GpppAmpG was used as a cap. (B) 20% dPAGE analysis of the transcription reaction of **cap71RNA_N** ^**R**^ **-Cp-ATTO488**. (L) DNA ladder, (1) only natural NTPs, (2) natural NTPs and cap, (3, 6, 9, 13) negative control, three natural NTPs and cap, without the modified **N** ^**R**^ **TP** of interest, (4, 5, 7, 8, 10, 11, 12, 14, 15, 16) three natural NTPs and cap, with the modified **N** ^**R**^ **TP** of interest. The uncropped gel is shown in Figure S3 in the SI.

### Capped 71-mer RNA

Due to the important role of RNA caps, there is a strong interest in developing chemically synthesized cap analogues that can enhance mRNA stability by resisting hydrolysis, inhibit or activate cell processes, or interact more specifically with some proteins.^44^ In this study, we tested the previously reported^45^ 5′cap m^7^GpppAmpG (Figure 2) expected to give a high capping co-transcriptional efficiency in IVT synthesis of capped RNA. We tested it on short-modified RNA (**cap71RNA_N** ^**R**^) that were designed as a model for long mRNA but would better reflect the changes in electrophoretic mobility between capped and uncapped RNA products. The DNA template contained 2′-*O*-Me modifications at the last two nucleotides of the antisense oligonucleotide to prevent nontemplate incorporation by T7 RNAP.^46^ The results of the IVT experiments in the presence of the m^7^GpppAmpG are shown in Figure 2B. The positive control experiments with all four natural NTPs in the absence and in the presence of the cap (Figure 2B, lanes 1,2) show a distinct difference in mobility of the uncapped and capped RNA. The IVT experiments with modified **N** ^**R**^ **TPs** in the presence of the cap (lanes 4, 5, 7, 8, 10–12, and 14–16) demonstrate that all the modified nucleotides were good substrates for the T7 RNA polymerase and, in all cases, the capped RNA was the main product accompanied by only trace amounts of the uncapped RNA, indicating capping efficiencies above 90% (Table S3 in the SI). Moreover, all modified capped RNAs were characterized by LC-MS (Table S4 in the SI).

### mRNA Encoding *Renilla* Luciferase

Having proof of the good substrate activity and cotranslational capping efficiency, we used the same procedure for the IVT synthesis of modified mRNA (**cap-mRNA_N** ^**R**^). We designed a set of modified mRNAs encoding *Renilla* luciferase (**cap-mRNA_N** ^**R**^). The DNA template was prepared by the linearization of the corresponding plasmid (Figure S6 in the SI). Post-transcriptionally labeled natural and modified mRNA were analyzed by 7.5% dPAGE (Figure S7 in the SI) using either a posttranscriptional labeling (Figure S7A in the SI) or SybrGold staining (Figure S7B in the SI). The SybrGold did not stain the 7-deazaguanosine containing RNA due to quenching of fluorescence^43^ but has shown good transcription yields with modified **A** ^**R**^ **TP**, **C** ^**R**^ **TP**, and **U** ^**R**^ **TP** nucleotides.

The modified mRNAs were tested in the Rabbit Reticulocyte Lysate (RRL) System to assess their *in vitro* translation efficiency (Figure 3A).^47^ To further validate the translation efficiency, we also compared protein synthesis levels using 10% SDS PAGE analysis (Figure S14 and Section 2.7.1 in the SI). Interestingly, the 5-methyluridine-containing **cap-mRNA_U** ^**Me**^ and 5-methylcytidine-containing **cap-mRNA_C** ^**Me**^ demonstrated significantly enhanced translation efficiency as shown both by luminescence (Figure 3B) and 10% SDS PAGE (Figure S14 in the SI). Homologous **cap-mRNA_C** ^**Et**^ showed similar efficiency to natural mRNA, while **cap-mRNA_U** ^**Et**^ and **cap-mRNA_G** ^**H**^ gave lower but still significant protein formation. On the other hand, the other modified mRNAs, **cap-mRNA_A** ^**H**^, **cap-mRNA_A** ^**Me**^, **cap-mRNA_A** ^**Et**^, **cap-mRNA_G** ^**Me**^, and **cap-mRNA_G** ^**Et**^, afforded very low or negligible translation. These results are partly in accord with Karikó and Weissman^12^ who reported good efficiency for m^5^C and m^5^U mRNAs, but in their case, it was comparable to natural mRNA.

**Figure 3:**
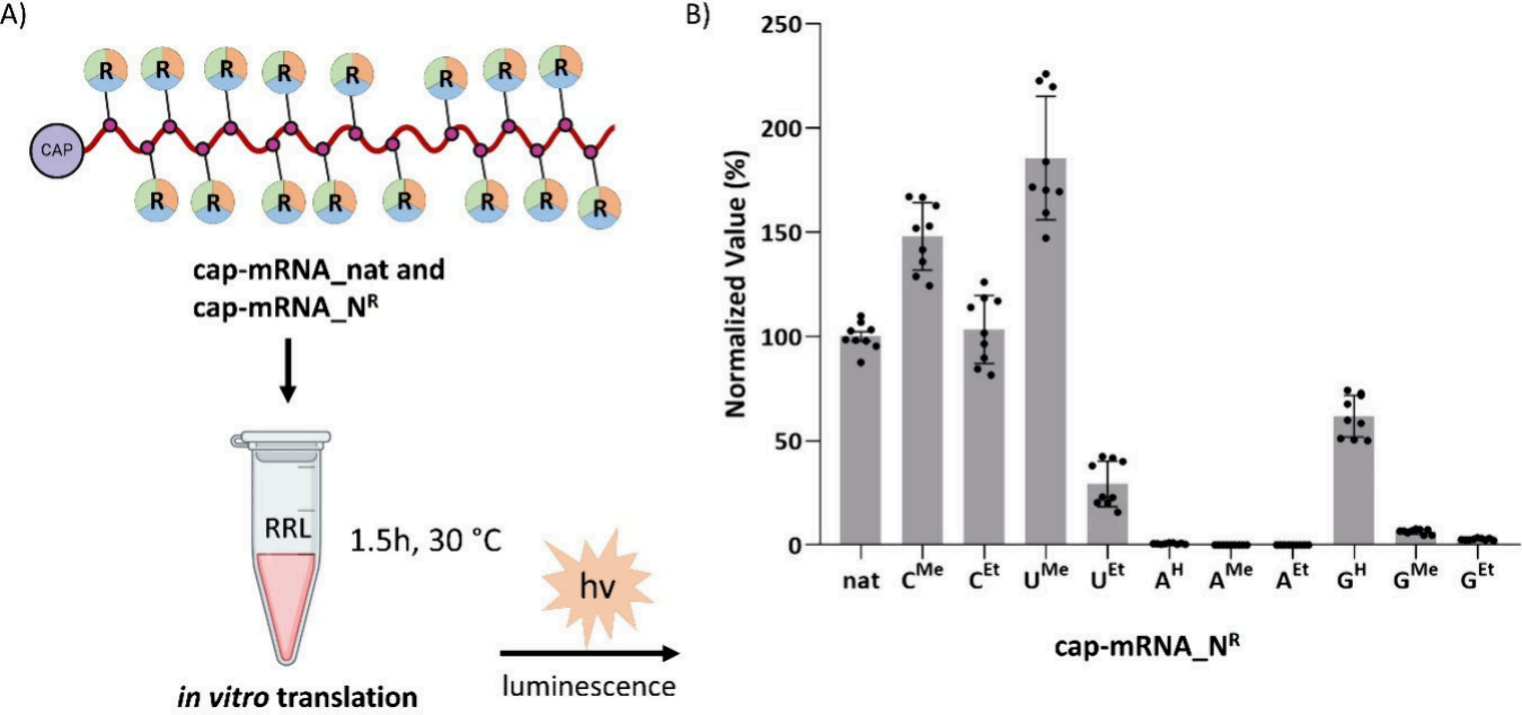
(A) *In vitro* translation analysis of natural mRNA (**cap-mRNA_nat**) and modified mRNA (**cap-mRNA_N** ^**R**^) encoding *Renilla* Luciferase using Rabbit Reticulocyte Lysate (RRL). (B) Translation efficiency is expressed as the luminescence signal normalized to natural mRNA (**cap-mRNA_nat**) set as 100% (Figure S13, Section 2.7 in the SI). Data represent mean ± SD from three biological replicates (*n* = 3), each performed in technical triplicate (Table S8, Section 2.7 in the SI). GraphPad Prism was used for data analysis and visualization.

To test the *in cellulo* translation, the same set of modified mRNAs (**cap-mRNA_N** ^**R**^) were transfected using Lipofectamine MessengerMax in Hela S3 cells (Figure 4A). At defined time points (3, 6, 12, 24, and 36 h post-transfection), the cells were collected for downstream procedures (Section 2.8 in the SI), i.e., study of transfection and translation efficacy through luciferase activity measurements. To ensure that the transfection of modified mRNAs did not alter the growth of the cells, cell proliferation was monitored on IncuCyte (Figure S18, Section 2.8.4 in the SI). To verify the transfection efficiency and stability of modified mRNA, for selected **cap-mRNA_N** ^**R**^ which contained examples that gave significant translation as well as examples of translationally inactive 7-deazaadenine-containing mRNA, the total RNA was isolated from transfected cells and reverse-transcribed into **cDNA_N** ^**R**^. For selected mRNAs, the absolute amount of transfected modified mRNAs at 3 and 6 h was reverse transcribed to **cDNA_N** ^**R**^ and was analyzed by digital droplet PCR (Figure 4B, Section 2.8.2 in the SI). Most of the modified **cap-mRNA_N** ^**R**^ containing 5-methylpyrimidine or unsubstituted 7-deazapurine modifications showed levels similar to those of the nonmodified mRNA confirming that the transfection and stability are comparable to those of the natural control in the cell. On the other hand, mRNAs containing 7-alkyl-7-deazapurines showed somewhat lower quantities of the corresponding cDNA, indicating that either the transfection was less efficient or the mRNA was less stable.

**Figure 4:**
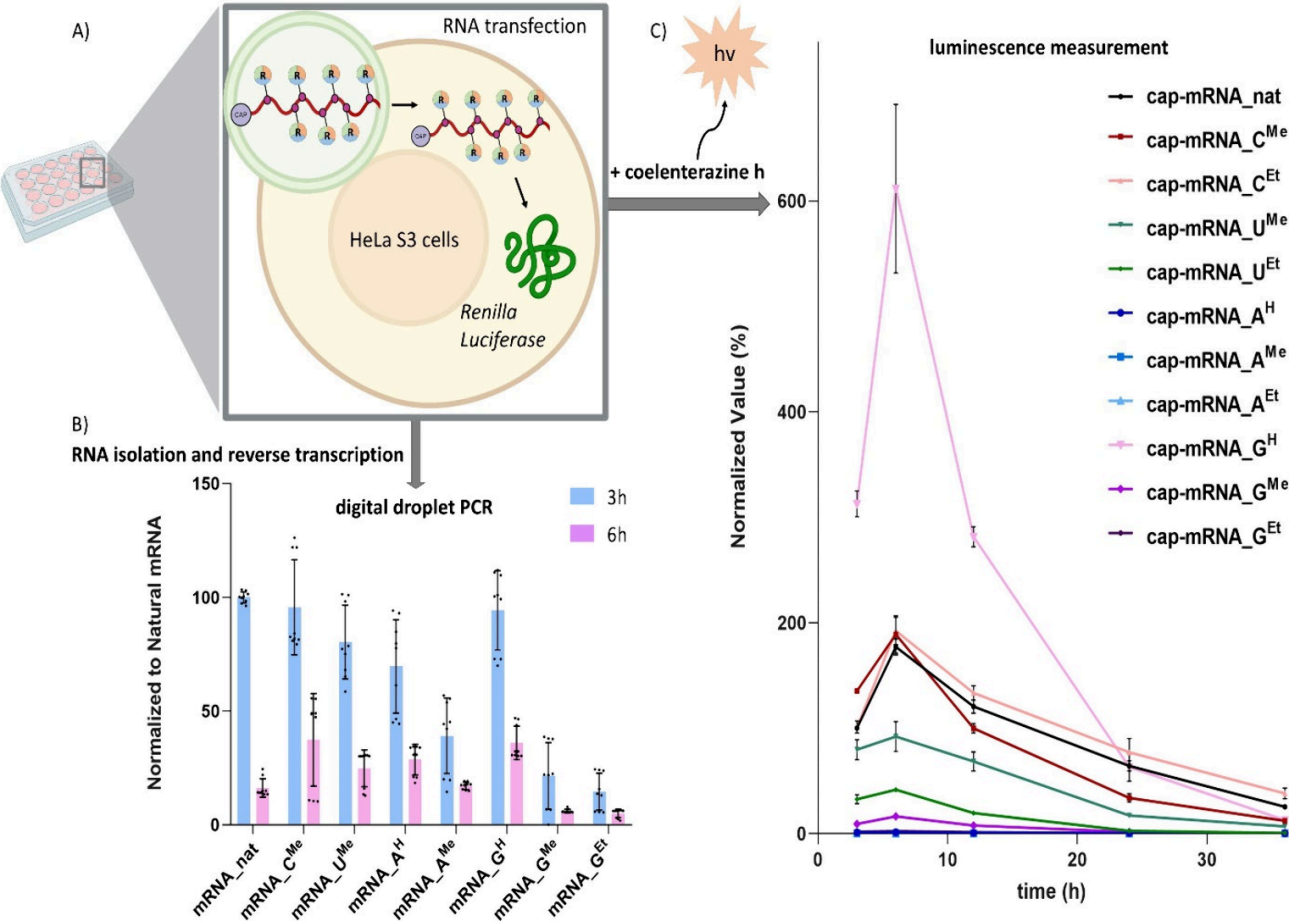
(A) Schematic of the experimental workflow. HeLa S3 cells were transfected with **cap-mRNA_nat** and modified mRNA **cap-mRNA_N** ^**R**^ using Lipofectamine MessengerMAX and collected at 3, 6, 12, 24, and 36 h post-transfection. (B) Intracellular levels of selected modified mRNAs. Total RNA (3 and 6 h) was reverse-transcribed to **cDNA_N** ^**R**^ and quantified by ddPCR. As cDNA levels reflect original mRNA abundance, absolute transcript copies/ng RNA are used to represent the levels of the transfected mRNA. Values are normalized to **cDNA_nat** at 3 h (set as 100%) and represent mean ± SD from three biological replicates (*n* = 3), each performed in technical triplicate. (C) Time-course *Renilla* luciferase activity from cells transfected with the indicated **cap-mRNA_N** ^**R**^. Data are normalized to the luminescence signal from **cap-mRNA_nat** at 3 h (set as 100%) and represent mean ± SD from three biological replicates (*n* = 3), each performed in technical triplicate. GraphPad Prism was used for data analysis and visualization (Figures S15 and S16, Table S9 and Table S10, Section 2.8 in the SI).

To directly measure translation efficiency, luciferase activity was quantified at several defined time points.^48^ The results are summarized in Figure 4C (and in Figure S15 and Section 2.8.1 in the SI). Surprisingly, **cap-mRNA_G** ^**H**^ showed the highest translational efficiency with very fast onset, producing luminescence approximately 3-fold higher than the natural mRNA (**cap-mRNA_nat**) at the earliest time points (3 and 6 h) post-transfection but with significant decrease after 24 h. **Cap-mRNA_C** ^**Me**^ and **cap-mRNA_C** ^**Et**^ were translated comparably to the natural mRNA (control), while **cap-mRNA_U** ^**Me**^, **cap-mRNA_U** ^**Et**^, and **cap-mRNA_G** ^**Me**^ produced lower, but still measurable, levels of luminescence. All 7-deazaadenine-containing mRNA (**cap-mRNA_A** ^**H**^, **cap-mRNA_A** ^**Me**^, and **cap-mRNA_A** ^**Et**^) as well as **cap-mRNA_G** ^**Et**^ produced no measurable signal, consistent with their poor *in vitro* translation performance (see above).

To validate the observed translation efficiencies, dual-luciferase assays were performed to transfect **cap-mRNA_N** ^**R**^ and a capped *Firefly* luciferase control (**mRNA_natFF**) (Figure[Fig fig5]). Although the level of luminescence was generally lower compared to single-reporter data, the results corroborated the effect of 7-deazaguanine (**cap-mRNA_G** ^**H**^) on the fast onset of translation showing about twice the higher level of translation after 6 h but then a relatively fast decrease after 24 h. It is important to note a potential limitation of dual-reporter assays where the co-transfection of two mRNAs may lead to competition for shared translational resources (ribosomes, initiation factors, etc.); when under high transcript load, increased expression of one mRNA can negatively impact the translation of another.^49–51^ This provides a mechanistic rationale for the relatively lower translation efficiency in the dual-luciferase assay. Therefore, while the dual-luciferase experiments confirmed the enhanced translation of **cap-mRNA_G** ^**H**^, we emphasize that the single-reporter luciferase data, free from such competition artifacts, provide a more definitive validation of its superior *in cellulo* translation performance. To ensure that the co-transfection did not alter the growth of the cells, cell proliferation was also monitored on IncuCyte (Figure S19, Section 2.8.4 in the SI).

**Figure 5:**
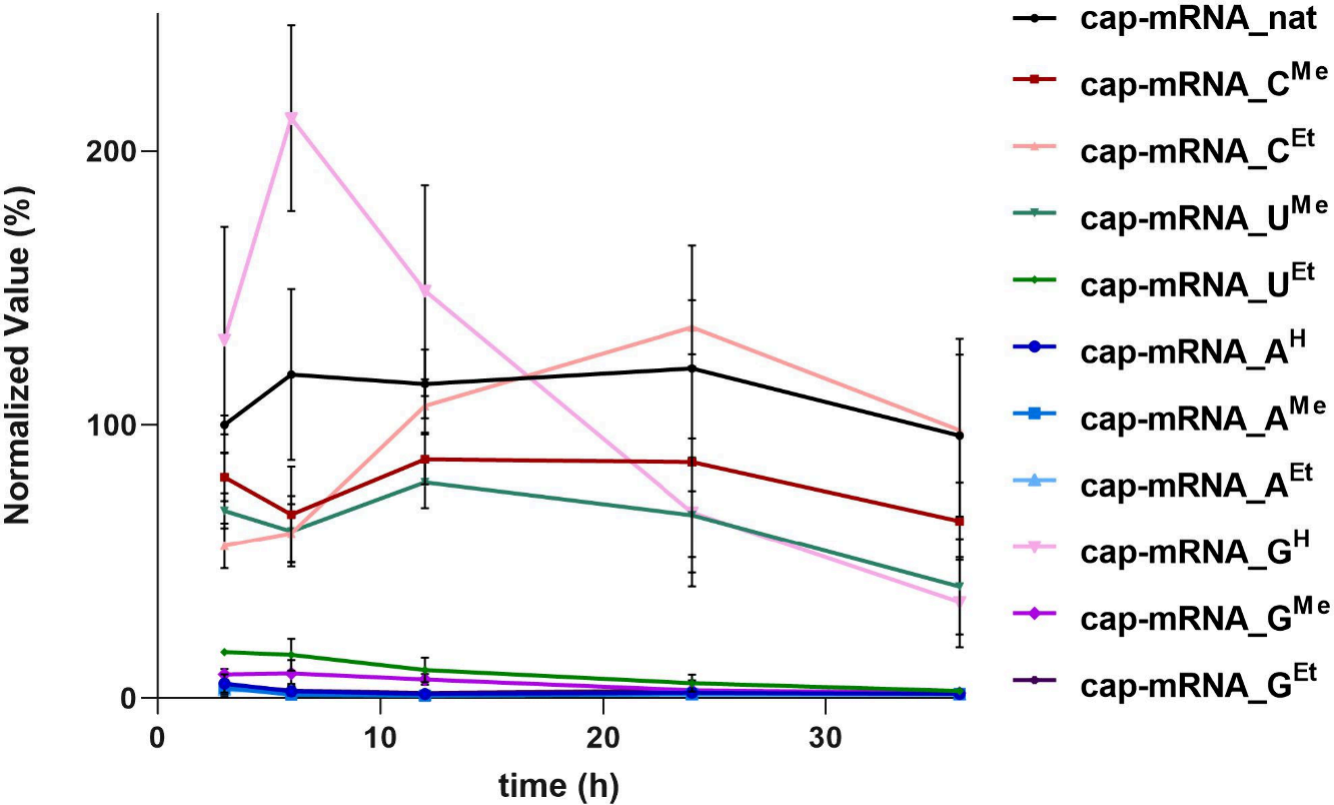
Time-course analysis of *Renilla* luciferase expression in HeLa S3 cells cotransfected with natural (**cap-mRNA_nat**) or modified (**cap-mRNA_N** ^**R**^) mRNA encoding *Firefly* luciferase (**mRNA_natFF**) using Lipofectamine MessengerMAX and collected at 3, 6, 12, 24, and 36 h post-transfection. Data are normalized to the luminescence signal of **mRNA_natFF** and **cap-mRNA_nat** at 3 h (set as 100%) and represent mean ± SD from three biological replicates (*n* = 3), each performed in technical triplicate.

The high *in cellulo* translation of 7-deazaguanine **cap-mRNA_G** ^**H**^ (both in single and dual reporter assay) is particularly noteworthy, because its intracellular level measured by ddPCR was similar to the natural one and in the *in vitro* assay this mRNA did not show enhanced translation. This discrepancy suggests that the fast onset and enhanced *in cellulo* translation may be due to the highly efficient ribosome recruitment and translation initiation shortly after transfection. A possible reason for the enhanced translational efficiency could be in its altered ability to form secondary structures, as the 7-deazaguanines cannot form G quadruplexes or Hoogsteen base pairs. Mechanistic understanding of the effect will certainly require further studies.

Another interesting dichotomy is the translational performance of **cap-mRNA_C** ^**Me**^, **cap-mRNA_C** ^**Et**^, and **cap-mRNA_U** ^**Me**^, which showed the highest *in vitro* translation efficiency, but *in cellulo*, they were comparable or somewhat worse (but not better) than the natural mRNA. These findings are in accordance with previous work reporting that the enhancement of *in vivo* translation efficacy of mRNA containing **U** ^**Me**^ or **C** ^**Me**^ was significant only in longer-term protein expression (over 10 days).^52^ The other modified mRNAs were less effective, which could be due to structural changes affecting ribosome scanning, reduced interactions with translation initiation factors, or altered mRNA stability. All these effects are even more pronounced *in cellulo* in the complex intracellular environment. The other modified mRNAs, **cap-mRNA_U** ^**Et**^ and **cap-mRNA_G** ^**Me**^, gave very reduced but detectable luminescence, while **cap-mRNA_A** ^**H**^, **cap-mRNA_A** ^**Me**^, **cap-mRNA_A** ^**Et**^, and **cap-mRNA_G** ^**Et**^ were not translated at all in our cell system.

### sgRNA and CRISPR-Cas9

For the study of the influence of base-modification on cleavage of target DNA by CRISPR-Cas9, we designed a set of base-modified 99-nt single-guide RNA (sgRNA) and synthesized them by IVT. In this case, both strands of the DNA template were chemically synthesized using a DNA synthesizer. After purification, the strands were annealed to form a double-stranded DNA template (for sequences, see Table S1 in Section 2.3.4.1 in the SI) and amplified by PCR (Figure S4 in the SI), which was then ready for IVT (Section 2.3.4.2 in the SI). Both natural and modified sgRNA were characterized by LC-MS (Table S5 in the SI) and subsequently post-transcriptionally labeled by Cp-ATTO-488 for visualization and analysis by 15% dPAGE (Figure S5 in the SI).

Stability experiments in human serum were performed to evaluate the resistance of both labeled natural (**sgRNA_nat-Cp-ATTO-488**) and chemically modified (**sgRNA_N** ^**R**^ **-Cp-ATTO-488**) sgRNAs (General Procedure (IV) in Section 2.3.2 in the SI). Each sgRNA was mixed with Cas9 nuclease and incubated at 37 °C for 10 min to allow complex formation. Subsequently, human serum and PBS were added, and the mixtures were further incubated at 37 °C. Aliquots were collected at 0, 15, and 30 min, as well as at 1 and 2 h. After treatment with Proteinase K to degrade Cas9 protein, the samples were analyzed by 15% denaturing PAGE (Figure S9 in the SI). Remaining sgRNA was quantified over time (Figure S10 in the SI), and the half-lives were calculated by fitting one phase decay model in GraphPad (Figure S11 in the SI). Each analysis was done in triplicate, and the results are summarized in Figure 6B, indicating that smaller modifications (7-deazapurines or methyl derivatives) increased the stability of sgRNA, whereas bulkier ethyl groups caused a minor decrease of stability. Accordingly, **sgRNA_A** ^**H**^ and **sgRNA_G** ^**H**^ were the most resistant, along with **sgRNA_C** ^**Me**^ and **sgRNA_A** ^**Me**^, while uracil modifications were not stabilizing.

**Figure 6:**
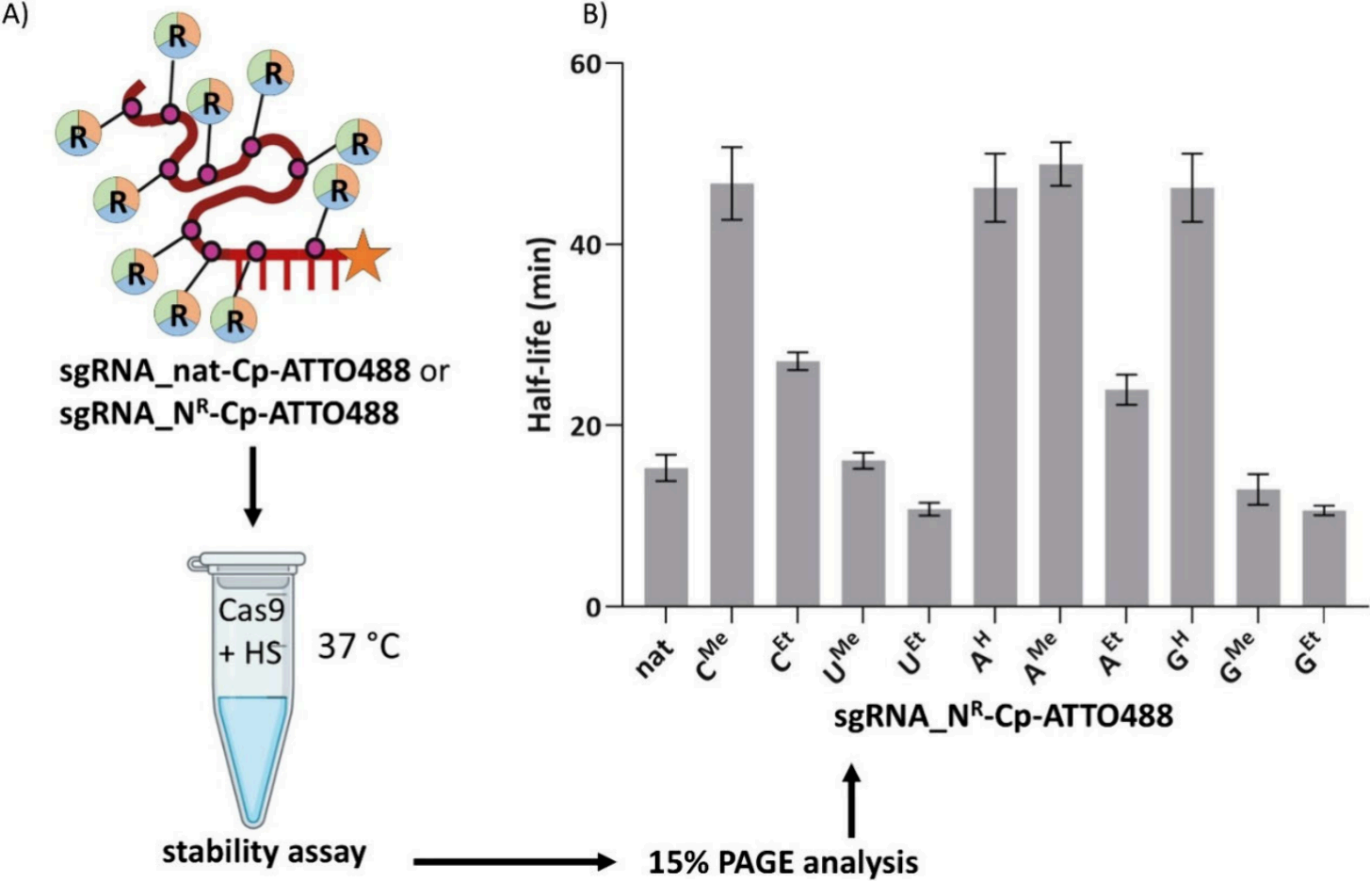
(A) Schematic representation of stability experiment of labeled **sgRNA_nat-Cp-ATTO-488** and **sgRNA_N** ^**R**^ **-Cp-ATTO-488** in human serum (HS). (B) Normalized degradation data from three independent experiments were averaged and fitted with a one-phase exponential decay model in GraphPad Prism. The reported half-life was calculated from the average degradation curve. Curve fitting yielded *R* ^2^ values ranging from 0.8863 to 0.9891 (mean *R* ^2^ = 0.957), indicating a good fit to the decay model. Data are shown as mean ± SEM (*n* = 3) (Table S7, Section 2.6.1 in the SI).

Using the full set of unlabeled **sgRNA_N** ^**R**^, CRISPR-Cas mediated cleavage experiments were carried out targeting the adeno-associated virus integration site 1 (AAVS1) genomic locus. Cleavage efficiency was analyzed by electrophoresis on chip (Figure 7A and Figure S8 in the SI). Our results (Figure 7B) demonstrate that most nucleotide modifications introduced into the sgRNA have minimal effects on cleavage efficiency and do not disrupt Cas9 activity in the AAVS1 target site in comparison to **sgRNA_nat**. However, when 7-methyl-7-deazaadenosine and 7-ethyl-7-deazaadenosine were incorporated into the RNA (**sgRNA_A** ^**Me**^ and **sgRNA_A** ^**Et**^, respectively), it resulted in a complete loss of cleavage activity. This loss may happen for two different reasons: either Cas9 is not able to recognize and bind **sgRNA_A** ^**Me**^ or Cas9 catalytic activity is compromised.

**Figure 7:**
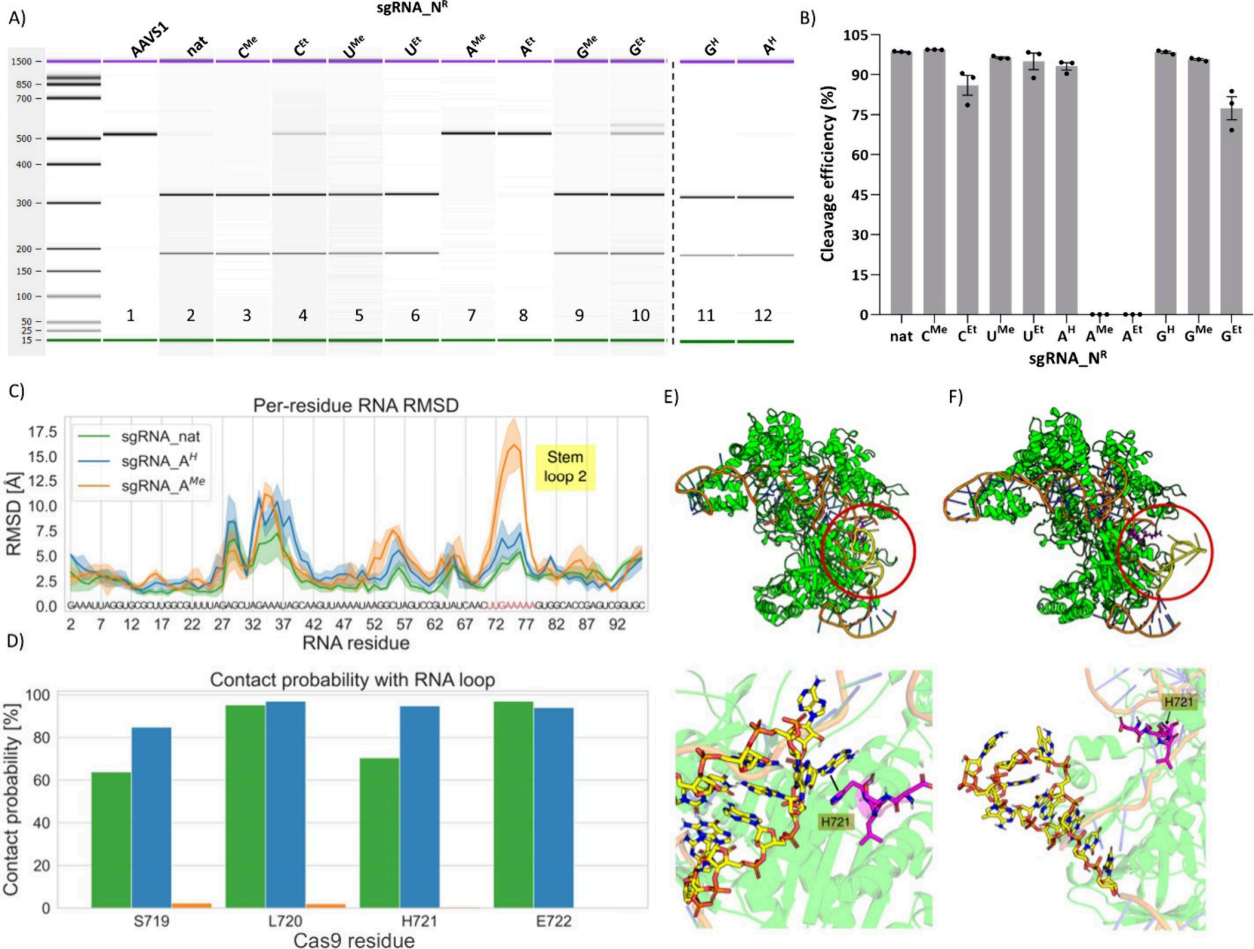
(A) Electrophoresis on a chip analysis of CRISPR-Cas9 cleavage of the AAVS1 target using natural and chemically modified **sgRNA_N** ^**R**^: (1) uncleaved full-length AAVS1 DNA; (2) cleavage with natural sgRNA (**sgRNA_nat**); and (3–12) cleavage with various modified sgRNAs (**sgRNA_N** ^**R**^) (Figure S8A, Section 2.5 in the SI). (B) Quantification of AAVS1 cleavage efficiency (%) by Cas9 in the presence of natural (**sgRNA_nat**) or modified (**sgRNA_N** ^**R**^) sgRNAs, based on fragment analysis. Data represent mean ± SEM from three independent experiments (*n* = 3) (Figure S8B, Table S6, and Section 2.5 in the SI). (C) Per residue root-mean-square deviation (RMSD) of the RNA backbone for **sgRNA_nat** (green), **sgRNA_A** ^**H**^ (blue), and **sgRNA_A** ^**Me**^ (orange). The standard deviation from replicates is shown as shaded areas. (D) Average contact probability of RNA Stem Loop 2 with selected residues from the Cas9 protein. Simulation frames where at least one atom from one of the RNA residues on the Stem Loop (71–78) and one atom from the four affected amino acids (719–722) where closer than 5 Å was considered a contact. (E) Top: representative structure from the molecular dynamics of the Cas9 protein (green), bound to unmodified sgRNA (**sgRNA_nat**). Inside of the red circle, the RNA Stem Loop 2 region, employed in previous analysis, is highlighted in yellow. The four selected residues from the protein are shown in magenta. Bottom: zoomed picture of the region inside the circle, highlighting the interaction between Stem Loop 2 and Cas9. The π-stacking interaction between the RNA and the histidine 721 (H721) is highlighted. (F) Equivalent representation from **sgRNA_A** ^**Me**^ in complex with Cas9. Notice the separation of Stem Loop 2 from the protein in this case.

To assess the first hypothesis, we performed a stability experiment in human serum using **sgRNA_A** ^**H**^ and **sgRNA_A** ^**Me**^ in the absence of Cas9, which normally protects sgRNAs from rapid degradation (Figure S12 in the SI). Both **sgRNA_A** ^**H**^ and **sgRNA_A** ^**Me**^ were rapidly degraded with detectable RNA present only at the 0 min time point. These findings suggest that Cas9 successfully binds both sgRNAs and that the observed loss of cleavage is not due to disrupted sgRNA recognition.

To understand the mechanistic basis of this loss of activity, we performed molecular dynamics simulations (MD) to examine how these modifications might perturb critical structural interactions between sgRNA and Cas9, starting from the published Cas9:sgRNA:DNA structure (PDB: 4OO8).^53,54^ Simulations were performed for **sgRNA_nat** as well as the **sgRNA_A** ^**H**^ and **sgRNA_A** ^**Me**^ variants (Figure 7, Figure S22, Section 2.10 in the SI). Notably, our simulations identified a pronounced conformational distortion between nucleobases U71 and A78, the region that corresponds to Stem Loop 2 (Figure S23 in the SI), reflected in a spike in the root-mean-square deviation (RMSD) of the RNA backbone (Figure 7C). This region contributes to sgRNA structural stability and enhances the DNA cleavage efficiency. Stem Loop 2 is rich in adenosines, making it particularly susceptible to our observed modification-induced conformational changes.^53^ Previous studies have demonstrated that altering its length disrupts Cas9 activity,^54^ emphasizing its functional importance.

These RNA structural changes observed with **sgRNA_A** ^**Me**^ involve the loss of several key contacts with the protein compared to **sgRNA_nat** and **sgRNA_A** ^**H**^, leading to the detachment of Stem Loop 2 from Cas9 (Figure 7D,F) compared to **sgRNA_nat** (Figure 7E). Among these disrupted sgRNA-protein interactions, the loss of π-stacking between adenosine and H721 (Histidine 721) is especially significant in the case of **sgRNA_A** ^**Me**^ (Figure 7F). Additional affected residues include S719, L720, and E722, all located at the N-terminus of the RuvC-II domain, a region critical for nontarget strand cleavage.^55^

To sum up, our simulations offer a mechanistic explanation for the functional inactivation observed with **sgRNA_A** ^**Me**^, while other methylated modifications (e.g., **sgRNA_G** ^**Me**^, **sgRNA_U** ^**Me**^, or **sgRNA_C** ^**Me**^) should not cause it. The incorporation of 7-methyl-7-deazaadenosine (**sgRNA_A** ^**Me**^) destabilizes an adenine rich RNA loop, while other modifications would not affect the same region. While **sgRNA_A** ^**H**^ preserves overall base pairing and RNA folding, adding a methyl group at position 7 creates steric hindrance, affecting the RNA-protein interaction and the nuclease effect of Cas9.

## Conclusions

In this study, we report the synthesis of a complete set of modified ribonucleside triphosphates derived from 5-substituted pyrimidines and 7-substituted 7-deazapurines bearing methyl or ethyl groups (as well as unsubstituted 7-deazapurines) and their use as substrates for T7RNA polymerase in the enzymatic synthesis of different types of RNAs by IVT. All of these modified **N** ^**R**^ **TP**s were good substrates, and we were able to synthesize model 70mer **70RNA_N** ^**R**^ sequences, their capped versions **cap71RNA_N** ^**R**^, and capped **cap-mRNA_N** ^**R**^ encoding for *Renilla* luciferase, as well as 99-mer single-guide RNAs **sgRNA_N** ^**R**^. The modifications did not affect the capping efficiency when using Cap1 in the IVT. In all cases, we also synthesized 3′-Cp-ATTO488 labeled versions for visualization on PAGE using T4 RNA ligase and pCp-ATTO-488 and all 70–99-mer modified RNAs were also characterized by LC-MS.

The **cap-mRNA_N** ^**R**^ encoding for *Renilla* luciferase was used for assessing the translation efficiency by luminescence in the Rabbit Reticulocyte System *in vitro* and in Hela S3 cells *in cellulo*. Interestingly, 5-methyluracil-conntaining **cap-mRNA_U** ^**Me**^ exhibited the highest translation efficiency in the cell-free system, while 7-deazaguanine-containing **cap-mRNA_G** ^**H**^ demonstrated remarkably fast and significantly enhanced translation efficiency with an early onset post-transfection in the cellular system. Any 7-deazaadenine-containing modified mRNAs were not translated in any of the systems, indicating that the *N* ^7^ nitrogen in adenines may be important for some key interactions with initiation factors or ribosome. To gain insight into early *in vivo* stages post-transfection, we quantified the amount of selected representative **cap-mRNA_N** ^**R**^ by digital droplet PCR. All the transcriptionally active modified mRNAs, including **cap-mRNA_G** ^**H**^, were found to have good *in cellulo* stability comparable to nonmodified natural mRNA, indicating that the transfection and stability is not the reason for its superior translational efficiency. We assume that the reason for the positive effect of 7-deazaguanine may be in the altered formation of secondary structures and/or enhanced interactions with initiation factors or the ribosome, but this will need to be verified by further research.

Although these are just preliminary results based on one single reporter system and we cannot yet compare our results with established mRNA modifications, i.e., 1-methylpseudouridine^12^ that enhances translation through modulation of mRNA decoding,^13^ 7-deazaguanine clearly has a promising potential as a new mRNA modification for further research toward biotechnological or therapeutic applications. However, to fully establish the generality and robustness of the positive effect of 7-deazaguanine in translation, future studies will need to validate the modification across additional translation systems and with different reporter genes. In addition, systematic study of site-specific and segmental modification of different regions of the mRNA, such as the untranslated regions or coding sequence, will be important to dissect how positional effects influence translation efficiency and stability. These studies are now under way in our lab.

The set of base-modified **sgRNA_N** ^**R**^ was used for the study of their efficiency in CRISPR-Cas9 gene cleavage and their short-term stability in human serum. Modified **sgRNA_N** ^**R**^ bearing methyl groups and unsubstituted 7-deazapurines generally exhibit a higher stability than the ethyl derivatives, indicating that bulkier groups may negatively affect RNA secondary structures and therefore their stability toward nuclease degradation. The CRISPR-Cas cleavage activity experiments on **sgRNA_N** ^**R**^ revealed that most of these small modifications have a minimal impact on the efficiency of the gene cleavage. The modifications are generally tolerated, but none of them enhanced cleavage efficiency. However, we demonstrate that 7-methyl- and 7-ethyl-7-deazaadenines in **sgRNA_A** ^**Me**^ and **sgRNA_A** ^**Et**^ completely disrupt Cas9 nuclease activity most likely due to altering the structural conformation of sgRNA. Comparative molecular dynamics simulations of **sgRNA_A** ^**Me**^ and **sgRNA_A** ^**H**^ revealed a pronounced conformational change localized to Stem Loop 2 in the case of **sgRNA_A** ^**Me**^, leading to the loss of critical interactions between the guide RNA and key residues situated in the nuclease domain (RuvC II) of Cas9. These findings emphasize the importance of considering RNA structural dynamics in sgRNA engineering. Moving forward, our results suggest that future design of chemically modified sgRNAs must preserve this key structural motif and the importance for structure-informed design that will be essential for advancing CRISPR-based technologies, where sgRNA modifications are increasingly employed.

In this study, **cap-mRNA_N** ^**R**^ or **sgRNA_N** ^**R**^ was always uniformly modified at all positions containing the respective nucleobase. As a next step, we plan to employ the recently developed PEX protocol^15,33^ with TGK DNA polymerase to synthesize site-specifically and segmentally modified mRNAs and sgRNAs and study the influence of modification positioning on both translation and CRISPR-Cas9 cleavage activity. Understanding the effects caused by modified nucleotides might contribute to the future rational design of chemically modified RNAs for RNA-based therapeutics, including mRNA vaccines and/or genome editing applications.

## Experimental Section

A comprehensive description of the experimental procedures including the synthesis of novel modified nucleoside triphosphates, NMR and MS characterization data, detailed *in vitro* transcription protocols, RNA gel analysis, LC-MS data, and additional methods and notes is provided in the Supporting Information. The key procedures are outlined below.

### General Procedure for 5′-End Labeling via Ligation Reaction

The ligation reaction was performed in a total volume of 30 μL in T4 RNA ligase buffer (1×) and DMSO (10%) with either natural or modified RNA (1 μg), rATP (1 mM), pCp-ATTO-488 (1 μM), and T4 RNA ligase 1000 U/μL (1 μL). The mixture was incubated at 16 °C in a thermal cycler for 16 h. The mixture was dissolved in 20 μL of water and purified by a Monarch Kit (50 μg) following the supplier’s protocol. The samples were analyzed by gel electrophoresis on denaturing PAGE and visualized by fluorescence imaging using the Cy2 channel.

### *In Vitro* Transcription of 71-Mer Capped RNA

*In vitro* transcription reactions were performed using a HiScribe T7 High Yield RNA synthesis Kit. Each reaction was carried out in a final volume of 10 μL containing modified **N** ^**R**^ **TP** (6 mM), two natural NTPs (6 mM), natural GTP (2 mM), DMSO (5%), Ribolock RNase inhibitor (1 U/μL), dsDNA template (**70DNA_N** ^**R**^) (2 μM), T7 RNA polymerase (1 μL), and cap m^7^GpppA_m_pG (6 mM). For the negative control experiment, water was used instead of the solution of modified **N** ^**R**^ **TP**, while the positive control contained the natural NTP of interest (6 mM). The mixture was incubated at 37 °C for 3 h. The DNA template was then removed by treatment with DNase I (0.05 U/μL) for 30 min at 37 °C. EDTA (50 mM) was added, and samples were heated at 65 °C for 10 min before purification with Monarch RNA Cleanup Kit columns following the supplier’s protocol. Samples were labeled following the above-mentioned General Procedure for 5′-end labeling (GP-IV in the SI), analyzed by gel electrophoresis on 20% denaturing PAGE, and visualized by fluorescence imaging (Figure S3 in the SI). All samples were also characterized by LC-MS (Table S4, mass spectra in Figures S30–S41 in the SI).

### *In Vitro* Transcription of sgRNA Oligonucleotides

sgRNA targeting AAVS1 (sequence information in Section 6 in the SI) was synthesized by *in vitro* transcription using a HiScribe T7 High Yield RNA synthesis Kit. Reactions were performed in the total volume of 10 μL containing three natural NTPs (7.5 mM), one modified **N** ^**X**^ **TP** (7.5 mM), **sgRNA_N** ^**R**^ (0.5 μg), and T7 RNA polymerase (0.75 μL). For the negative control experiment, water was used instead of the solution of modified **N** ^**R**^ **TP**, while the positive control contained the natural NTP of interest (7.5 mM). The mixture was incubated at 37 °C for 4 h. The DNA template was then removed by treatment with DNase I (0.05 U/ μL) for 30 min at 37 °C. Subsequently, EDTA (50 mM) was added, and samples were heated at 65 °C for 10 min before purification with Monarch RNA Cleanup Kit columns following the supplier’s protocol and by HPLC (reverse-phase column; Biozen 2.6 μm oligo LC column 150 × 4.6 mm). Samples were labeled following GP-IV in the SI, analyzed by gel electrophoresis on 15% denaturing PAGE, and visualized by fluorescence imaging (Figure S5). All samples were also characterized by LC-MS (Table S5, mass spectra in Figures S42–S52 in the SI).

### *In Vitro* Transcription of mRNA

*In vitro* transcription reactions were performed using a HiScribe T7 High Yield RNA synthesis Kit in a final volume of 10 μL containing modified **N** ^**R**^ **TP** (10 mM), two natural NTPs (10 mM), natural GTP (2 mM), DMSO (5%), Ribolock RNase inhibitor (1 U/ μL), pDNA template **phRL-SV40-linear** (250 ng), T7 RNA polymerase (1 μL), and cap m7GpppA_m_pG (8 mM). For the negative control experiment, water was used instead of the solution of modified **N** ^**R**^ **TP**, while the positive control contained the natural NTP of interest (10 mM). The mixture was incubated at 37 °C for 4 h. The DNA template was then removed by treatment with DNase I (0.05 U/ μL) for 30 min at 37 °C. EDTA (50 mM) was added, and samples were heated at 65 °C for 10 min before purification with Monarch RNA Cleanup Kit columns following the supplier’s protocol. Samples were labeled following GP-IV in the SI, analyzed by gel electrophoresis on 7.5% denaturing PAGE, and visualized by fluorescence imaging (Figure S7 in the SI).

### *In Vitro* Translation Studies

Translation efficiency experiments were carried out in the Rabbit Reticulocyte Lysate System (RRLS). The reaction mixture (RRL, 7 μL) was supplemented with Complete Amino Acid mixture, 1 mM (0.5 μL), Ribolock RNase inhibitor, 40 U/μL (0.5 μL), and 50 ng of **cap-mRNA_N** ^**R**^. The reaction was incubated at 30 °C for 1.5 h and then stopped by freezing the samples at −80 °C. For luminescence detection, 2.5 μL of reaction was diluted with 2.5 μL of water in a 384-well plate. *Renilla* luciferase activity was measured using a Synergy H1 microplate reader (BioTek) following the injection of 40 μL of *Renilla* Luciferase buffer (Figure S13 in the SI). The data represent the results of two biological replicates, each performed in technical triplicate. Raw luminescence values were normalized to the signal obtained from the natural mRNA control (**cap-mRNA_nat**), which was set as 100%. The relative translation efficiency of the modified **cap-mRNA_N** ^**R**^ constructs was expressed as a percentage.

### Transfection of *Renilla* Luciferase mRNA

**cap-mRNA_nat** and **cap-mRNA_N** ^**R**^ were transfected to HeLa S3 cells using Lipofectamine MessengerMax transfection reagent according to the manufacturer’s instructions: One day before, the cells were transfected at 50% confluency in a 24-well plate. The transfection mixture per one well contained 70 ng of mRNA encoding for *Renilla* luciferase. Three hours after transfection, the transfection reagent was washed away by substituting the medium for fresh complete medium. At defined time points (3, 6, 12, 24, and 36 h post-transfection), cells were washed with 1× PBS and stored at −80 °C until further processing. For lysis, the cells were removed, refrozen, and lysed with 100 μL of PPBT lysis buffer (0.2% v/v Triton X-100, 100 mM potassium phosphate buffer, pH 7.8) per well for 10 min at RT with soft shaking. For luminescence measurement, 5 μL of the lysate was transferred into a 384-well plate. Using a TECAN Spark Multimode Microplate Reader, 40 μL of *Renilla* Luciferase buffer was injected and luminescence was measured (Figure S15 in the SI). The data shown represent the results of three biological replicates, each performed in technical triplicate. Raw luminescence values were normalized to the signal obtained from the natural mRNA control (**cap-mRNA_nat**) at 3 h, which was set as 100%. The relative translation efficiency of modified **cap-mRNA_N** ^**R**^ constructs was expressed as a percentage.

### Cotransfection of *Renilla* Luciferase mRNA with *Firefly* Luciferase

**cap-mRNA_nat** and **cap-mRNA_N** ^**R**^ were cotransfected with Firefly luciferase (**mRNA_natFF**) to HeLa S3 cells using Lipofectamine MessengerMax transfection reagent according to the manufacturer’s instructions: One day before, the cells were transfected at 50% confluency in a 24-well plate. The transfection mixture for one well contained 70 ng of **cap-mRNA_N** ^**R**^ and 50 ng of **mRNA_natFF**. Three hours after transfection, the transfection reagent was washed away by substituting the medium for fresh complete medium. At defined time points (3, 6, 12, 24, and 36 h post-transfection), cells were washed with 1× PBS and stored at −80 °C until further processing. For lysis, the plates were thawed on ice and cells were lysed by adding 100 μL of PPBT lysis buffer (0.2% v/v Triton X-100, 100 mM potassium phosphate buffer, pH 7.8) per well for 10 min at RT with soft shaking. For luminescence measurement, 5 μL of lysate was transferred into a 384-well plate. Using a TECAN Spark Multimode Microplate Reader, 40 μL of *Firefly* luciferase substrate was injected and luminescence was measured, followed by the addition of 40 μL of *Renilla* luciferase substrate that stops the FF luciferase activity and allows the assessment of *Renilla* luciferase expression (Figure S17). The data shown represent the results of three biological replicates, each performed in technical triplicate. Raw luminescence values were normalized to the signal obtained from the natural mRNA control encoding Firefly luciferase **mRNA_natFF** and then normalized to **cap-mRNA_nat** at 3 h, which was set as 100%. The relative translation efficiency of modified **cap-mRNA_N** ^**R**^ constructs was expressed as a percentage.

### CRISPR-Cas9 *in Vitro* DNA Cleavage with Modified sgRNAs

The reactions were carried out in PCR tubes placed in a thermocycler for precise heating control. A Cas9 master mix (for 10 reactions) was prepared by mixing 39 μL of water with 10 μL of buffer 3.1 (NEB) and 1 μL of a 20 mM solution of spCas9. Then, 5 μL of master mix (2 pmol) and 2.5 μL of 1 μM **sgRNA_N** ^**R**^ solution (2.5 pmol) were added to every PCR tube. The mixture was incubated for 10 min at 37 °C to form the Cas9-sgRNA complex. Next, 2.5 μL of 0.1 μM AAVS1 target DNA solution was added, and the reaction mixture was incubated for 30 min at 37 °C. The reaction was stopped by increasing the temperature to 65 °C for 5 min. Subsequently, 1 μL of RNase A/T1 mix was added, and the reaction mixture was incubated for 30 min at 37 °C, followed by addition of 1 μL of proteinase K and incubation for 15 min at 65 °C. For quantification of DNA fragments, on-chip electrophoresis was used.

### Stability Experiment in Human Serum

The stability of all labeled sgRNAs (prepared as described in Section 2.3.4.2 in the SI) was assessed in PCR tubes (25 μL final volume). First, **sgRNA_N** ^**R**^ (100 nM) was mixed with Cas9 nuclease (0.5 μM) and the mixture incubated at 37 °C for 10 min. Human serum (10%) and PBS (1×) were then added and the reaction was further incubated at 37 °C.

Samples were collected at 0 min, 15 min, 30 min, 1 h, and 2 h. At each time point, 5 μL of the reaction mixture was diluted in 5 μL of water and the solution was immediately flash-frozen in liquid nitrogen. Next, 1 μL of proteinase K was added, followed by the addition of 10 μL of loading dye, and the reaction was incubated for 15 min at 65 °C. The samples were analyzed by gel electrophoresis on 15% denaturing PAGE and visualized by fluorescence imaging (Figure S9 in the SI). Remaining sgRNA was quantified over time (Figure S10 in the SI), and the half-lives were calculated by fitting one phase decay model in GraphPad. Each analysis was done in triplicate (Figure S11 in the SI).

### Molecular Dynamics Simulations

The system building and simulation details are described in the SI, Sections 2.10.1 and 2.10.2, respectively. In short, three replicates for each system (**sgRNA_nat**, **sgRNA_A** ^**H**^, and **sgRNA_A** ^**Me**^) were prepared and simulated for 1 μs by using molecular dynamics.

The trajectories were analyzed using the MDAnalysis Python package.^56^ The per-residue root mean squared deviation (RMSD) of the RNA backbone was calculated using the phosphate atoms with respect to the reference PDB: 4OO8 after addition of hydrogens and missing loops. The contact probability between RNA residues 71–78 and S719, L720, H721, and E722 was calculated as the percentage of frames in which at least one atom from both selections is at 5 Å or less from another atom of the other selection. All of the results shown are average values between replicates. The simulation snapshots are presented using Pymol^57^ (Figure 7B, Figure S22 in the SI).

## Supplementary Material



## Data Availability

Raw data associated with this paper are deposited and freely available in a public repository: 10.48700/datst.b0x33-8ef95.

## Notes

Complete experimental procedures and characterization of compounds and modified RNAs, further data from translation and DNA cleavage experiments, molecular modeling, and copies of NMR and MS spectra (PDF)
