# Supplementary material for: Enzymatic Synthesis of Modified RNA Containing 5‑Methyl- or 5‑Ethylpyrimidines or Substituted 7‑Deazapurines and Influence of the Modifications on Stability, Translation, and CRISPR-Cas9 Cleavage
Source: ACS Chem Biol. 2025 Nov 6;20(11):2755–67. doi: 10.1021/acschembio.5c00692 (PMC12645431; doi:10.1021/acschembio.5c00692)
Supplement: Supplementary file 1 [file cb5c00692_si_001.pdf]

SUPPLEMENTARY INFORMATION

Enzymatic Synthesis of Modified RNA Containing 5-Methyl- or 5-Ethylpyrimidines or Substituted 7-Deazapurines and Influence of the Modifications on Stability, Translation and CRISPR-Cas9 Cleavage

Tania Sanchez-Quirante,[a,b] Erika Kužmová,[a] Miguel Riopedre-Fernandez,[a] Sebastian Golojuch,[c] Pavel Vopálenský,[a] Veronika Raindlová,[a] Afaf H. El-Sagheer,[c,d] Tom Brown,[c] Michal Hocek[a,b*]

[a] *Institute of Organic Chemistry and Biochemistry Czech Academy of Sciences. Flemingovo nam.2, 16610 Prague 6, Czech Republic*

[b] *Department of Organic Chemistry, Faculty of Science, Charles University in Prague, Hlavova 8, 12843 Prague 2, Czech Republic*

[c] *Department of Chemistry, Chemistry Research Laboratory, University of Oxford, Oxford OX1 3TA, UK*

[d] *School of Chemistry and Chemical Engineering, University of Southampton, Highfield, Southampton SO17 1BJ, U.K*

* *michal.hocek@uochb.cas.cz*

Contents

1 Synthetic part 5

1.1 General remarks 5

1.2 General synthetic scheme 6

1.3 General procedure (I) (GP-I) for Hydrogenation reaction 6

1.4 General procedure (II) (GP-II) for Triphosphorylation reactions 6

1.5 Chemical structures of all modified nucleosides and nucleoside triphosphates 7

1.6 Chemical synthesis of already published nucleosides and nucleotides 7

1.6.1 1-(β-D-ribofuranosyl) thymine (2, U^{Me}) 7

1.6.2 7-Methyl-7-deazaadenosine (3, A^{Me}) 7

1.6.3 7-Methyl-7-deazaguanosine (4, G^{Me}) 7

1.6.4 5-Methylcytidine 5'-*O*-triphosphate (5, C^{Me}TP) 8

1.6.5 5-Methyluridine 5'-*O*-triphosphate (6, U^{Me}TP) 8

1.6.6 7-Methyl-7-deazaadenosine 5'-*O*-triphosphate (7, A^{Me}TP) 8

1.6.7 7-Methyl-7-deazaguanosine- 5'-*O*-triphosphate (8, G^{Me}TP) 8

1.6.8 5-Ethylcytidine (13, C^{Et}) 9

1.6.9 5-Ethyluridine (14, U^{Et}) 9

1.6.10 7-Deazaadenosine (21, A^{H}) 10

1.6.11 7-Deazaguanosine (22, G^{H}) 10

1.6.12 7-Deazaadenosine 5'-*O*-triphosphate (23, A^{H}TP) 10

1.6.13 7-Deazaguanosine 5'-*O*-triphosphate (24, G^{H}TP) 11

1.7 Chemical synthesis of novel nucleosides and nucleotides 11

1.7.1 7-Ethyl-7-deazaadenosine (15, A^{Et}) 11

1.7.2 7-Ethyl-7-deazaguanosine (16, G^{Et}) 12

1.7.3 5-Ethylcytidine 5'-*O*-triphosphate (17, C^{Et}TP) 13

1.7.4 5-Ethyluridine 5'-*O*-triphosphate (18, U^{Et}TP) 14

1.7.5 7-Ethyl-7-deazaadenosine 5'-*O*-triphosphate (19, A^{Et}TP) 15

1.7.6 7-Ethyl-7-deazaguanosine 5'-*O*-triphosphate (20, G^{Et}TP) 16

2 Biochemical part 17

2.1 General remarks 17

2.2 List of DNA and RNA oligonucleotides used in this study 19

2.3 Enzymatic synthesis of modified RNA by *in vitro* transcription 22

2.3.1 General procedure (III) (GP-III) for annealing of dsDNA oligonucleotides used as a template for IVT 22
2.3.2 General procedure (IV) (GP-IV) for 5’-end labelling via ligation reaction....... 22
2.3.3 *In vitro* transcription of 71-mer capped RNA....... 22
2.3.4 Synthesis of modified 99-mer sgRNA oligonucleotides 25
2.4 Synthesis of modified cap-mRNA....... 27
2.5 CRISPR-Cas9 *in vitro* DNA cleavage experiments....... 30
2.5.1 Isolation of genomic DNA and PCR amplification of AAVS1 on-target 30
2.5.2 Automated on-chip electrophoresis 30
2.5.3 CRISPR-Cas9 *in vitro* DNA cleavage with modified sgRNAs....... 30
2.6 Stability of the sgRNAs in human serum 33
2.6.1 Stability experiment in human serum with Cas9 33
2.6.2 Stability experiment in human serum without Cas9 36
2.7 *In vitro* translation studies....... 36
2.7.1 10% SDS PAGE 38
2.8 *In cellulo* translation studies 38
2.8.1 Transfection of *Renilla* luciferase mRNA 38
2.8.2 RNA isolation and quantification by digital droplet PCR (ddPCR)....... 41
2.8.3 Transfection of *Renilla* luciferase mRNA with *Firefly* luciferase....... 42
2.8.4 Confluency analysis....... 44
2.9 RNA quantification....... 46
2.9.1 Synthesis of RNA by PEX....... 46
2.9.2 Fluorescence measurements....... 47
2.10 Molecular dynamics....... 49
2.10.1 Computational models 49
2.10.2 Simulation protocol....... 49
2.10.3 Simulation analysis 50

3 Copies of NMR spectra....... 52

3.1 ^{1}H and ^{13}C NMR spectra of 7-Ethyl-7-deazaadenosine (15, A^{Et})....... 52
3.2 ^{1}H and ^{13}C NMR spectra of 7-Ethyl-7-deazaguanosine (16, G^{Et})....... 53
3.3 ^{1}H, ^{13}C and ^{31}P {^{1}H} NMR spectra of 5-Ethylcytidine 5’-*O*-triphosphate (17, C^{Et}TP) 54

3.4 ^{1}H, ^{13}C and ^{31}P {^{1}H} NMR spectra of 5-Ethyluridine 5'-*O*-triphosphate (18, U^{Et}TP) 55
3.5 ^{1}H, ^{13}C and ^{31}P {^{1}H} NMR spectra of 7-Ethyl-7-deazaadenosine 5'-*O*-triphosphate (19, A^{Et}TP) 57
3.6 ^{1}H, ^{13}C and ^{31}P {^{1}H} NMR spectra of 7-Ethyl-7-deazaguanosine 5'-*O*-triphosphate (20, G^{Et}TP) 58

4 Copies of MS ESI- spectra of nucleosides and nucleotides 60

5 Copy of LC-ESI Spectra of Oligonucletotides 63

6 Appendix 86

6.1 Sequence of AAVS1 86
6.2 Sequences and map of plasmid 86
6.2.1 phRL-SV40 86
6.2.2 pGL4-CMV 88

7 References 92

1 Synthetic part

1.1 General remarks

All chemicals were purchased from commercial suppliers (Fluorochem, Sigma Aldrich). 5-iodocytidine was purchased from Biosynth Ltd., 5-iodouridine from Fluorochem, and 5-methylcytidine (**C^{Me} (1)**) from Sigma Aldrich. Phosphoryl chloride ($POCl_3$) and trimethyl phosphate [$PO(OMe)_3$] were distilled prior to use. Dried solvents were purchased from Acros Organics. The progress of reactions was monitored by silica gel thin-layer chromatography (TLC) in silica gel 60 F_{254} (Merck) plates with UV light detection at 254 and 365 nm. The masses of individual spots on TLC plate were measured by Advion Expression Compact Mass Spectrometer connected with Plate Express® TLC Plate Reader (TLC-MS) using electrospray ionization (ESI). Purification of the nucleosides was carried out on CombiFlash Rf+ with columns filled with Silica gel or by preparative flash chromatography on reverse phase (C18 RediSep column) both on a CombiFlash Teledyne ISCO system. Purification of the ribonucleoside triphosphates were performed using HPLC (Waters modular HPLC system) on a Phenomenex Kinetex 5 µm EVO C18 100 Å. Buffer A (0.1 M TEAB in H_2O) and buffer B (0.1 M TEAB in 50% MeOH) were used for purification of nucleoside triphosphates on RP-HPLC columns. NMR spectra were measured on a Bruker Avance III HD 500 MHz (^1H at 500.0 MHz, ^{13}C at 125.7 MHz and ^{31}P at 202.4 MHz) and JEOL ECZR 500 MHz (^1H at 500.2 MHz, ^{13}C at 125.8 MHz and ^{31}P at 202.5 MHz) in hexadeuteriodimethylsufoxide (DMSO-d6) or deuterium oxide (D_2O) referenced to the residual solvent signal. Chemical shifts are given in ppm (δ scale), coupling constants (*J*) in Hz. Low- and high-resolution mass spectra of small molecules were acquired on LTQ Orbitrap XL spectrometer (ESI ionization, Thermo Fisher Scientific). Mass of the small molecules were performed by the MS service at IOCB.

1.2 General synthetic scheme

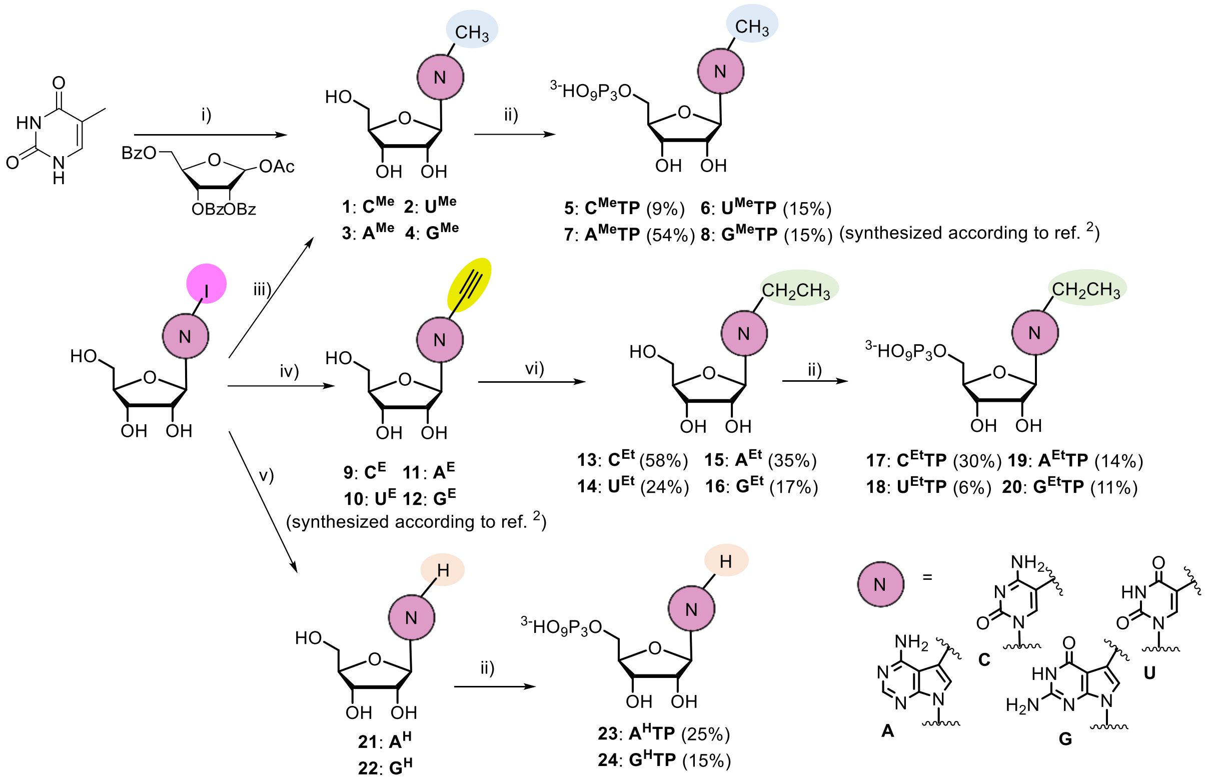

Figure S1: Synthesis of modified nucleosides and modified nucleoside triphosphates. Reagents and conditions: (i) TMSCl, HMDS, $SnCl_4$, 80 °C, (ii) 1. $POCl_3$, $PO(OMe)_3$, 0 °C, 2. $(NHBu_3)_2H_2P_2O_7$, Bu_3N, DMF, 0 °C 3. 2M TEAB. (iii) HMDS, 40 °C, $AlMe_3$, $Pd(PPh_3)_4$, THF, 80 °C, 2 h (iv) 1. TMS–acetylene, $[PdCl_2(PPh_3)]$ or $Pd(PPh_3)_4$, CuI, DMF, r.t., 16 h; 2. NH_3 (aq), Et_3N-3HF or K_2CO_3, 1-12 h, r.t; (v)/(vi) H_2 (1 atm.), 10% Pd/C, 5–12 h, 22 °C.

1.3 General procedure (I) (GP-I) for Hydrogenation reaction[1]

MeOH was added through a septum to an argon-purged flask containing the modified nucleoside and 10% Pd/C (1 equiv.) followed by vacuuming and fulfilling with H_2 atmosphere (balloon). The reaction mixture was stirred for 5–12 h at 22 °C. The resulting suspension was filtered with HPLC filter and/or purified by high performance reverse phase flash chromatography (RP-HPFC) (0 to 100% MeOH in H_2O).

1.4 General procedure (II) (GP-II) for Triphosphorylation reactions[2]

A modified nucleoside was heated under vacuum for 16 h at 60 °C. Then it was dissolved in dry $PO(OMe)_3$ (0.5 mL) under an argon atmosphere and cooled in an ice bath. $POCl_3$ (1.2 equiv.) was added through a septum and stirred for 1.5 h–5 h at 0 °C. In another argon purged flask, bis(tributylammonium) pyrophosphate ($(NHBu_3)_2H_2P_2O_7$) (5 equiv.) was dissolved in

dry MeCN (1 mL) and tributylamine (Bu_3N) and after cooling down in an ice bath, the mixture was added to the reaction. The reaction was stirred for 1 h at 0 °C and 2 M TEAB was added dropwise. The reaction was co-evaporated several times with water and the product was purified on RP-HPLC with use of a linear gradient of 0.1 M TEAB in H_2O to 0.1 M TEAB in 50% MeOH (1:1) as eluent, followed by freeze-drying from water.

1.5 Chemical structures of all modified nucleosides and nucleoside triphosphates

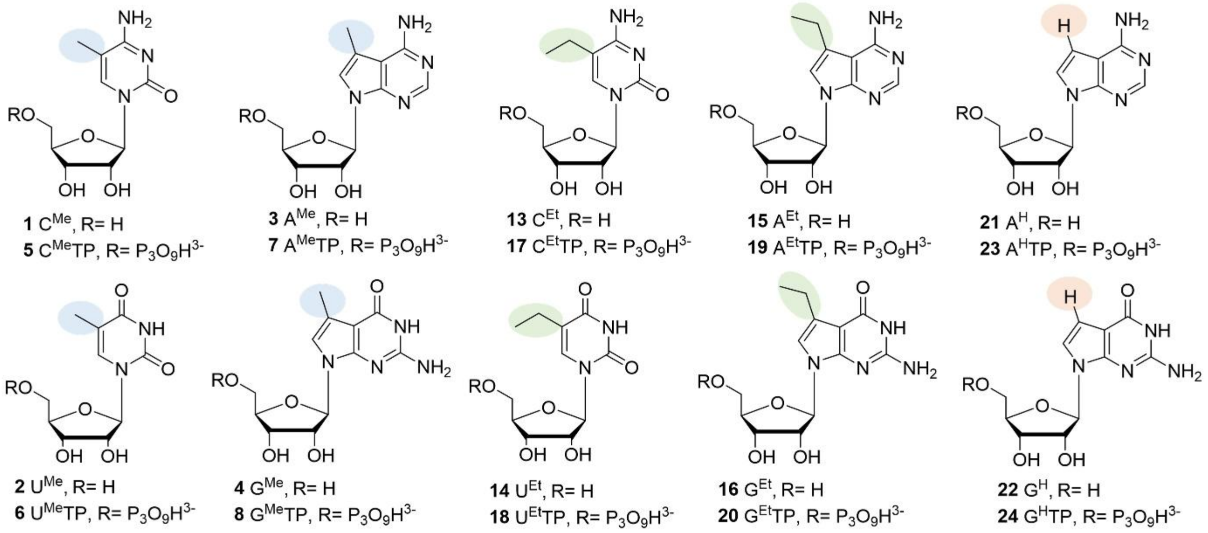

Figure S2: All modified nucleosides and nucleoside triphosphates involve/used in this study.

1.6 Chemical synthesis of already published nucleosides and nucleotides

1.6.1 1-(β-D-ribofuranosyl) thymine (2, U^{Me})

A literature procedure[3] starting from thymine (0.5 g, 4 mmol) gave product **U^{Me}** (**2**) (730 mg, 70% over 2 steps) as a white solid. The NMR spectra agreed with literature data.

1.6.2 7-Methyl-7-deazaadenosine (3, A^{Me})

The synthesis was performed following a published protocol.[2] Starting from 7-iodo-7-deazaadenosine[4] (300 mg, 0.76 mmol) product **A^{Me}** (**3**) (110 mg, 51%) was isolated as a white solid. The NMR spectra agreed with literature data.[2]

1.6.3 7-Methyl-7-deazaguanosine (4, G^{Me})

The synthesis was performed following a published protocol.[2] Starting from protected 7-iodo-7-deazaguanosine[5] (600 mg, 0.75 mmol) product **G^{Me}** (**4**) was isolated as a white solid (93 mg, 18%). The NMR spectra agreed with literature data.[2]

1.6.4 5-Methylcytidine 5'-*O*-triphosphate (5, C^{Me}TP)

The synthesis was performed according to General Procedure II (**GP-II**), starting from 5-methylcytidine (**1**, $\mathbf{C^{Me}}$) (80 mg, 0.31 mmol). Product $\mathbf{C^{Me}TP}$ (**5**) (25 mg, 9%) was isolated as a white lyophilizate. The NMR spectra agreed with literature data.[6]

1.6.5 5-Methyluridine 5'-*O*-triphosphate (6, U^{Me}TP)

The synthesis was performed according to **GP-II**, starting from 5-methyluridine (**2**, $\mathbf{U^{Me}}$) (100 mg, 0.39 mmol). Product $\mathbf{U^{Me}TP}$ (**6**) (45 mg, 15%) (triethylammonium salt) was isolated as a white lyophilizate.

^{1}H NMR (500.0 MHz, D_2O, ref(*t*BuOH) = 1.24 ppm): 1.27 (t, 36H, J_{vic} = 7.3, C**H**$_3$CH$_2$N); 1.93 (d, 3H, $J_{CH3,6}$ = 1.2, CH$_3$); 3.20 (q, 24H, J_{vic} = 7.3, CH$_3$C**H**$_2$N); 4.21 (ddd, 1H, J_{gem} = 11.4, $J_{H,P}$ = 4.3, $J_{5'b,4'}$ = 2.4, H-5′b); 4.27 (m, 1H, H-4′); 4.29 (ddd, 1H, J_{gem} = 11.4, $J_{H,P}$ = 6.0, $J_{5'b,4'}$ = 2.5, H-5′b); 4.43 (dd, 1H, $J_{2',1'}$ = 5.7, $J_{2',3'}$ = 5.3, H-2′); 4.50 (dd, 1H, $J_{3',2'}$ = 5.3, $J_{3',4'}$ = 3.8, H-3′); 6.00 (d, 1H, $J_{1',2'}$ = 5.7, H-1′); 7.81 (q, 1H, $J_{6,CH3}$ = 1.2, H-6).

^{13}C NMR (125.7 MHz, D_2O, ref(*t*BuOH) = 30.29 ppm): 8.92 (**C**H$_3$CH$_2$N); 12.39 (CH$_3$); 47.32 (CH$_3$**C**H$_2$N); 65.57 (d, $J_{C,P}$ = 5.5, CH$_2$-5′); 70.32 (CH-3′); 74.20 (CH-2′); 84.24 (d, $J_{C,P}$ = 9.1, CH-4′); 88.42 (CH-1′); 112.55 (C-5); 137.97 (CH-6); 152.85 (C-2); 167.42 (C-4).

^{31}P NMR (202.4 MHz, D_2O): –21.84 (dd, J = 20.7, 20.2, P_β); –10.93 (d, J = 20.2, P_α); –5.86 (d, J = 20.7, P_γ).

ESI MS *m/z* (rel. %): 417.0 (100) $[M - HPO_3]^-$; HR MS (ESI) for $C_{10}H_{16}O_{15}N_2P_3$ $[M - H]^-$: calcd 496.9769; found 496.9766.

1.6.6 7-Methyl-7-deazaadenosine 5'-*O*-triphosphate (7, A^{Me}TP)

The synthesis was performed according to **GP-II**, starting from 7-methyl-7-deazaadenosine (**3**, $\mathbf{A^{Me}}$) (50 mg, 0.18 mmol). Product $\mathbf{A^{Me}TP}$ (**7**) (88 mg, 54%) was isolated as a white lyophilizate. The NMR and MS spectra agreed with literature data.[2]

1.6.7 7-Methyl-7-deazaguanosine- 5'-*O*-triphosphate (8, G^{Me}TP)

The synthesis was performed according to **GP-II**, starting from 7-methyl-7-deazaguanosine (**4**, $\mathbf{G^{Me}}$) (50 mg, 0.16mmol). Product $\mathbf{G^{Me}TP}$ (**8**) (24 mg, 15%) was isolated as a white lyophilitate. The NMR and MS spectra agreed with literature data.[2]

1.6.8 5-Ethylcytidine (13, C^{Et})

Although $\mathbf{C^{Et}}$ has been previously described,[7] in this work we synthesized it using a distinct approach. 5-Iodocytidine (0.5 g, 1.35 mmol), CuI (180 mg, 0.945 mmol), and $Pd(PPh_3)_4$ (218 mg, 0.189 mmol) were dissolved in dry DMF (20 mL) and dry triethylamine (TEA) (18 mL). Then trimethylsilylacetylene (TMSA) (0.4 mL, 2.70 mmol) was added and the reaction was heated to 35 °C and stirred for 12 h. Volatiles were evaporated, and the crude product was purified by HPFC (13% MeOH in DCM). The resulting TMS-protected coupled product (380 mg, 1.12 mmol) was dissolved in aqueous ammonia (6 mL) and the solution was stirred at 22 °C for 1 h. The crude was purified by HPFC (20% MeOH in DCM). Using the resulting TMS-deprotected coupled product (**9**) (240 mg, 0.9 mmol) and following General Procedure I (**GP-I**) for 5 h, product $\mathbf{C^{Et}}$ (**13**) (211 mg, 58% over three steps) was isolated as a white solid.

^{1}H NMR (500.0 MHz, DMSO-d_6): 1.04 (t, 3H, J_{vic} = 7.4, C**H**$_3$CH$_2$); 2.27 (qd, 2H, J_{vic} = 7.4, ^4J = 0.9, CH$_3$C**H**$_2$); 3.54, 3.67 (2 × ddd, 2 × 1H, J_{gem} = 12.0, $J_{5',OH}$ = 5.1, $J_{5',4'}$ = 3.0, H-5′); 3.81 (dt, 1H, $J_{4',3'}$ = 4.9, $J_{4',5'}$ = 3.0, H-4′); 3.93 – 3.98 (m, 2H, H-2′,3′); 4.96 (d, 1H, $J_{OH,3'}$ = 4.8, OH-3′); 5.10 (t, 1H, $J_{OH,5'}$ = 5.1, OH-3′); 5.26 (d, 1H, $J_{OH,2'}$ = 4.8, OH-2′); 5.77 (m, 1H, H-1′); 6.82, 7.24 (2 × bs, 2 × 1H, NH$_2$); 7.70 (t, 1H, ^4J = 0.9, H-6).

^{13}C NMR (125.7 MHz, DMSO-d_6): 12.89 (**C**H$_3$CH$_2$); 20.05 (CH$_3$**C**H$_2$); 60.62 (CH$_2$-5′); 69.50 (CH-3′); 74.24 (CH-2′); 84.14 (CH-4′); 89.51 (CH-1′); 106.98 (C-5); 138.11 (CH-6); 155.39 (CH-2); 164.88 (C-4).

ESI MS *m/z* (rel. %): 272.1 (44.89) $[M + H]^+$; HR MS (ESI) for $C_{11}H_{18}O_5N_3$ $[M + H]^+$: calcd 272.1241; found 272.1242, for $C_{11}H_{17}O_5N_3Na$ $[M + Na]^+$: calcd 294.1060; found 294.1061.

1.6.9 5-Ethyluridine (14, U^{Et})

Although $\mathbf{U^{Et}}$ has been previously described,[1] in this work we synthesized it using a distinct approach. Copper(I) iodide (26 mg, 0.135 mmol), anhydrous TEA (5 mL), TMSA (2 mL), and $PdCl_2(PPh_3)_2$ (50 mg, 0.0675 mmol) were added to a degassed solution of iodinated nucleoside (0.5 g, 1.35 mmol) in anhydrous MeCN (10 mL). The mixture was stirred at 40 °C for 12 h. Resulting suspension was evaporated in vacuo, co-evaporated with silica and purified by HPFC (11% MeOH in DCM). The resulting TMS-protected coupled product (274 mg, 0.81 mmol) was dissolved in THF (7 mL), followed by addition of $Et_3N{\cdot}3HF$ (1.3 mL, 8.1 mmol). The reaction mixture was stirred for 12 h at 22 °C. The mixture was purified by HPFC (12% MeOH in DCM). Using resulting TMS-deprotected coupled product (**10**) (106 mg, 0.395 mmol) and

following **GP-I** for 12 h, product **U^{Et}** (**14**) (90 mg, 24% over 3 steps) was isolated as a yellow solid. The NMR spectra agreed with literature data.[1]

1.6.10 7-Deazaadenosine (21, A^{H})

Although **A^{H}** has been previously described,[4] in this work we synthesized it using a different approach following General Procedure I (**GP-I**). Starting from 7-iodo-7-deazaadenosine (350 mg, 0.89 mmol) product **A^{H}** (**21**) (58 mg, 24%) was isolated as a yellow solid. The NMR spectra agreed with literature data.[4]

1.6.11 7-Deazaguanosine (22, G^{H})

Although **G^{H}** has been previously described,[4] in this work we synthesized it using a different approach following **GP-I**. Starting from 7-iodo-7-deazaguanosine[5] (350 mg, 0.86 mmol) product **G^{H}** (**22**) (47 mg, 19%) was isolated as a white solid. The NMR spectra agreed with literature data.[4]

1.6.12 7-Deazaadenosine 5'-*O*-triphosphate (23, A^{H}TP)

The synthesis was performed according to **GP-II**, starting from 7-deazaadenosine (**21**, **A^{H}**) (55 mg, 0.2 mmol). Product **A^{H}TP** (**23**) (34 mg, 25 %) (triethylammonium salt) was isolated as a white lyophilizate.

^{1}H NMR (600.1 MHz, D_2O, ref(*t*BuOH) = 1.24 ppm): 1.21 (t, 27H, J_{vic} = 7.3, C**H**$_3$CH$_2$N); 3.11 (q, 18H, J_{vic} = 7.3, CH$_3$C**H**$_2$N); 4.14 (ddd, 1H, J_{gem} = 11.6, $J_{H,P}$ = 4.4, $J_{5'b,4'}$ = 3.0, H-5′b); 4.21 (ddd, 1H, J_{gem} = 11.6, $J_{H,P}$ = 6.0, $J_{5'b,4'}$ = 2.9, H-5′b); 4.30 (ddd, 1H, $J_{4',5'}$ = 3.0, 2.9, $J_{4',3'}$ = 2.7, H-4′); 4.51 (dd, 1H, $J_{3',2'}$ = 5.2, $J_{3',4'}$ = 2.7, H-3′); 4.65 (dd, 1H, $J_{2',1'}$ = 6.8, $J_{2',3'}$ = 5.2, H-2′); 6.19 (d, 1H, $J_{1',2'}$ = 6.8, H-1′); 6.65 (d, 1H, $J_{5,6}$ = 3.8, H-5); 7.55 (d, 1H, $J_{6,5}$ = 3.8, H-6); 8.08 (s, 1H, H-2).

^{13}C NMR (150.9 MHz, D_2O, ref(*t*BuOH) = 30.29 ppm): 8.88 (**C**H$_3$CH$_2$N); 47.27 (CH$_3$**C**H$_2$N); 65.21 (d, $J_{C,P}$ = 5.5, CH$_2$-5′); 71.46 (CH-3′); 74.65 (CH-2′); 84.37 (d, $J_{C,P}$ = 9.2, CH-4′); 86.53 (CH-1′); 102.10 (CH-5); 103.67 (C-4a); 123.41 (CH-6); 149.87 (C-7a); 150.06 (CH-2); 156.43 (C-4).

^{31}P NMR (202.5 MHz, D_2O): –22.23 (bt, J = 19.9, P_β); –10.66 (d, J = 19.9, P_α); –8.51 (bd, J = 19.9, P_γ).

ESI MS *m/z* (rel. %): 505.0 (100) [M – H]$^-$; 425.0 (92.23) [M – HPO$_3$]$^-$; HR MS (ESI) for $C_{11}H_{16}O_{13}N_4P_3$ [M – H]$^-$: calcd 504.9930; found 504.9932.

1.6.13 7-Deazaguanosine 5'-*O*-triphosphate (24, $G^{H}TP$)

The synthesis was performed according to **GP-II**, starting from 7-deazaguanosine (**22**, $\mathbf{G^{H}}$) (35 mg, 0.12 mmol). Product $\mathbf{G^{H}TP}$ (**24**) (32 mg, 28%) (triethylammonium salt) was isolated as a white lyophilizate.

^{1}H NMR (600.1 MHz, D_2O, ref(*t*BuOH) = 1.24 ppm): 1.26 (t, 27H, J_{vic} = 7.3, C**H**$_3$CH$_2$N); 3.17 (q, 18H, J_{vic} = 7.3, CH$_3$C**H**$_2$N); 4.15 (ddd, 1H, J_{gem} = 11.5, $J_{H,P}$ = 4.8, $J_{5'b,4'}$ = 3.4, H-5′b); 4.23 (ddd, 1H, J_{gem} = 11.5, $J_{H,P}$ = 6.1, $J_{5'b,4'}$ = 3.3, H-5′b); 4.30 (ddd, 1H, $J_{4',5'}$ = 3.4, 3.3, $J_{4',3'}$ = 2.6, H-4′); 4.52 (dd, 1H, $J_{3',2'}$ = 5.3, $J_{3',4'}$ = 2.6, H-3′); 4.69 (dd, 1H, $J_{2',1'}$ = 7.0, $J_{2',3'}$ = 5.3, H-2′); 6.05 (d, 1H, $J_{1',2'}$ = 7.0, H-1′); 6.58 (d, 1H, $J_{5,6}$ = 3.8, H-5); 7.19 (d, 1H, $J_{6,5}$ = 3.8, H-6).
^{13}C NMR (150.9 MHz, D_2O, ref(*t*BuOH) = 30.29 ppm): 8.93 (**C**H$_3$CH$_2$N); 47.34 (CH$_3$**C**H$_2$N); 66.31 (d, $J_{C,P}$ = 5.6, CH$_2$-5′); 71.44 (CH-3′); 74.06 (CH-2′); 84.19 (d, $J_{C,P}$ = 9.2, CH-4′); 86.48 (CH-1′); 101.14 (C-4a); 103.79 (CH-5); 119.83 (CH-6); 152.68 (C-7a); 153.44 (C-2); 162.10 (C-4).

^{31}P NMR (202.5 MHz, D_2O): –22.59 (dd, J = 20.1, 19.7, P_β); –10.76 (d, J = 20.1, P_α); –9.98 (bd, J = 19.7, P_γ).

ESI MS *m/z* (rel. %): 441.0 (100) [M – HPO$_3$]$^-$, 521.0 (83.53) [M – H]$^-$; HR MS (ESI) for $C_{11}H_{16}O_{14}N_4P_3$ [M – H]$^-$: calcd 520.9881; found 520.9879.

1.7 Chemical synthesis of novel nucleosides and nucleotides

1.7.1 7-Ethyl-7-deazaadenosine (15, A^{Et})

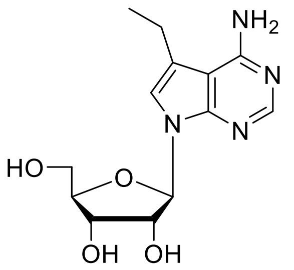

An argon purged mixture of 7-iodo-7-deazaadenosine[4] (150 mg, 0.383 mmol), PdCl$_2$(PPh$_3$) (14 mg, 0.02 mmol), CuI (8 mg, 0.038 mmol), TMSA (0.55 mL, 3.83 mmol) and TEA (0.5 mL) was stirred in DMF (3 mL) at 22 °C for 16 h. Resulting suspension was evaporated in vacuo, resuspended in MeOH, co-evaporated with silica gel and purified by HPFC (12% MeOH in DCM). The resulting TMS-protected coupled product (128 mg, 0.353 mmol) and K_2CO_3 (200

mg, 1.45 mmol) were dissolved in MeOH (5 mL) and was stirred at 22 °C for 2.5 h, followed by co-evaporation with celite. Product was purified by RP-HPFC (12% MeOH in H_2O). Using the resulting TMS-deprotected coupled product (**11**) (65 mg, 0.224 mmol) and following **GP-I** for 12 h, product **A^{Et}** (**15**) (40 mg, 35% over 3 steps) was isolated as a white solid.

^{1}H NMR (500.0 MHz, DMSO-d_6): 1.19 (t, 3H, J_{vic} = 7.4, C**H$_3$**CH$_2$); 2.77 (qd, 2H, J_{vic} = 7.4, ^4J = 1.2, CH$_3$C**H$_2$**); 3.51, 3.60 (2 × dd, 2 × 1H, J_{gem} = 11.9, $J_{5',4'}$ = 3.9, H-5′); 3.86 (td, 1H, $J_{4',5'}$ = 3.9, $J_{4',3'}$ = 3.2, H-4′); 4.06 (dd, 1H, $J_{3',2'}$ = 5.2, $J_{3',4'}$ = 3.2, H-3′); 4.37 (dd, 1H, $J_{2',1'}$ = 6.4, $J_{2',3'}$ = 5.2, H-2′); 5.22 (bs, 3H, OH-2′,3′,5′); 5.98 (d, 1H, $J_{1',2'}$ = 6.4, H-1′); 6.56 (bs, 2H, NH$_2$); 7.09 (t, 1H, ^4J = 1.2, H-6)); 8.01 (s, 1H, H-2).

^{13}C NMR (125.7 MHz, DMSO-d_6): 15.10 (**C**H$_3$CH$_2$); 19.37 (CH$_3$**C**H$_2$); 62.03 (CH$_2$-5′); 70.85 (CH-3′); 73.64 (CH-2′); 85.07 (CH-4′); 87.15 (CH-1′); 102.39 (C-4a); 117.04 (C-5); 118.71 (CH-6); 150.94 (C-7a); 151.47 (CH-2); 157.86 (C-4).

ESI MS *m/z* (rel. %): 295.14 (100) [*M* + H]$^+$; HR MS (ESI) for $C_{13}H_{19}O_4N_4$ [*M* + H]$^+$: calcd 295.1401; found 295.1402, for $C_{13}H_{18}O_4N_4Na$ [*M* + Na]$^+$: calcd 317.1220; found 317.1201.

1.7.2 7-Ethyl-7-deazaguanosine (16, G^{Et})

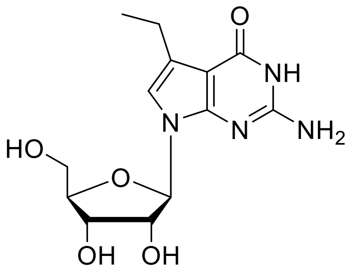

7-Iodo-7-deazaguanosine[5] (650 mg, 1.6 mmol), [Pd(PPh$_3$)$_4$] (186 mg, 0.16 mmol), and CuI (61 mg, 0.32 mmol) were dissolved in anhydrous DMF (25 mL) under argon atmosphere. Then TEA (0.4 mL, 4.46 mmol) and the TMSA (2 mL, 16 mmol) were added, and the mixture was stirred overnight at 22 °C. The crude mixture was purified by HPFC (15% DCM in MeOH). The resulting TMS-protected coupled product and the inseparable de-iodinated by-product were dissolved in 65 mL of MeOH and K_2CO_3 (380 mg, 2.75 mmol). The mixture was stirred overnight at 22 °C and purified by HPFC (19% DCM in MeOH). Using the resulting TMS-deprotected coupled product (**12**) (87 mg, 0.28 mmol) and following **GP-I** for 12 h, product **G^{Et}** (**16**) (84 mg, 17% over 3 steps) was isolated as a white solid.

^{1}H NMR (500.0 MHz, DMSO-d_6): 1.15 (t, 3H, J_{vic} = 7.5, C**H$_3$**CH$_2$); 2.57 (qd, 2H, J_{vic} = 7.5, $J_{CH2,6}$ = 1.1, CH$_3$C**H$_2$**); 3.47, 3.54 (2 × ddd, 2 × 1H, J_{gem} = 11.7, $J_{5',OH}$ = 5.5, $J_{5',4'}$ = 4.2, H-5′); 3.77 (td, 1H, $J_{4',5'}$ = 4.2, $J_{4',3'}$ = 3.2, H-4′); 3.99 (ddd, 1H, $J_{3',2'}$ = 5.2, $J_{3',OH}$ = 4.5, $J_{3',4'}$ = 3.2, H-

3′); 4.20 (td, 1H, $J_{2',1'} = J_{2',OH} = 6.6$, $J_{2',3'} = 5.2$, H-2′); 4.95 (t, 1H, $J_{OH,5'} = 5.5$, OH-5′); 5.01 (d, 1H, $J_{OH,3'} = 4.5$, OH-3′); 5.18 (d, 1H, $J_{OH,2'} = 6.5$, OH-2′); 5.82 (d, 1H, $J_{1',2'} = 6.5$, H-1′); 6.15 (bs, 2H, NH_2); 6.63 (t, 1H, $J_{6,CH2} = 1.1$, H-6); 10.24 (s, 1H, NH).

^{13}C NMR (125.7 MHz, DMSO-d_6): 14.93 (**C**H_3CH_2); 19.38 (**C**H_3CH_2); 61.83 (CH_2-5′); 70.62 (CH-3′); 73.37 (CH-2′); 84.43 (CH-4′); 85.64 (CH-1′); 99.05 (C-4a); 113.00 (CH-6); 121.05 (C-5); 151.48 (C-7a); 152.44 (C-2); 159.05 (C-4).

ESI MS *m/z* (rel. %): 333.1 (100) [*M* + Na]$^+$; HR MS (ESI) for $C_{13}H_{19}O_5N_4$ [*M* + H]$^+$: calcd 311.1350; found 311.1348; for $C_{13}H_{18}O_5N_4Na$ [*M* + Na]$^+$: calcd 333.1169; found 333.1165.

1.7.3 5-Ethylcytidine 5’-*O*-triphosphate (17, C^{Et}TP)

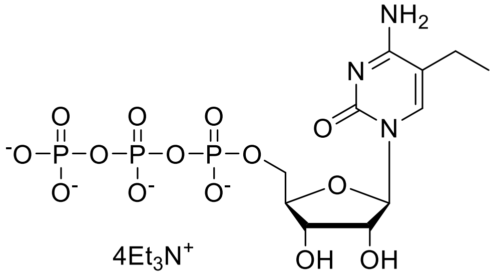

The synthesis was performed according to **GP-II**, starting from 7-ethylcytidine (**13**, **C^{Et}**) (50 mg, 0.16 mmol). Product **C^{Et}TP** (**17**) (50 mg, 30%) (triethylammonium salt) was isolated as a white lyophilizate.

^{1}H NMR (600.1 MHz, D_2O, ref (*t*BuOH) = 1.24 ppm): 1.13 (t, 3H, $J_{vic} = 7.5$, C**H**$_3CH_2$); 1.25 (t, 36H, $J_{vic} = 7.3$, C**H**$_3CH_2N$); 2.34 – 2.44 (m, 2H, CH_3C**H**$_2$); 3.17 (q, 24H, $J_{vic} = 7.3$, CH_3C**H**$_2N$); 4.19 – 4.28 (m, 3H, H-4′,5′); 4.35 (t, 1H, $J_{2',1'} = J_{2',3'} = 5.3$, H-2′); 4.42 (dd, 1H, $J_{3',2'} = 5.3$, $J_{3',4'} = 4.2$, H-3′); 6.01 (d, 1H, $J_{1',2'} = 5.3$, H-1′); 7.71 (t, 1H, $J_{6,CH2} = 1.0$, H-6).

^{13}C NMR (150.9 MHz, D_2O, ref (*t*BuOH) = 30.29 ppm): 8.91 (**C**H_3CH_2N); 12.77 (**C**H_3CH_2); 20.73 (CH_3**C**H_2); 47.29 (CH_3**C**H_2N); 65.67 (d, $J_{C,P} = 5.4$, CH_2-5′); 70.27 (CH-3′); 74.64 (CH-2′); 83.82 (d, $J_{C,P} = 9.3$, CH-4′); 89.34 (CH-1′); 111.48 (C-5); 138.54 (CH-6); 158.16 (C-2); 165.99 (C-4).

^{31}P NMR (202.5 MHz, D_2O): –22.51 (t, $J = 20.2$, P_β); –11.12 (d, $J = 20.2$, P_α); –8.73 (d, $J = 20.2$, P_γ).

ESI MS *m/z* (rel. %): 510.0 (100) [*M* – H]$^-$, 430.0 (51.69) [*M* – HPO_3]$^-$; HR MS (ESI) for $C_{11}H_{19}O_{14}N_3P_3$ [*M* – H]$^-$: calcd 510.0085; found 510.0083; for $C_{11}H_{18}O_{14}N_3NaP_3$ [*M* + Na – 2H]$^-$: calcd 531.9905; found 531.9896.

1.7.4 5-Ethyluridine 5'-*O*-triphosphate (18, U^{Et}TP)

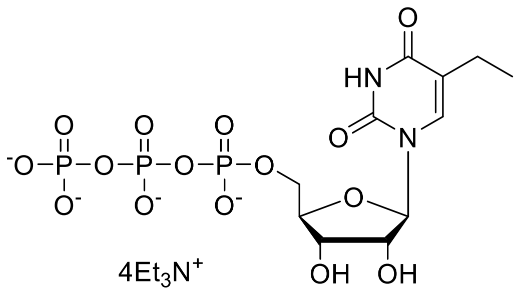

The synthesis was performed according to **GP-II**, starting from 7-ethyluridine (**14**, **U^{Et}**) (60 mg, 0.22 mmol). Product **U^{Et}TP** (**18**) (12 mg, 6%) (triethylammonium salt) was isolated as a white lyophilizate.

^{1}H NMR (500.0 MHz, D_2O, ref (*t*BuOH) = 1.24 ppm): 1.09 (t, 3H, J_{vic} = 7.5, C**H**$_3$CH$_2$); 1.25 (t, 36H, J_{vic} = 7.3, C**H**$_3$CH$_2$N); 2.35 (qd, 2H, J_{vic} = 7.5, $J_{CH2,6}$ = 1.0, CH$_3$C**H**$_2$); 3.15 (q, 24H, J_{vic} = 7.3, CH$_3$C**H**$_2$N); 4.18 (ddd, 1H, J_{gem} = 11.5, $J_{H,P}$ = 4.3, $J_{5'b,4'}$ = 2.5, H-5'b); 4.26 (m, 1H, H-4'); 4.29 (ddd, 1H, J_{gem} = 11.5, $J_{H,P}$ = 6.5, $J_{5'a,4'}$ = 2.9, H-5'a); 4.43 (dd, 1H, $J_{2',1'}$ = 6.0, $J_{2',3'}$ = 5.3, H-2'); 4.50 (dd, 1H, $J_{3',2'}$ = 5.3, $J_{3',4'}$ = 3.7, H-3'); 6.02 (d, 1H, $J_{1',2'}$ = 6.0, H-1'); 7.68 (t, 1H, $J_{6,CH2}$ = 1.0, H-6).

^{13}C NMR (125.7 MHz, D_2O, ref (*t*BuOH) = 30.29 ppm): 9.00 (**C**H$_3$CH$_2$N); 13.41 (**C**H$_3$CH$_2$); 20.77 (CH$_3$CH$_2$); 47.23 (CH$_3$**C**H$_2$N); 65.74 (d, $J_{C,P}$ = 5.5, CH$_2$-5'); 70.45 (CH-3'); 74.04 (CH-2'); 84.20 (d, $J_{C,P}$ = 9.1, CH-4'); 88.47 (CH-1'); 118.46 (C-5); 137.13 (CH-6); 155.10 (C-2); 169.93 (C-4).

^{31}P NMR (202.4 MHz, D_2O): –21.88 (t, J = 20.5, P$_\beta$); –10.97 (d, J = 20.5, P$_\alpha$); –5.70 (d, J = 20.5, P$_\gamma$).

ESI MS *m/z* (rel. %): 271.1 (100) [M – $H_3P_3O_9$]$^-$, 543.2 (49.07) [M – HPO_3 + Na]; HR MS (ESI) for $C_{11}H_{18}O_{15}N_2P_3$ [M – H]$^-$: calcd 510.9925; found 510.9921.

1.7.5 7-Ethyl-7-deazaadenosine 5'-*O*-triphosphate (19, A^{Et}TP)

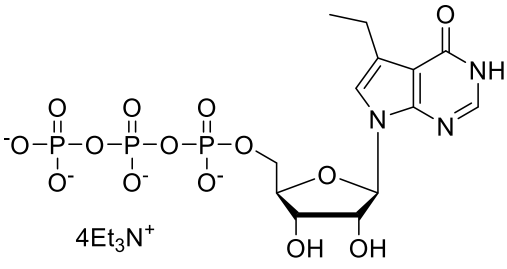

The synthesis was performed according to **GP-II**, starting from 7-ethyl-7-deazaadenosine (**15**, **A^{Et}**) (50 mg, 0.17 mmol). Product **A^{Et}TP** (**19**) (22 mg, 14%) (triethylammonium salt) was isolated as a white lyophilizate.

^{1}H NMR (500.0 MHz, D_2O, ref (*t*BuOH) = 1.24 ppm): 1.21 (t, 3H, J_{vic} = 7.5, C**H3**CH_2); 1.27 (t, 27H, J_{vic} = 7.3, C**H3**CH_2N); 2.69 (qd, 2H, J_{vic} = 7.5, $J_{CH2,6}$ = 1.1, CH_3C**H2**); 3.19 (q, 18H, J_{vic} = 7.3, CH_3C**H2**N); 4.12 (ddd, 1H, J_{gem} = 11.5, $J_{H,P}$ = 4.9, $J_{5'b,4'}$ = 3.6, H-5'b); 4.21 (ddd, 1H, J_{gem} = 11.5, $J_{H,P}$ = 6.2, $J_{5'a,4'}$ = 3.4, H-5'a); 4.27 (m, 1H, H-4'); 4.50 (dd, 1H, $J_{3',2'}$ = 5.3, $J_{3',4'}$ = 2.5, H-3'); 4.66 (dd, 1H, $J_{2',1'}$ = 7.1, $J_{2',3'}$ = 5.3, H-2'); 6.01 (d, 1H, $J_{1',2'}$ = 7.1, H-1'); 6.91 (t, 1H, $J_{6,CH2}$ = 1.1, H-6).

^{13}C NMR (125.7 MHz, D_2O, ref (*t*BuOH) = 30.29 ppm): 8.92 (**C**H_3CH_2N); 14.67 (**C**H_3CH_2); 19.83 (**C**H_3CH_2); 47.36 (CH_3**C**H_2N); 66.34 (d, $J_{C,P}$ = 5.8, CH_2-5'); 71.43 (CH-3'); 73.68 (CH-2'); 84.02 (d, $J_{C,P}$ = 9.2, CH-4'); 86.07 (CH-1'); 100.34 (C-4a); 115.06 (CH-6); 123.87 (C-5); 153.05 (C-7a); 153.42 (C-2); 162.24 (C-4).

^{31}P{^{1}H} NMR (202.4 MHz, D_2O): –21.91 (d, *J* = 20.6, 19.9, P_β); –10.73 (d, *J* = 19.9, P_α); –5.93 (d, *J* = 20.6, P_γ).

ESI MS *m/z* (rel. %): 453.1 (100) [*M* – HPO_3]$^-$, 533.0 (47.01) [*M* – H]$^-$; HR MS (ESI) for $C_{13}H_{20}O_{13}N_4P_3$ [*M* – H]$^-$: calcd 533.0245; found 533.0244.

1.7.6 7-Ethyl-7-deazaguanosine 5'-*O*-triphosphate (20, G^Et^TP)

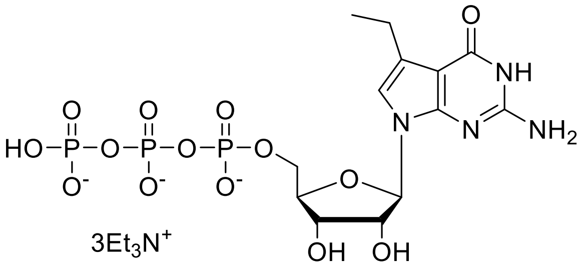

The synthesis was performed according to **GP-II**, starting from 7-ethyl-7-deazaguanosine (**16**, **G^{Et}**) (50 mg, 0.16 mmol). Product **G^{Et}TP** (**20**) (17 mg, 11%) (triethylammonium salt) was isolated as a white lyophilizate.

^{1}H NMR (500.0 MHz, D_2O, ref (*t*BuOH) = 1.24 ppm): 1.21 (t, 3H, J_{vic} = 7.5, C**H$_3$**CH$_2$); 1.27 (t, 27H, J_{vic} = 7.3, C**H$_3$**CH$_2$N); 2.69 (qd, 2H, J_{vic} = 7.5, $J_{CH2,6}$ = 1.1, CH$_3$C**H$_2$**); 3.19 (q, 18H, J_{vic} = 7.3, CH$_3$C**H$_2$**N); 4.12 (ddd, 1H, J_{gem} = 11.5, $J_{H,P}$ = 4.9, $J_{5'b,4'}$ = 3.6, H-5'b); 4.21 (ddd, 1H, J_{gem} = 11.5, $J_{H,P}$ = 6.2, $J_{5'a,4'}$ = 3.4, H-5'a); 4.27 (m, 1H, H-4'); 4.50 (dd, 1H, $J_{3',2'}$ = 5.3, $J_{3',4'}$ = 2.5, H-3'); 4.66 (dd, 1H, $J_{2',1'}$ = 7.1, $J_{2',3'}$ = 5.3, H-2'); 6.01 (d, 1H, $J_{1',2'}$ = 7.1, H-1'); 6.91 (t, 1H, $J_{6,CH2}$ = 1.1, H-6).

^{13}C NMR (125.7 MHz, D_2O, ref (*t*BuOH) = 30.29 ppm): 8.92 (**C**H$_3$CH$_2$N); 14.67 (**C**H$_3$CH$_2$); 19.83 (**C**H$_3$CH$_2$); 47.36 (CH$_3$**C**H$_2$N); 66.34 (d, $J_{C,P}$ = 5.8, CH$_2$-5'); 71.43 (CH-3'); 73.68 (CH-2'); 84.02 (d, $J_{C,P}$ = 9.2, CH-4'); 86.07 (CH-1'); 100.34 (C-4a); 115.06 (CH-6); 123.87 (C-5); 153.05 (C-7a); 153.42 (C-2); 162.24 (C-4).

^{31}P{^{1}H} NMR (202.4 MHz, D_2O): –22.60 (d, J = 20.2, 19.6, P$_\beta$); –10.80 (d, J = 20.2, P$_\alpha$); –10.15 (d, J = 19.6, P$_\gamma$).

ESI MS *m/z* (rel. %): 549.0 (100) [M – H]$^-$, 469.1 (85.53) [M – HPO$_3$]$^-$, 550.0 (40.39) [M]; HR MS (ESI) for $C_{13}H_{20}O_{14}N_4P_3$ [M – H]$^-$: calcd 549.0194; found 549.0191, for $C_{13}H_{19}O_{14}N_4NaP_3$ [M + Na – 2H]$^-$: calcd 571.0014; found 571.0010.

2 Biochemical part

2.1 General remarks

RNase/DNase free solutions for biochemical reactions were prepared using Milli-Q water treated with DEPC and sterilized by autoclaving. Single-stranded DNA oligonucleotides for preparation of double-stranded DNA templates were purchased from Generi Biotech, Eurofins or IDT. DNase I, TurboDNase, RiboLock RNase Inhibitor, 50 mM EDTA, Lipofectamine MessengerMAX, Opti-MEM medium, RNAse A/T1 mix and SYBR gold (10,000X in DMSO were purchased from Thermo Fisher Scientific. HiScribe T7 High-Yield RNA Synthesis Kit, Monarch RNA purification kits (50 µg), Proteinase K, BamHI-HF and SalI restriction enzyme, Gel loading dye purple (6X), Q5 High-Fidelity DNA polymerase and Color Prestained Protein Standar, Broad Range (10-250 kDa ladder) were purchased from New England Biolabs. RiboRuler High Range RNA ladder, RiboRuler Low Range, Ultra Low Range DNA ladder, GeneRuler 1 kb DNA ladder, as well as Lipofectamine MessengerMAX, were obtained from Thermo Fisher Scientfic. DNA ladder was in-house prepared by mixing FAM-labeled oligonucleotides of 100, 80, 60 nucleotides. PcP-ATTO488 was purchased from Jena Bioscience. The enzymes 5' Polyphosphatase and Terminator 5'-Phosphate-Dependent Exonuclease were purchased in Biosearch Technologies. Human Serum from Sigma Aldrich. Coelenterazine h was purchased from Nanolight Technologies. Rabbit reticulocyte Lysate system (RRLS), FluroTech Green Lys and Complete Amino Acid Mixture (1 mM) and phRL-SV40 (# E2231) from Promega. *Renilla* Luciferase buffer was prepared (25 mM Na_4PP_i, 10 mM NaOAc, 15 mM EDTA, 500 mM Na_2SO_4, 500 mM NaCl, 1.3 mM NaN_3, 4 µM coelenterazine h). RNA isolation was performed using Quick RNA microprep kit (Zymo) and reverse transcription using Quantitect Reverse Transcription Kit from Qiagen.

PCR products were purified using QIAquick PCR Purification Kit (Qiagen). All samples were analysed by denaturing PAGE (acrylamide/bisacrylamide 19:1, 7M urea) in 1X TBE pH 8 under denaturing conditions (50 °C, 30 mA) using 2X Gel Loading Dye (80% [v/v] formamide, 20 mM EDTA, 0.025% [w/v] bromophenol blue, 0.0250% [w/v] xylene cyanol and 0.025% SDS) or by agarose gel (Serva) containing Gel Red (Biotium, 10,000X in water) in 0.5X TBE buffer pH 8, using 6X DNA Gel Loading Dye (60 mM EDTA, 10 mM Tris-HCl (pH 7.6), 60% glycerol, 0.03% bromophenol blue, 0.03% xylene cyanol FF, ThermoFisher Scientific).

PAGE and agarose gels were analyzed by fluorescence imaging using a Typhoon FLA9500 (GE Healthcare Life Sciences) or an Amersham Typhoon (Cytiva). Samples were concentrated

on CentriVap Vacuum Concentrator system (Labconco). Concentrations were measured by Nanophotometer N60 (Implen). Luminescence was measured in TECAN Spark® Multimode Microplate Reader. Quantification from dPAGE gels was carried out by ImageJ.

Purification of the sgRNA was performed using semi-preparative HPLC (Waters modular HPLC system) on a column packed with C18 reverse phase (Biozen 2.6 µm oligo LC column 150 x 4.6 mm) using mobile phase A (100 mM TEAAc in H_2O) and mobile phase B (100 mM TEAAc in 50 % MeCN) by 20 min gradient from 0 % B to 35 % B. LC-ESI-MS spectra were acquired on Agilent 1290 Infinity II Bio system with DAD detector and mass spectrometer MSD XT. LC-ESI-MS analysis of oligonucleotides was carried out according to standard procedures using mobile phases A (12.2 mM Et_3N, 300 mM HFIP in H_2O) and B (12.2 mM Et_3N, 300 mM HFIP in H_2O in 100% MeOH) by 10 min gradient from 5% B to 100% B in A using BioZen 1.7 µm oligo column 2.1 x 50 mm (Phenomenex). Deconvolutions of LC-ESI-MS spectra were carried out using UniDec program[8].

HEK-293 cell lines (ATCC #CRL-1573) was a gift from Elizabeth J. Robertson (Sir William Dunn School of Pathology, University of Oxford).

HeLa S3 cell line (ATCC # CCL-2.2) was cultured and maintained at 37°C, 5% CO_2 in DMEM high glucose 4.5 g/L medium (cat. #: R8758, Merck, Germany). The medium was supplemented with 10% fetal bovine serum (cat. #: F7524, Merck, Germany; inactivated at 56 °C for 30 min), 1% of L-glutamine (cat. #: G7513, Merck, Germany) and 1% of Penicillin/Streptomycin (Sigma-Aldrich #P4333-100ml). For experiments the cells were seeded in 24 well plates (cat #: 10062-896, VWR International). For measurements of luminescence 384-well format white micro plates (cat. #: 164610, Nunc, Thermo Fisher Scientific, USA) were used.

The cells were negatively tested for the presence of mycoplasma using MycoAlert Mycoplasma Detection kit (cat. #: LT07-318, Lonza).

Cell growth was measured by IncuCyte® SX1 Live-Cell Analysis System (Sartorius). For ddPCR QX200 Droplet Digital PCR System (Bio-Rad) with automated Droplet Generator (Bio-Rad) and QX200 Droplet Reader was used. Aabsolute transcript copy numbers were calculated using QuantaSoft analysis software.

2.2 List of DNA and RNA oligonucleotides used in this study

Table S1 List of DNA templates and primers

Name		Size (nt)	Sequence (5´-3´ direction)
**70DNA_N^{R}**	sense	87	TAATACGACTCACTATAGGGCCCTTCGCCAACTTGCAGAGACGGTCGGGTCCAGATATTCGTATCTGTCGAGTAGAGTGTGGGCTCC
	anti-sense	87	[mG][mG]AGCCCACACTCTACTCGACAGATACGAATATCTGGACCCGACCGTCTCTGCAAGTTGGCGAAGGGCCCTATAGTGAGTCGTATTA
**sgDNA_N^{R}**	sense	118	TCTAATACGACTCACTATAGGGGCCACTAGGGACAGGATGTTTTAGAGCTAGAAATAGCAAGTTAAAATAAGGCTAGTCCGTTATCAACTTGAAAAAGTGGCACCGAGTCGGTGCTTT
	anti-sense	118	[mA][mA]AGCACCGACTCGGTGCCACTTTTTCAAGTTGATAACGGACTAGCCTTATTTTAACTTGCTATTTCTAGCTCTAAAACATCCTGTCCCTAGTGGCCCCTATAGTGAGTCGTATTAGA
**AAVS1_FWR**	forward primer	20	CCCCGTTCTCCTGTGGATTC
**AAVS1_REV**	reverse primer	20	CTCCCTCCCAGGATCCTCTC
**PrimFWR_sgRNA99**	forward primer	22	[mA][mA]AGCACCGACTCGGTGCCACT
**PrimREV_sgRNA99**	reverse primer	28	TCTAATACGACTCACTATAGGGGCCACT
**hRL_Fwd**	forward primer	19	CGTCGTGCCTCACATCGAG
**hRL_Rev**	reverse primer	19	ATCCAGGAGGCGATATGAG
**LT25TH-Cy5**	primer	26	CAAGGACAAAATACCTGTATTCCTT
**FVL-A**	template	98	GACATCATGAGAGACATCGCCTCTGGGCTAATAGGACTACTTCTAATCTGTAAGAGCAGATCCCTGGACAGGCAAGGAATACAGGTATTTTGTCCTTG

Notes: Promoter region is underlined.

The last one or two nucleotides at the 5´-terminus in the anti-sense strand and forward primers are 2´-*O*-Me modified [mN; N = A or G] to minimize non-templated nucleotide addition[9].

Table S2 List of RNA oligonucleotides synthesized in this study

Name	Size (nt)	Sequence (5′-3′ direction)
**70RNA_uncap**	70	GGGCCCUUCGCCAACUUGCAGAGACGGUCGGGUCCAGAUAUUCGUAUCUGUCGAGUAGAGUGUGGGCUCC
**cap71RNA_nat**	71	CapGGCCCUUCGCCAACUUGCAGAGACGGUCGGGUCCAGAUAUUCGUAUCUGUCGAGUAGAGUGUGGGCUCC
**cap71RNA_C^{Me}**	71	CapGGC^{Me}C^{Me}C^{Me}UUC^{Me}GC^{Me}C^{Me}AAC^{Me}UUGC^{Me}AGAGAC^{Me}GGUC^{Me}GGGUC^{Me}C^{Me}AGAUAUUC^{Me}GUAUC^{Me}UGUC^{Me}GAGUAGAGUGUGGGC^{Me}UC^{Me}C^{Me}
**cap71RNA_C^{Et}**	71	CapGGC^{Et}C^{Et}C^{Et}UUC^{Et}GC^{Et}C^{Et}AAC^{Et}UUGC^{Et}AGAGAC^{Et}GGUC^{Et}GGGUC^{Et}C^{Et}AGAUAUUC^{Et}GUAUC^{Et}UGUC^{Et}GAGUAGAGUGUGGGC^{Et}UC^{Et}C^{Et}
**cap71RNA_U^{Me}**	71	CapGGCCCU^{Me}U^{Me}CGCCAACU^{Me}U^{Me}GCAGAGACGGU^{Me}CGGGU^{Me}CCAGAU^{Me}AU^{Me}U^{Me}CGU^{Me}AU^{Me}CU^{Me}GU^{Me}CGAGU^{Me}AGAGU^{Me}GU^{Me}GGGCU^{Me}CC
**cap71RNA_U^{Et}**	71	CapGGCCCU^{Et}U^{Et}CGCCAACU^{Et}U^{Et}GCAGAGACGGU^{Et}CGGGU^{Et}CCAGAU^{Et}AU^{Et}U^{Et}CGU^{Et}AU^{Et}CU^{Et}GU^{Et}CGAGU^{Et}AGAGU^{Et}GU^{Et}GGGCU^{Et}CC
**cap71RNA_A^{H}**	71	CapGGCCCUUCGCCA^{H}A^{H}CUUGCA^{H}GA^{H}GA^{H}CGGUCGGGUCCA^{H}GA^{H}UA^{H}UUCGUA^{H}UCUGUCGA^{H}GUA^{H}GA^{H}GUGUGGGCUCC
**cap71RNA_A^{Me}**	71	CapGGCCCUUCGCCA^{Me}A^{Me}CUUGCA^{Me}GA^{Me}GA^{Me}CGGUCGGGUCCA^{Me}GA^{Me}UA^{Me}UUCGUA^{Me}UCUGUCGA^{Me}GUA^{Me}GA^{Me}GUGUGGGCUCC
**cap71RNA_A^{Et}**	71	CapGGCCCUUCGCCA^{Et}A^{Et}CUUGCA^{Et}GA^{Et}GA^{Et}CGGUCGGGUCCA^{Et}GA^{Et}UA^{Et}UUCGUA^{Et}UCUGUCGA^{Et}GUA^{Et}GA^{Et}GUGUGGGCUCC
**cap71RNA_G^{H}**	71	CapG^{H}G^{H}CCCUUCG^{H}CCAACUUG^{H}CAG^{H}AG^{H}ACG^{H}G^{H}UCG^{H}G^{H}G^{H}UCCAG^{H}AUAUUCG^{H}UAUCUG^{H}UCG^{H}AG^{H}UAG^{H}AG^{H}UG^{H}UG^{H}G^{H}G^{H}CUCC
**cap71RNA_G^{Me}**	71	CapG^{Me}G^{Me}CCCUUCG^{Me}CCAACUUG^{Me}CAG^{Me}AG^{Me}ACG^{Me}G^{Me}UCG^{Me}G^{Me}G^{Me}UCCAG^{Me}AUAUUCG^{Me}UAUCUG^{Me}UCG^{Me}AG^{Me}UAG^{Me}AG^{Me}UG^{Me}UG^{Me}G^{Me}G^{Me}CUCC
**cap71RNA_G^{Me}**	71	CapG^{Et}G^{Et}CCCUUCG^{Et}CCAACUUG^{Et}CAG^{Et}AG^{Et}ACG^{Et}G^{Et}UCG^{Et}G^{Et}G^{Et}UCCAG^{Et}AUAUUCG^{Et}UAUCUG^{Et}UCG^{Et}AG^{Et}UAG^{Et}AG^{Et}UG^{Et}U G^{Et}G^{Et}G^{Et}CUCC
**sgRNA_nat**	99	pppGGGGCCACUAGGGACAGGAUGUUUUAGAGCUAGAAAUAGCAAGUUAAAAUAAGGCUAGUCCGUUAUCAACUUGAAAAAGUGGCACCGAGUCGGUGCUUU
**sgRNA_C^{Me}**	99	pppGGGGC^{Me}C^{Me}AC^{Me}UAGGGAC^{Me}AGGAUGUUUUAGAGC^{Me}UAGAAAUAGC^{Me}AAGUUAAAAUAAGGC^{Me}UAGUC^{Me}C^{Me}GUUAUC^{Me}AAC^{Me}UUGAAAAAGUGGC^{Me}AC^{Me}C^{Me}GAGUC^{Me}GGUGC^{Me}UUU

**sgRNA_C^{Et}**	99	pppGGGG$C^{Et}C^{Et}$AC^{Et}UAGGGAC^{Et}AGGAUGUUUUAGAGC^{Et}UA<br>GAAAUAGC^{Et}AAGUUAAAAUAAGGC^{Et}UAGU$C^{Et}C^{Et}$GUUAU<br>C^{Et}AAC^{Et}UUGAAAAAGUGGC^{Et}A$C^{Et}C^{Et}$GAGUC^{Et}GGUGC^{Et}UUU
**sgRNA_U^{Me}**	99	pppGGGGCCACU^{Me}AGGGACAGGAU^{Me}G$U^{Me}U^{Me}U^{Me}U^{Me}$AGAG<br>CU^{Me}AGAAAU^{Me}AGCAAG$U^{Me}U^{Me}$AAAAU^{Me}AAGGCU^{Me}AGU^{Me}<br>CCG$U^{Me}U^{Me}$AU^{Me}CAAC$U^{Me}U^{Me}$GAAAAAGU^{Me}GGCACCGAG<br>U^{Me}CGGU^{Me}GC$U^{Me}U^{Me}U^{Me}$
**sgRNA_U^{Et}**	99	pppGGGGCCACU^{Et}AGGGACAGGAU^{Et}G$U^{Et}U^{Et}U^{Et}U^{Et}$AGAGCU^{Et}<br>AGAAAU^{Et}AGCAAG$U^{Et}U^{Et}$AAAAU^{Et}AAGGCU^{Et}AGU^{Et}CCGU^{Et}<br>U^{Et}AU^{Et}CAAC$U^{Et}U^{Et}$GAAAAAGU^{Et}GGCACCGAGU^{Et}CGGU^{Et}GC<br>$U^{Et}U^{Et}U^{Et}$
**sgRNA_A^{H}**	99	pppGGGGCCA^{H}CUA^{H}GGGA^{H}CA^{H}GGA^{H}UGUUUUA^{H}GA^{H}GCU<br>A^{H}G$A^{H}A^{H}A^{H}$UA^{H}GC$A^{H}A^{H}$GUU$A^{H}A^{H}A^{H}A^{H}$U$A^{H}A^{H}$GGCUA^{H}GUC<br>CGUUA^{H}UC$A^{H}A^{H}$CUUG$A^{H}A^{H}A^{H}A^{H}A^{H}$GUGGC$A^{H}CCGA^{H}$GUCG<br>GUGCUUU
**sgRNA_A^{Me}**	99	pppGGGGCCA^{Me}CUA^{Me}GGGA^{Me}CA^{Me}GGA^{Me}UGUUUUA^{Me}GA^{Me}<br>GCUA^{Me}G$A^{Me}A^{Me}A^{Me}$UA^{Me}GC$A^{Me}A^{Me}$GUU$A^{Me}A^{Me}A^{Me}A^{Me}$UA^{Me}<br>A^{Me}GGCUA^{Me}GUCCGUUA^{Me}UC$A^{Me}A^{Me}$CUUG$A^{Me}A^{Me}A^{Me}A^{Me}$<br>A^{Me}GUGGCA^{Me}CCGA^{Me}GUCGGUGCUUU
**sgRNA_A^{Et}**	99	pppGGGGCCA^{Et}CUA^{Et}GGGA^{Et}CA^{Et}GGA^{Et}UGUUUUA^{Et}GA^{Et}GCU<br>A^{Et}G$A^{Et}A^{Et}A^{Et}$UA^{Et}GC$A^{Et}A^{Et}$GUU$A^{Et}A^{Et}A^{Et}A^{Et}$U$A^{Et}A^{Et}$GGCUA^{Et}G<br>UCCGUUA^{Et}UC$A^{Et}A^{Et}$CUUG$A^{Et}A^{Et}A^{Et}A^{Et}A^{Et}$GUGGC$A^{Et}CCGA^{Et}$<br>GUCGGUGCUUU
**sgRNA_G^{H}**	99	ppp$G^{H}G^{H}G^{H}G^{H}$CCACUA$G^{H}G^{H}G^{H}$ACA$G^{H}G^{H}$AUG^{H}UUUUAG^{H}A<br>G^{H}CUAG^{H}AAAUAG^{H}CAAG^{H}UUAAAAUAA$G^{H}G^{H}$CUAG^{H}UCC<br>G^{H}UUAUCAACUUG^{H}AAAAAG^{H}U$G^{H}G^{H}$CACCG^{H}AG^{H}UC$G^{H}G^{H}$U<br>G^{H}CUUU
**sgRNA_G^{Me}**	99	ppp$G^{Me}G^{Me}G^{Me}G^{Me}$CCACUA$G^{Me}G^{Me}G^{Me}$ACA$G^{Me}G^{Me}$AUG^{Me}UUU<br>UAG^{Me}AG^{Me}CUAG^{Me}AAAUAG^{Me}CAAG^{Me}UUAAAAUAA$G^{Me}G^{Me}$<br>CUAG^{Me}UCCG^{Me}UUAUCAACUUG^{Me}AAAAAG^{Me}U$G^{Me}G^{Me}$CAC<br>C G^{Me}AG^{Me}UC$G^{Me}G^{Me}$UG^{Me}CUUU
**sgRNA_G^{Et}**	99	ppp$G^{Et}G^{Et}G^{Et}G^{Et}$CCACUA$G^{Et}G^{Et}G^{Et}$ACA$G^{Et}G^{Et}$AUG^{Et}UUUUAG^{Et}<br>AG^{Et}CUAG^{Et}AAAUAG^{Et}CAAG^{Et}UUAAAAUAA$G^{Et}G^{Et}$CUAG^{Et}UC<br>CG^{Et}UUAUCAACUUG^{Et}AAAAAG^{Et}U$G^{Et}G^{Et}$CACCG^{Et}AG^{Et}UCG^{Et}<br>G^{Et}UG^{Et}CUUU
**98RNA_nat**	98	Cys5'CAAGGACAAAAUACCUGUAUUCCUUGCCUGUCCAG<br>GGAUCUCGUCUUACAGAUUAGAAGUAGUCCUAUUAGCC<br>CAGAGGCGAUGUCUCUCAUGAUGUC
**98RNA_AH**	98	Cys5'C$A^{H}A^{H}$GGA^{H}C$A^{H}A^{H}A^{H}A^{H}$UA^{H}CCUGUA^{H}UUCCUUG<br>CCUGUCCA^{H}GGGA^{H}UCUCGUCUUA^{H}CA^{H}GA^{H}UUA^{H}G$A^{H}A^{H}$<br>GUA^{H}GUCCUA^{H}UUA^{H}GCCCA^{H}GA^{H}GGCGA^{H}UGUCUCUC<br>A^{H}UGA^{H}UGUC
**98RNA_GH**	98	Cys5'CAA$G^{H}G^{H}$ACAAAAUACCUG^{H}UAUUCCUUG^{H}CCUG^{H}<br>UCCA$G^{H}G^{H}G^{H}$AUCUCG^{H}UCUUACAG^{H}AUUAG^{H}AAG^{H}UAG^{H}<br>UCCUAUUAG^{H}CCCAG^{H}A$G^{H}G^{H}$CG^{H}AUG^{H}UCUC<br>UCAUG^{H}AUG^{H}UC

Notes: ppp (triphosphate residue) marked in blue, cap (m^7GpppAmpG) marked in purple AND Cys5' marked in green at the 5' terminus. Modified bases in transcripts are marked in red.

2.3 Enzymatic synthesis of modified RNA by *in vitro* transcription

2.3.1 General procedure (III) (GP-III) for annealing of dsDNA oligonucleotides used as a template for IVT

A solution of complementary single-stranded DNA oligonucleotide (sense and antisense) (**Table S1**) (100 µM each) in water was heated up to 95 °C for 5 min in a thermal cycler, and then gradually cooled down to 25 °C (0.02 °C/s). The resulting DNA (**70DNA_N^R**, **sgRNA_N^R**) (50 µM) was used as a template for *in vitro* transcription reactions.

2.3.2 General procedure (IV) (GP-IV) for 5'-end labelling via ligation reaction

The ligation reaction was performed in total volume of 30 µL in T4 RNA ligase buffer (1X) and DMSO (10%) with either natural or modified RNA (1 µg), rATP (1 mM), pCp-ATTO-488 (1 µM) and T4 RNA ligase 1000 U/ µL (1µL), according to supplier's protocol. The mixture was incubated at 16 °C in a thermal cycler for 16 h. The mixture was dissolved in 20 µL of water and purified by Monarch Kit (50 µg) following supplier's protocol. The samples were analyzed by gel electrophoresis on denaturing PAGE and visualized by fluorescence imaging using Cy2 channel.

2.3.3 *In vitro* transcription of 71-mer capped RNA

In vitro transcription reactions were performed using HiScribe T7 High Yield RNA synthesis Kit. Each reaction was carried out in a final volume of 10 µL, containing modified **N^{R}TP** (6 mM), two natural NTPs (6 mM), natural GTP (2 mM), DMSO (5%), Ribolock RNase inhibitor (1 U/ µL), dsDNA template (**70DNA_N^R**) (2 µM), T7 RNA polymerase (1 µL) and cap m^7GpppA$_m$pG (6 mM). For the negative control experiment, water was used instead of the solution of modified **N^{R}TP**, while the positive control contained the natural NTP of interest (6 mM). The mixture was incubated at 37°C for 3 h. The DNA template was then removed by treatment with DNase I (0.05 U/ µL) for 30 min at 37 °C. EDTA (50 mM) was added, and samples were heated at 65 °C for 10 min before purification with Monarch RNA Cleanup Kit columns following supplier's protocol. Samples were labeled following General Procedure IV (**GP-IV)** and analyzed by gel electrophoresis on 20% denaturing PAGE and visualized by fluorescence imaging (**Figure S3**). All samples were also characterized by LC-MS (**Table S4**).

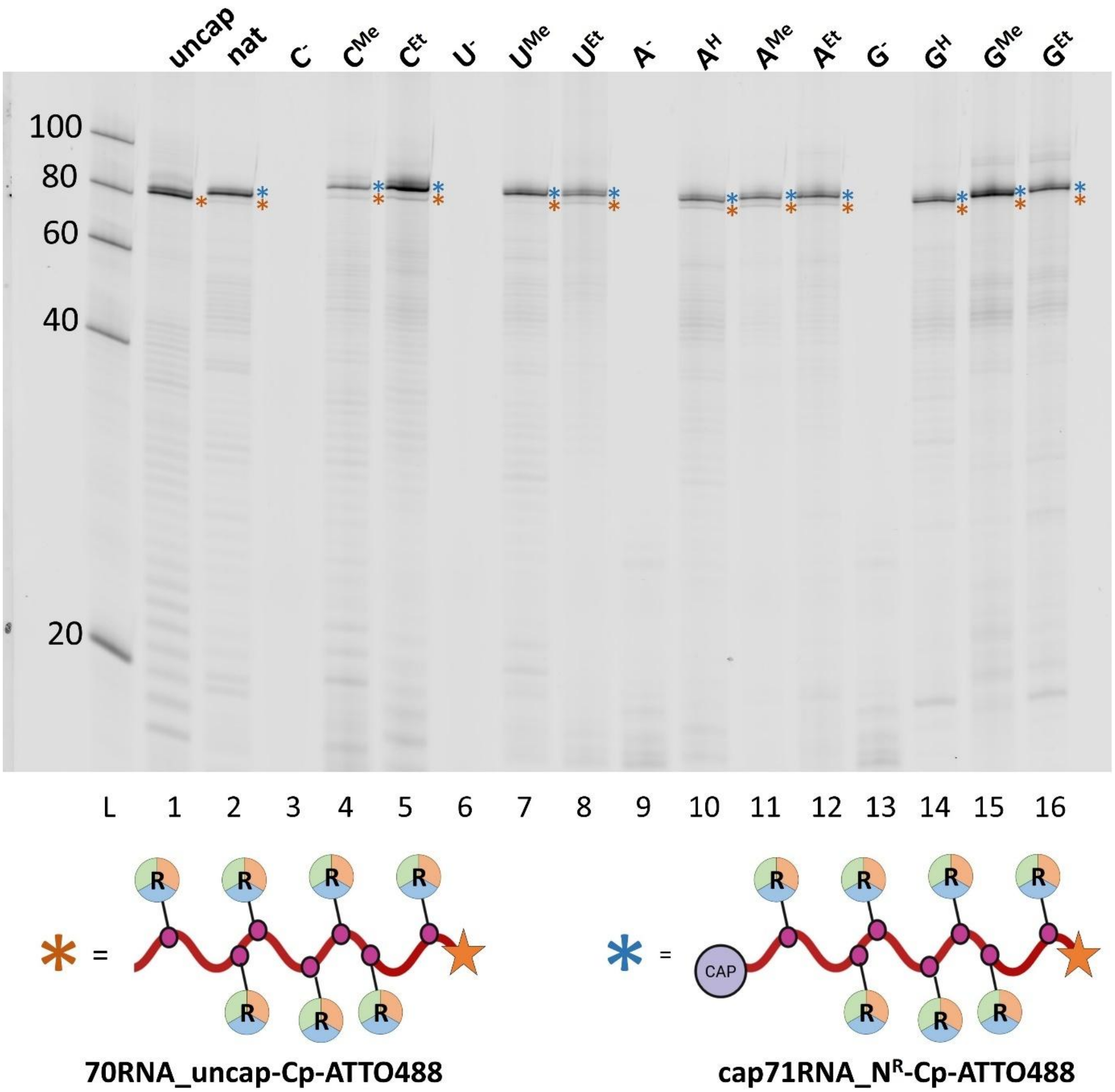

Figure S3: 20% dPAGE analysis of transcription reaction of 71-mer capped RNA. (L) DNA ladder (1) only natural NTPs, (2) natural NTPs with cap, (3,6,9,13) negative controls, three natural NTPs, without NTP or **N$^{\mathbf{R}}$TP** of interest; (4,5,7,8,10,11,12,14,15,16) three natural NTPs, with the modified N^{R}TP of interest.

Table S3 Capping efficiencies of 71-mer capped RNAs

Transcript	Capping efficiency (%)	Transcript	Capping efficiency (%
**cap71RNA_nat-ATTO488**	97.7	**cap71RNA_A^{Me}-ATTO488**	92.8
**cap71RNA_C^{Me}-ATTO488**	91.7	**cap71RNA_A^{Et}-ATTO488**	95.3
**cap71RNA_C^{Et}-ATTO488**	92.5	**cap71RNA_G^{H}-ATTO488**	99.4
**cap71RNA_U^{Me}-ATTO488**	98.7	**cap71RNA_G^{Me}-ATTO488**	100
**cap71RNA_U^{Et}-ATTO488**	95.8	**cap71RNA_G^{Et}-ATTO488**	100
**cap71RNA_A^{H}-ATTO488**	94.3		

Capping efficiencies were calculated using ImageJ from gel in **Figure S3**

Table S4 Molecular masses calculated and obtained of 71-mer capped RNAs

Transcript	Mw calc. [Da]	Mw found [Da]	Δ [Da]	Figure number
**70RNA_uncap**	22763	22763	0	S30
**cap71RNA_nat**	23378	23380	2	S31
**cap71RNA_C^{Me}**	23630	23632	2	S32
**cap71RNA_C^{Et}**	23882	23883	1	S33
**cap71RNA_U^{Me}**	23616	23619	3	S34
**cap71RNA_U^{Et}**	23854	23857	3	S35
**cap71RNA_A^{H}**	23366	23368	2	S36
**cap71RNA_A^{Me}**	23546	23540	6	S37
**cap71RNA_A^{Et}**	23714	23709	5	S38
**cap71RNA_G^{H}**	23356	23359	3	S39
**cap71RNA_G^{Me}**	23664	23667	3	S40
**cap71RNA_G^{Et}**	23972	23978	6	S41

2.3.4 Synthesis of modified 99-mer sgRNA oligonucleotides

2.3.4.1 Synthesis of DNA template

Sense and antisense oligonucleotides of the DNA template **sgDNA_N^R** (**Table S1**) were synthetized by solid-phase synthesis on an Applied Biosystems 394 automated DNA/RNA synthesizer. Synthesis of oligonucleotides were carried out on a 0.2 µmol scale using a standard phosphoramidite cycle. Cleavage from solid support and deprotection was done with a concentrated aqueous ammonia solution for 1h at RT and followed by heating at 55 ºC for 16 h. Obtained oligonucleotides were purified by HPLC (Ion exchange column; DNAPac PA 200 9x250mm, Thermoscientific). After HPLC the samples were desalted with AmiconUltra4 30K (Millipore). Then, annealing reaction was performed following General Porcedure III (**GP-III)**. The reaction was purified by HPLC (Ion-pairing reverse-phase; (Kinetex 5 µm EVO C18 100Å) and desalted with AmiconUltra4 30K (Millipore), using TRIS (10mM), EDTA (1mM), pH 8 as a buffer. Obtained dsDNA template was then amplified by PCR. The reaction mixture (50 µl) contained ds **sgDNA_N^R** (35ng), primFOR_sgRNA99 (2 µM), primREV_sgRNA99 (2 µM), set of four natural rNTP**s** (2mM), Q5 DNA polymerase (0.02 U/ µl) and the enzyme reaction buffer (5X). All reaction mixtures were under cycling protocol: 98°C for 30 sec, followed by 28 cycles at 98°C for 10 sec, 71°C for 30 sec, and 72°C for 2 min, followed by a final elongation step at 72°C for 2 min. Samples were analyzed by 2% agarose and visualized using fluorescence imaging (**Figure S4**).

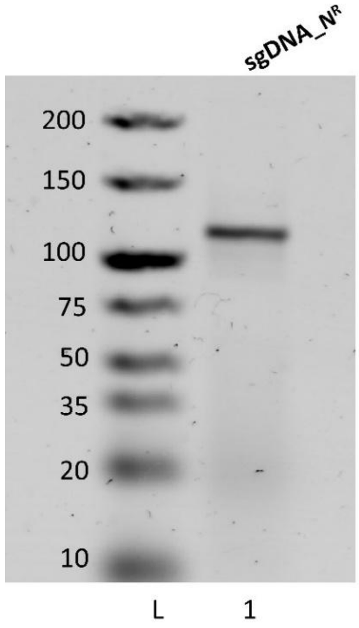

Figure S4: 2% Agarose gel analysis after PCR. (L) Ultra Low Range DNA ladder; (1) **sgDNA_N^R**.

2.3.4.2 *In vitro* transcription of sgRNA oligonucleotides

sgRNA targeting AAVS1 (sequence information in section 6 in SI) was synthesized by *in vitro* transcription using HiScribe T7 High Yield RNA synthesis Kit. Reactions were performed in the total volume of 10 µL containing three natural NTPs (7.5 mM), one modified **N^{X}TP** (7.5 mM), **sgRNA_N^{R}** (0.5 µg) and T7 RNA polymerase (0.75 µL). For the negative control experiment, water was used instead of the solution of modified **N^{R}TP**, while the positive control contained the natural NTP of interest (7.5 mM). The mixture was incubated at 37°C for 4 h. The DNA template was then removed by treatment with DNase I (0.05 U/ µL) for 30 min at 37 °C. Subsequently EDTA (50 mM) was added, and samples were heated at 65 °C for 10 min before purification with Monarch RNA Cleanup Kit columns following supplier's protocol and by HPLC (reverse-phase column; Biozen 2.6 µm oligo LC column 150 x 4.6 mm). Samples were labeled following **GP-IV** and analyzed by gel electrophoresis on 15% denaturing PAGE and visualized by fluorescence imaging (**Figure S5**). All samples were also characterized by LC-MS (**Table S5**).

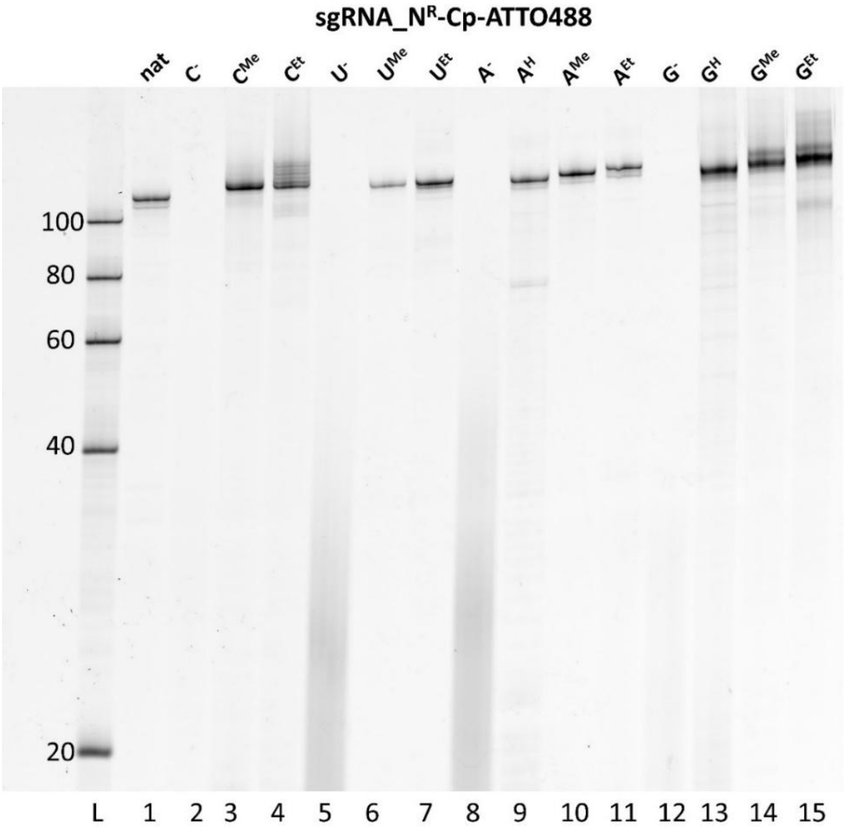

Figure S5: 15% dPAGE analysis of transcription reaction of sgRNA. (L) in-house prepared DNA ladder (1) only natural NTPs, (2,5,8,12) negative controls, three natural NTPs, without NTP or **N^{R}TP** of interest; (3,4,6,7,9,10,11,13,14,15) three natural NTPs, with the modified **N^{R}TP** of interest.

Table S5 Molecular masses calculated and obtained of sgRNAs

Transcript	Mw calc. [Da]	Mw found [Da]	Δ [Da]	Figure number
**sgRNA_nat**	32280	32307	27	S42
**sgRNA_C^{Me}**	32504	32550	46	S43
**sgRNA_C^{Et}**	32728	32773	45	S44
**sgRNA_U^{Me}**	32616	32660	44	S45
**sgRNA_U^{Et}**	32952	32987	35	S46
**sgRNA_A^{H}**	32249	32272	23	S47
**sgRNA_A^{Me}**	32714	32720	6	S48
**sgRNA_A^{Et}**	33148	33152	4	S49
**sgRNA_G^{H}**	32252	32253	1	S50
**sgRNA_G^{Me}**	32644	32690	46	S51
**sgRNA_G^{Et}**	33036	33070	34	S52

2.4 Synthesis of modified cap-mRNA

2.4.1.1 Linearization of plasmids

The linearization of vector phRL-SV40 was done with using bamHI-HF as restriction enzyme and for pGL4-CMV SalI-HF, following already described published protocol[10]. The samples were then analyzed by 1.3% agarose gel and visualized using fluorescence imaging (**Figure S6**). See the appendix section **6.2.1** and **6.2.2** for the vector's details.

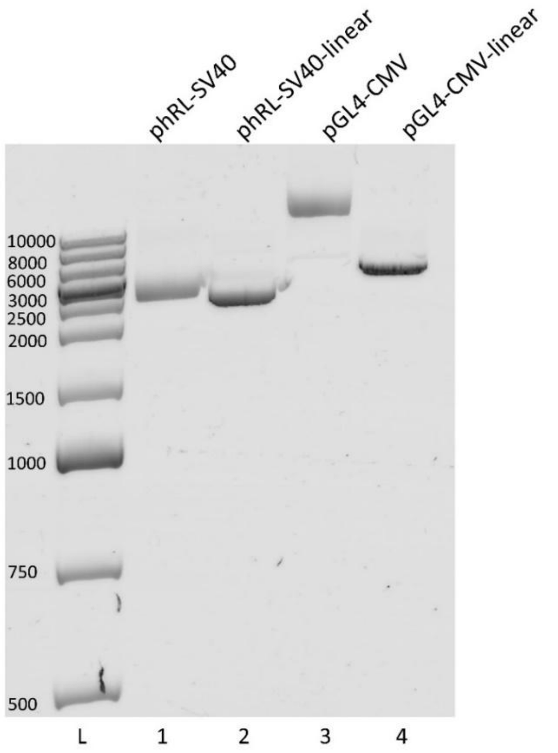

Figure S6: 1.3% agarose gel analysis of phRL-SV40 vector linearization. (L) GeneRuler 1 kb DNA ladder; (1,3) Circular form of the vectors; (2,4) linearized vectors.

2.4.1.2 *In vitro* transcription of mRNA

In vitro transcription reactions were performed using HiScribe T7 High Yield RNA synthesis Kit in a final volume of 10 µL containing modified **N^R^TP** (10 mM), two natural NTPs (10 mM), natural GTP (2 mM), DMSO (5%), Ribolock RNase inhibitor (1 U/ µL), pDNA template **phRL-SV40-linear** or **pGL4-CMV-linear** (250 ng) , T7 RNA polymerase (1 µL) and cap m7GpppA$_m$pG (8 mM). For the negative control experiment, water was used instead of the solution of modified **N^{R}TP**, while the positive control contained the natural NTP of interest (10 mM). The mixture was incubated at 37°C for 4 h. The DNA template was then removed by treatment with DNase I (0.05 U/ µL) for 30 min at 37 °C. EDTA (50 mM) was added, and samples were heated at 65 °C for 10 min before purification with Monarch RNA Cleanup Kit columns following supplier's protocol. Samples were labelled following **GP-IV** and analyzed by gel electrophoresis on 7.5% denaturing PAGE and visualized by fluorescence imaging (**Figure S7**).

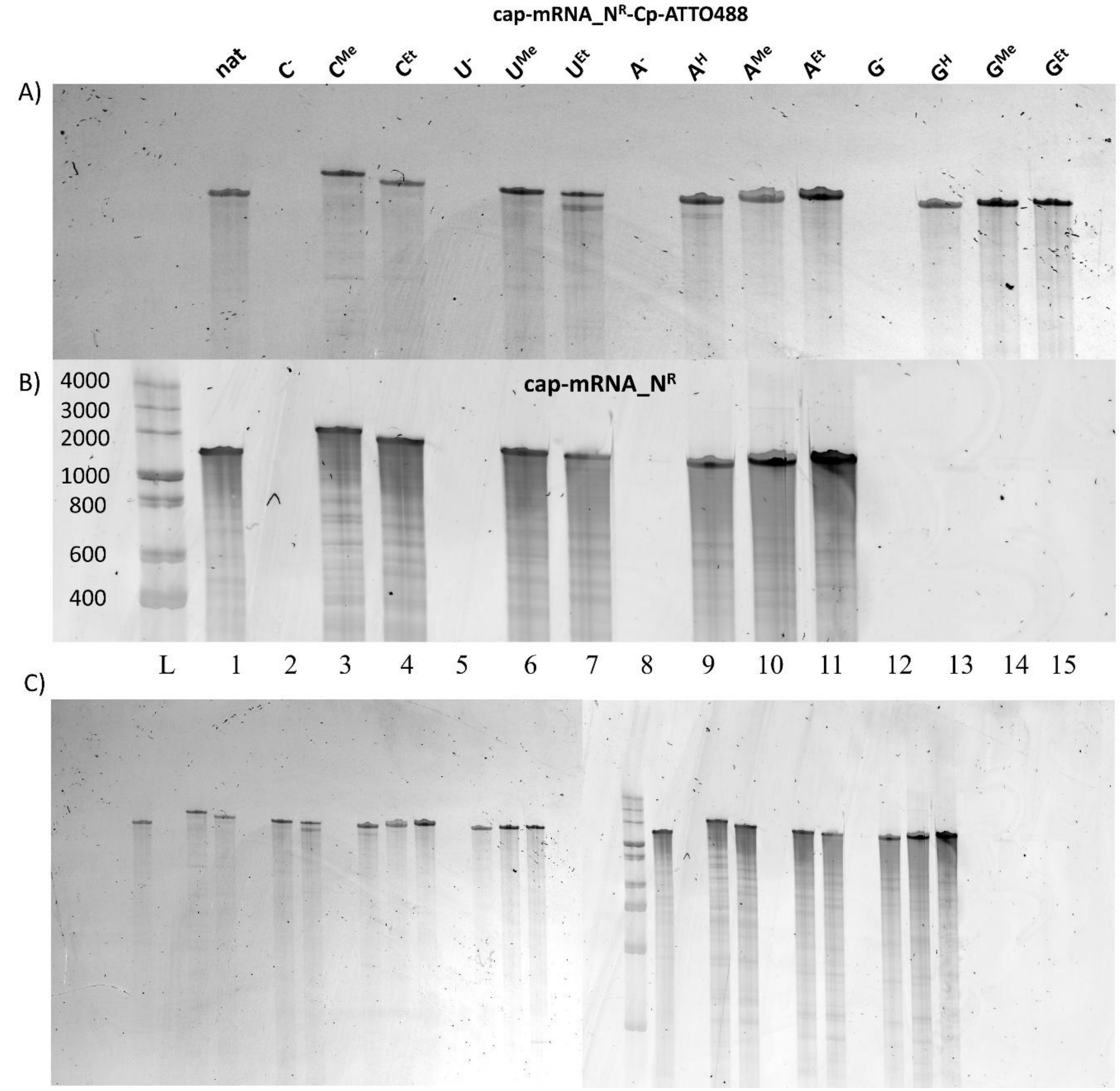

Figure S7: 7.5% dPAGE analysis of transcription reaction of mRNA visualized using (A) Cy2 channel or (B) Cy3 channel after staining with SYBR gold. (L) RNA ladder, mix of RiboRuler High and Low Range RNA ladder (1) only natural NTPs, (2,5,8,12) negative controls, three natural NTPs, without NTP or **N^{R}TP** of interest; (3,4,6,7,9,10,11,13,14,15) three natural NTPs, with the modified **N^{R}TP** of interest. (C) uncropped gels of A and B.

2.5 CRISPR-Cas9 *in vitro* DNA cleavage experiments

2.5.1 Isolation of genomic DNA and PCR amplification of AAVS1 on-target

Genomic DNA from HEK-293 cells was isolated using Invitrogen Genomic DNA Mini kit following the Mammalian Cells and Blood Lysate protocol and purification protocol. AAVS1, target DNA for CRISPR experiments, was amplified from HEK-293 DNA using PCR. In a 100 µL reaction, Q5 1X Master Mix (50 µL), 0.5 µM AAVS1_FWR primer (5 µL), 0.5 µM AAVS1_REV primer (5 µL), 3.17 ng/µL template (10 µL) and water (30 µL) were mixed and the reaction was carried out under the following conditions: 98 ºC, 30 sec; 35 cycles of 98 ºC,10 sec, 69 ºC 30 sec, 72 ºC 25 sec and for the final extension 72 ºC,1 20 sec. The reaction was purified using Monarch PCR & DNA Cleanup kit 5µg.

2.5.2 Automated on-chip electrophoresis

Automated on-chip electrophoresis of nucleic acids was carried out on the Agilent 2100 Bioanalyzer instrument. For instrument control, data acquisition and data processing, 2100 Expert B.02.11 (Agilent Technologies Inc.) software was used. Electrophoretic separations of dsDNA fragments of AAVS1 after *in vitro* CRISPR-Cas9 cleavage were carried out with the Agilent DNA 1000 kit using the manufacturer's protocol.

2.5.3 CRISPR-Cas9 *in vitro* DNA cleavage with modified sgRNAs

The reactions were carried out in PCR tubes placed in a thermocycler for precise heating control. A Cas9 master mix (for 10 reaction) was prepared by mixing 39 µL of water with 10 µL of buffer 3.1 and 1 µL of 20 mM solution of spCas9. Then, 5 µL master mix (2 pmol) was added in every PCR tube and 2.5 µL of 1 µM **sgRNA_N$^{\mathbf{R}}$** solution (2.5 pmol). The mixture was incubated for 10 min at 37 ºC to form Cas9-sgRNA complex. Next, 2.5 µL of 0.1 µM of AAVS1 target DNA solution was added and the reaction mixture was incubated for 30 min at 37 ºC. The reaction was stopped by increasing the temperature to 65 ºC for 5 min. Subsequently, 1 µL RNAse A/T1 mix was added, and the reaction mixture was incubated for 30 min at 37 ºC, followed by addition of 1 µL of proteinase K and incubated for 15 min at 65 ºC. For quantification of DNA fragments, on-chip electrophoresis was used. Reported cleavage efficiencies were calculated from data collected during three independent experiments, using the equation $\eta = \frac{A_{p1}+A_{p2}}{A_{p1}+A_{p2}+A_s} * 100$, where A_{p1} and A_{p2} are time-corrected areas of cleaved DNA fragments and A_s is the time-corrected area of the full-length DNA fragment (not cleaved) (**Figure S8**). Cleavage efficiencies were quantified from three independent

experiments. Data are expressed as mean ± SEM. Replicates showed low variability, and observed differences were consistent across all experiments (**Table S6**).

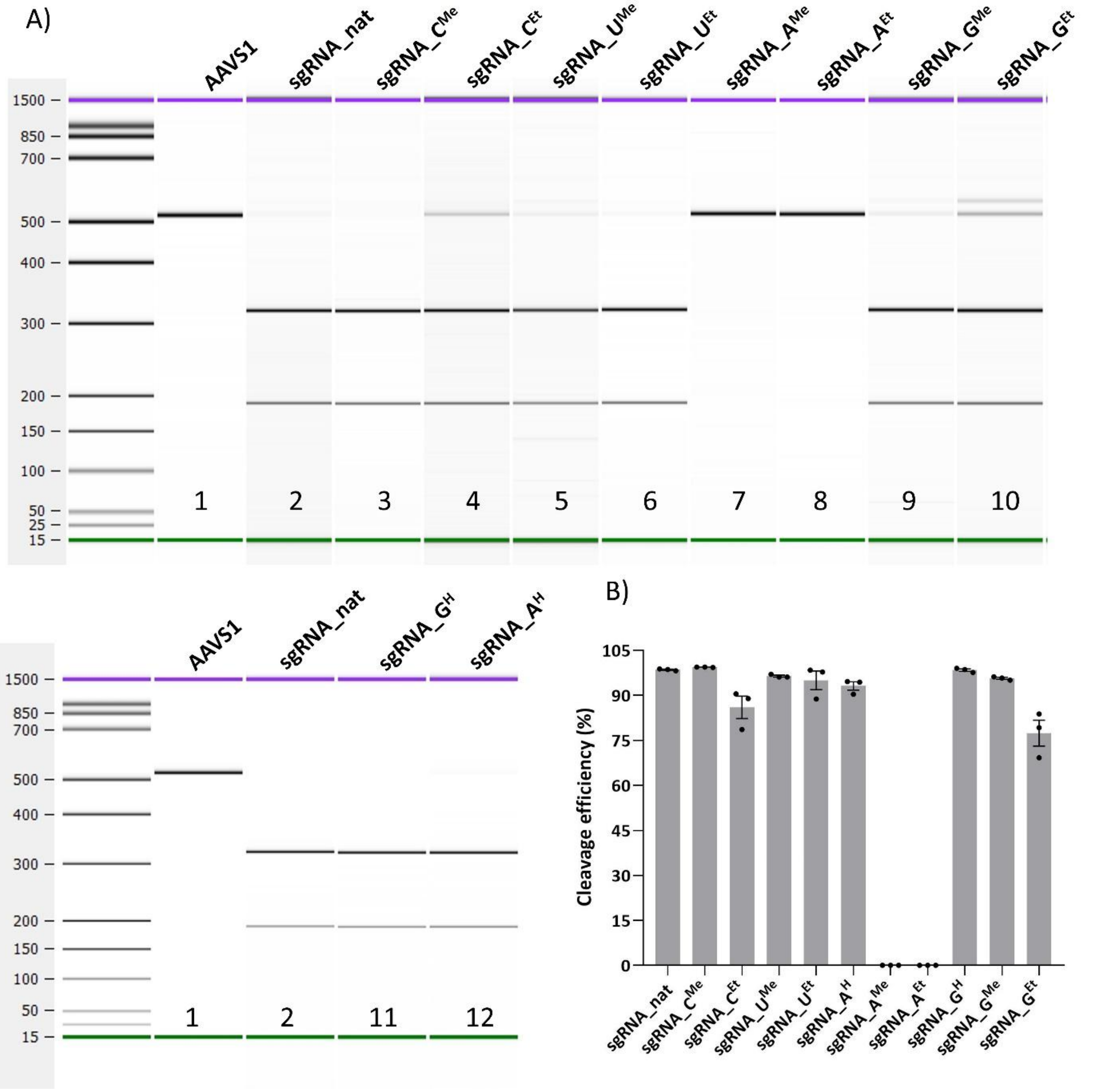

Figure S8: (A) Electrophoresis on-chip analysis of CRISPR-Cas9 cleavage of the AAVS1 target using natural and chemically modified **sgRNA_N^{R}**. (1) uncleaved full-length AAVS1 DNA; (2) cleavage with natural sgRNA (**sgRNA_nat**); (3-12) cleavage with various modified sgRNAs (**sgRNA_N^{R}**). (B) Quantification of AAVS1 cleavage efficiency (%) by Cas9 in the presence of natural (**sgRNA_nat**) or modified sgRNAs (**sgRNA_N^{R}**), based on fragment analysis. Data represents mean ± SEM from three independent experiments (n = 3).

Table S6 Mean of cleavage efficiency and standard deviation (SD) from CRISPR-Cas9 cleavage experiment of different **sgRNA_N^{R}** for n = 3

	Mean (Cleavage efficiency)	SD
**sgRNA_nat**	98.5	0.26
**sgRNA_C^{Me}**	99.4	0.05
**sgRNA_C^{Et}**	86	5.27
**sgRNA_U^{Me}**	96.4	0.42
**sgRNA_U^{Et}**	95	4.39
**sgRNA_A^{H}**	93.1	1.9
**sgRNA_A^{Me}**	0	0
**sgRNA_A^{Et}**	0	0
**sgRNA_G^{H}**	98.4	0.6
**sgRNA_G^{Me}**	95.7	0.45
**sgRNA_G^{Et}**	77.4	6.09

2.6 Stability of the sgRNAs in human serum

2.6.1 Stability experiment in human serum with Cas9

The stability of all labelled sgRNAs (prepared as described in section **2.3.4.2** in SI) was assessed in PCR tubes (25 µL final volume). First, **sgRNA_N^{R}-Cp-ATTO488** (100 nM) was mixed with Cas9 nuclease (0.5 µM) and incubated at 37 ºC for 10 min. Human serum (10%) and PBS (1X) were then added and the reaction was further incubated at 37 ºC.

Samples were collected at 0 min, 15 min, 30 min, 1 h and 2 h. At each time point, 5 µL of the reaction mixture was diluted in 5 µL of water and immediately flash-frozen in liquid nitrogen. Next, 1 µL of proteinase K was added, following the addition of 10 µL of loading dye and the reaction was incubated for 15 min at 65 ºC. The samples were analyzed by gel electrophoresis on 15% denaturing PAGE and visualized by fluorescence imaging (**Figure S9**). Remaining sgRNA over the time was quantified (**Figure S10**), and the half-lives were calculated by fitting one phase decay model in GraphPad (**Figure S11**). Each analysis was done in triplicates (**Table S7**).

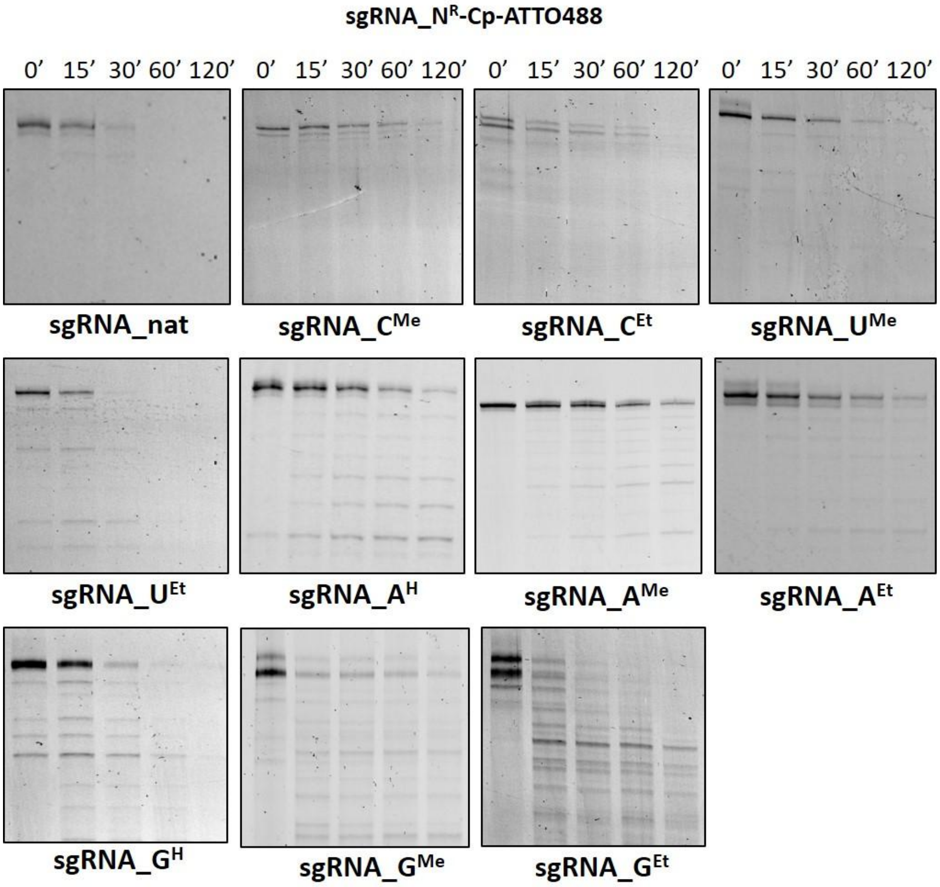

Figure S9: 15% dPAGE analysis of stability experiment of natural and chemically modified **sgRNA_N^R-Cp-ATTO488** in 10% human serum at 37 °C. Representative gels from three independent experiments are shown.

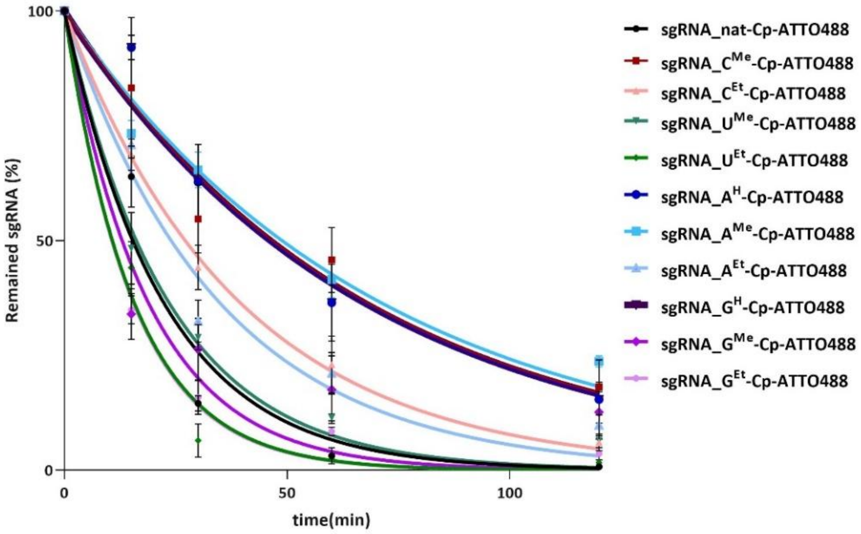

Figure S10: Quantification of **sgRNA_nat-Cp-ATTO488** and **sgRNA_N^R-Cp-ATTO488** remaining over time following incubation in 10% human serum at 37 °C. Fluorescent band intensities from denaturing PAGE (**Figure S9**) were quantified using ImageJ and normalized to the signal at time 0. Data represents mean ± SEM from three independent experiments (n = 3).

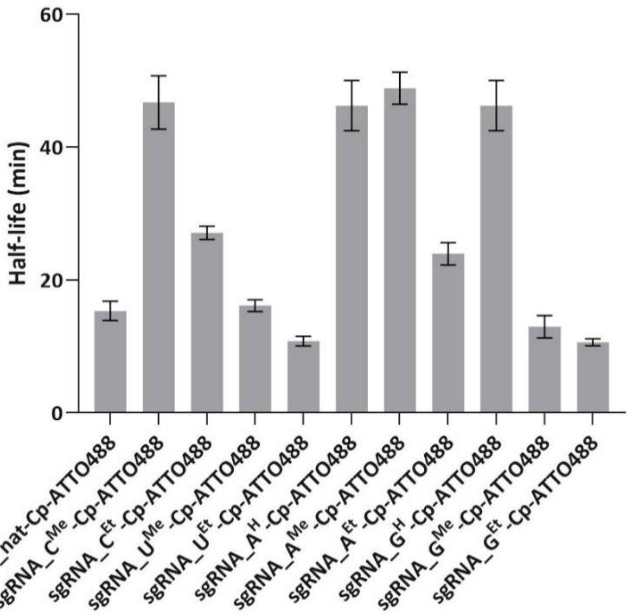

Figure S11: Stability analysis of **sgRNA_nat-Cp-ATTO488** and **sgRNA_N^R-Cp-ATTO488** in human serum. Normalized degradation data from three independent experiments (**Figure S10**) were averaged and fitted with a one-phase exponential decay model in GraphPad Prism. The reported half-life was calculated from the average degradation curve. Curve fitting yielded R^2 values ranging from 0.8863 to 0.9891 (mean R^2 = 0.957), indicating a good fit to the decay model. Data are shown as mean ± SEM (n = 3).

Table S7 Mean of normalized values of remaining RNA (%) and standard deviation (SD) from stability in human serum experiment of different **sgRNA_N^{R}-Cp-ATTO488** for n = 3

	Time (min)	Mean	SD		Time (min)	Mean	SD
**sgRNA_nat-Cp-ATTO488**	0	100.00	0.00	**sgRNA_C^{Me}-Cp-ATTO488**	0	100.00	0.00
	15	63.93	5.43		15	83.26	12.48
	30	14.56	1.37		30	54.71	6.06
	60	3.12	1.45		60	45.77	5.78
	120	0.74	0.22		120	18.11	0.86
**sgRNA_C^{Et}-Cp-ATTO488**	0	100.00	0.00	**sgRNA_U^{Me}-Cp-ATTO488**	0	100.00	0.00
	15	52.79	3.42		15	48.31	6.39
	30	35.84	2.97		30	28.93	2.25
	60	18.79	2.61		60	11.47	0.58
	120	10.64	1.41		120	6.46	0.21
**sgRNA_U^{Et}-Cp-ATTO488**	0	100.00	0.00	**sgRNA_A^{H}-Cp-ATTO488**	0	100.00	0.00
	15	44.13	4.64		15	92.03	2.20
	30	6.44	2.95		30	62.76	6.69
	60	2.39	0.45		60	36.47	6.80
	120	1.65	0.58		120	15.38	7.04
**sgRNA_A^{Me}-Cp-ATTO488**	0	100.00	0.00	**sgRNA_A^{Et}-Cp-ATTO488**	0	100.00	0.00
	15	73.31	2.32		15	70.85	2.02
	30	65.40	3.16		30	32.45	3.74
	60	41.52	3.23		60	21.08	3.72
	120	23.70	1.01		120	9.76	1.85
**sgRNA_G^{H}-Cp-ATTO488**	0	100.00	0.00	**sgRNA_G^{Me}-Cp-ATTO488**	0	100.00	0.00
	15	70.14	5.88		15	33.98	4.50
	30	13.55	2.27		30	26.35	5.47
	60	5.59	1.40		60	17.54	6.00
	120	2.47	0.91		120	12.65	1.96
**sgRNA_G^{Et}-Cp-ATTO488**	0	100.00	0.00				
	15	34.93	2.48				
	30	15.84	2.99				
	60	8.45	0.75				
	120	3.56	1.12				

2.6.2 Stability experiment in human serum without Cas9

The stability of labelled **sgRNA_A^{H}** and **sgRNA_A^{Me}** (prepared as described in section **2.3.4.2**) was assessed in PCR tubes (25 µL final volume). First, **sgRNA_N^{R}-Cp-ATTO488** (100 nM) was mixed with human serum (10%) and PBS (1X) and the reaction was further incubated at 37 ºC. Samples were collected at 0 min, 15 min, 30 min, 1 h and 2 h. At each time point, 5 µL of the reaction mixture was diluted in 5 µL of water and immediately flash-frozen in liquid nitrogen. Next, 10 µL of loading dye was added and the reaction was incubated for 5 min at 65 ºC. The samples were analyzed by gel electrophoresis on 15% denaturing PAGE and visualized by fluorescence imaging (**Figure S12**).

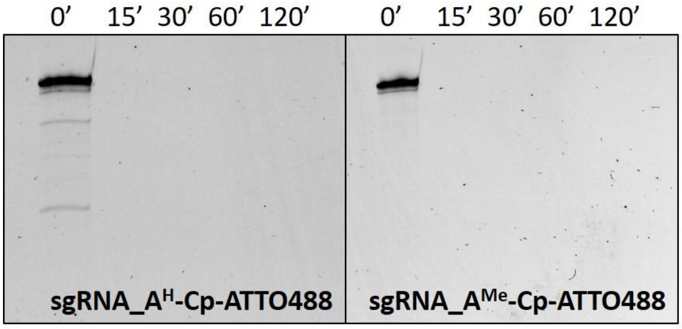

Figure S12: 15% dPAGE analysis of stability experiment of chemically modified **sgRNA_A^{H}-Cp-ATTO488** and **sgRNA_A^{Me}-Cp-ATTO488** in 10% human serum at 37 °C.

2.7 *In vitro* translation studies

Translation efficiency experiments measured by luminescence signal were carried out in Rabbit Reticulocyte Lysate System (RRLS). The reaction mixture (RRL, 7 µL) was supplemented with Complete Amino Acid mixture 1 mM (0.5 µL), Ribolock RNase inhibitor 40 U/ µL (0.5 µL) and 50 ng of **cap-mRNA_N^{R}**. Reaction was incubated at 30 ºC for 1.5 h and then stopped by freezing the samples at –80 ºC. For luminescence detection, 2.5 µL of reaction was diluted with 2.5 µL of water in a 384-well plate. *Renilla* luciferase activity was measured using Spark® Multimode Microplate Reader (Tecan), following the injection of 40 µL of *Renilla* Luciferase buffer (**Figure S13**). The data shown represents the results of two biological replicates, each performed in technical triplicate. Raw luminescence values were normalized to the signal obtained from the natural mRNA control (**cap-mRNA_nat**), which was set as 100%. The relative translation efficiency of modified **cap-mRNA_N^{R}** constructs was expressed as a percentage (**Table S8**).

Table S8 Mean of normalized values (%), standard deviation (SD) and standard error of the mean (SEM) from luminescence measurement of *Renilla* luciferase *in vitro* for n = 3

	Mean	SD	SEM
**cap-mRNA_nat**	100.00	6.22	2.20
**cap-mRNA_C^{Me}**	148.00	15.22	5.38
**cap-mRNA_C^{Et}**	103.28	15.34	5.42
**cap-mRNA_U^{Me}**	185.54	27.95	9.88
**cap-mRNA_U^{Et}**	29.39	10.28	3.63
**cap-mRNA_A^{H}**	0.69	0.27	0.10
**cap-mRNA_A^{Me}**	0.01	0.00	0.00
**cap-mRNA_A^{Et}**	0.00	0.00	0.00
**cap-mRNA_G^{H}**	61.79	9.44	3.34
**cap-mRNA_G^{Me}**	6.44	1.08	0.38
**cap-mRNA_G^{Et}**	2.78	0.39	0.14

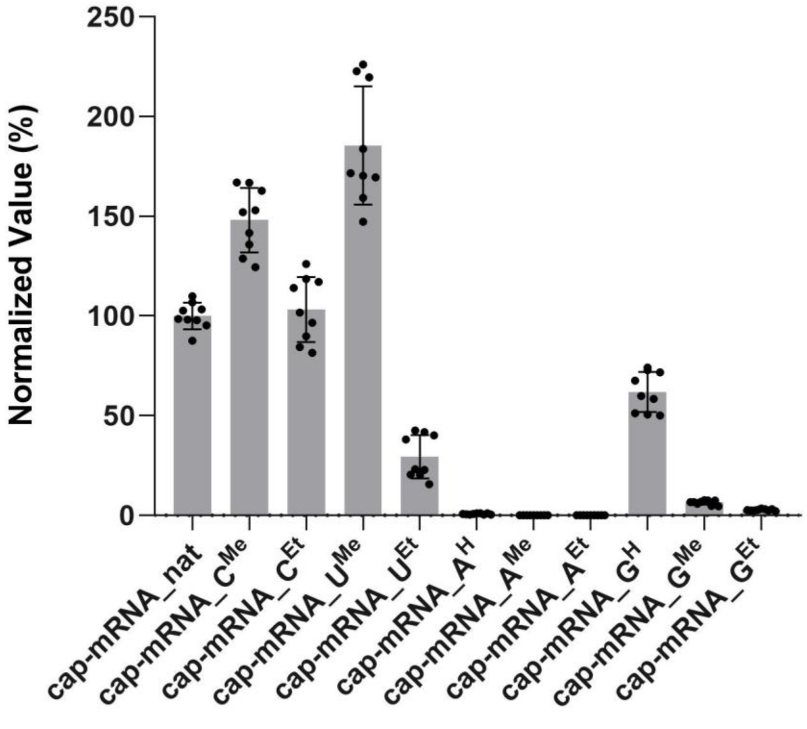

Figure S13: *In vitro* translation analysis of natural mRNA (**cap-mRNA_nat**) and modified (**cap-mRNA_N^{R}**) encoding *Renilla* luciferase. Translation efficiency is expressed as luminescence signal normalized to natural mRNA (**cap-mRNA_nat**) set as 100%. Data represent mean ± SEM from three biological replicates (n = 3), each performed in technical triplicate. GraphPad Prism was used for data analysis and visualization.

2.7.1 10% SDS PAGE

In vitro translation was performed following the same procedure described in Section **2.6**, with the addition of 1 µL of the FluroTech Green Lys labeling system to enable visualization of the synthesized luciferase protein. Following incubation, samples were resolved by 10% SDS-PAGE, and fluorescently labeled proteins were visualized using a fluorescence imaging system (**Figure S14**).

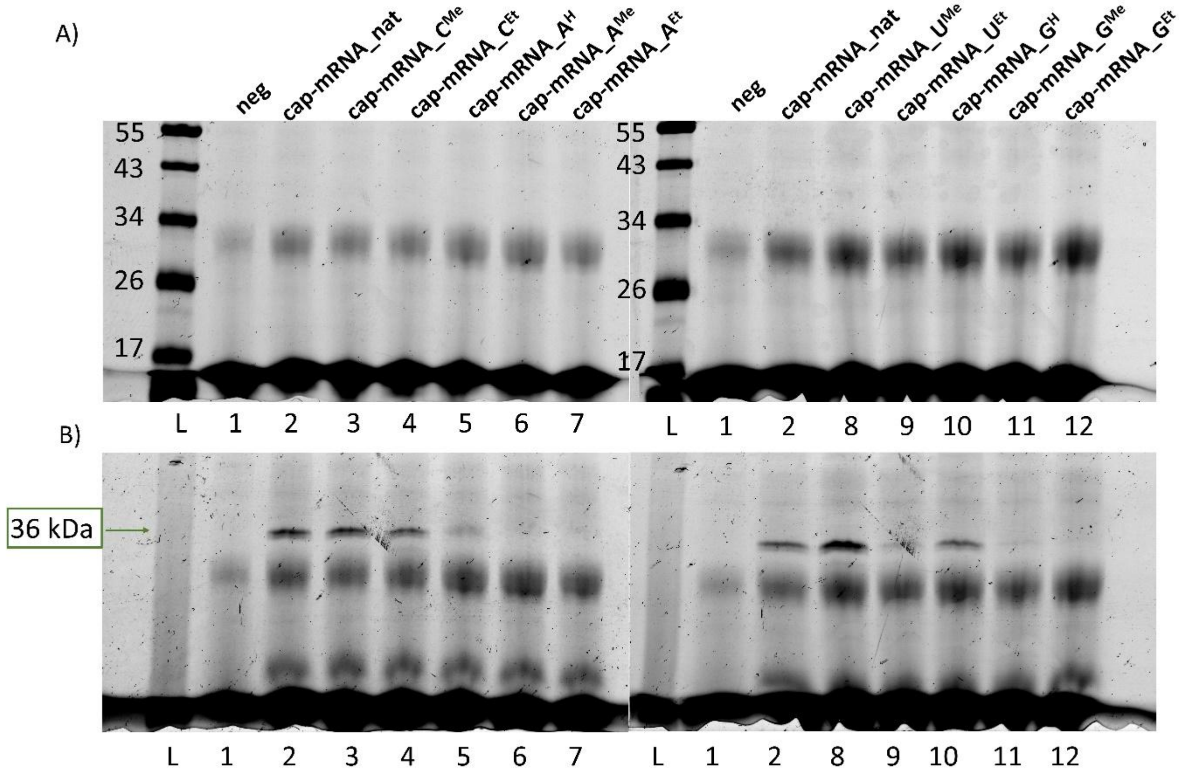

Figure S14: 10% SDS PAGE analysis of *in vitro* translation experiment with modified **cap-mRNA_N^{R}** using FluroTech Green Lys labelling. (A) Scan using Cy5 channel; (B) Scan using Cy2 channel. (L) Color Prestained Protein Standar, Broad Range (10-250 kDa ladder; (1) Negative control, without mRNA; (2) Positive control, natural mRNA (**cap-mRNA_nat**); (3-12) *Renilla* luciferase protein level from translation with different modified **cap-mRNA_N^{R}**.

2.8 *In cellulo* translation studies

2.8.1 Transfection of *Renilla* luciferase mRNA

The **cap-mRNA_nat** and **cap-mRNA_N^{R}** were transfected to HeLa S3 cells using Lipofectamine MessengerMax transfection reagent according to manufacturer's instructions: One day before the cells were transfected at 50% confluency in a 24-well plate. The transfection mixture per one well contained 70 ng of mRNA encoding *Renilla* luciferase. Three hours after

transfection, the transfection reagent was washed away by substituting the medium for fresh complete medium. At defined time points (3 h, 6 h, 12 h, 24 h, and 36 h post-transfection), cells were washed with 1X PBS and stored at –80 °C until further processing. For lysis, the cells were removed refrozen and lysed with 100 µL PPBT lysis buffer (0.2% v/v Triton X-100, 100 mM potassium phosphate buffer, pH 7.8) per well for 10 min at room temperature with soft shaking. For luminescence measurement, 5 µL of lysate was transferred into a 384-well plate. Using TECAN Spark® Multimode Microplate Reader, 40 µL of *Renilla* luciferase buffer was injected and luminescence was measured (**Figure S15**). The data shown represents the results of three biological replicates, each performed in technical triplicate. Raw luminescence values were normalized to the signal obtained from the natural mRNA control (**cap-mRNA_nat**) at 3h, which was set as 100%. The relative translation efficiency of modified **cap-mRNA_N^{R}** constructs was expressed as a percentage (**Table S9**).

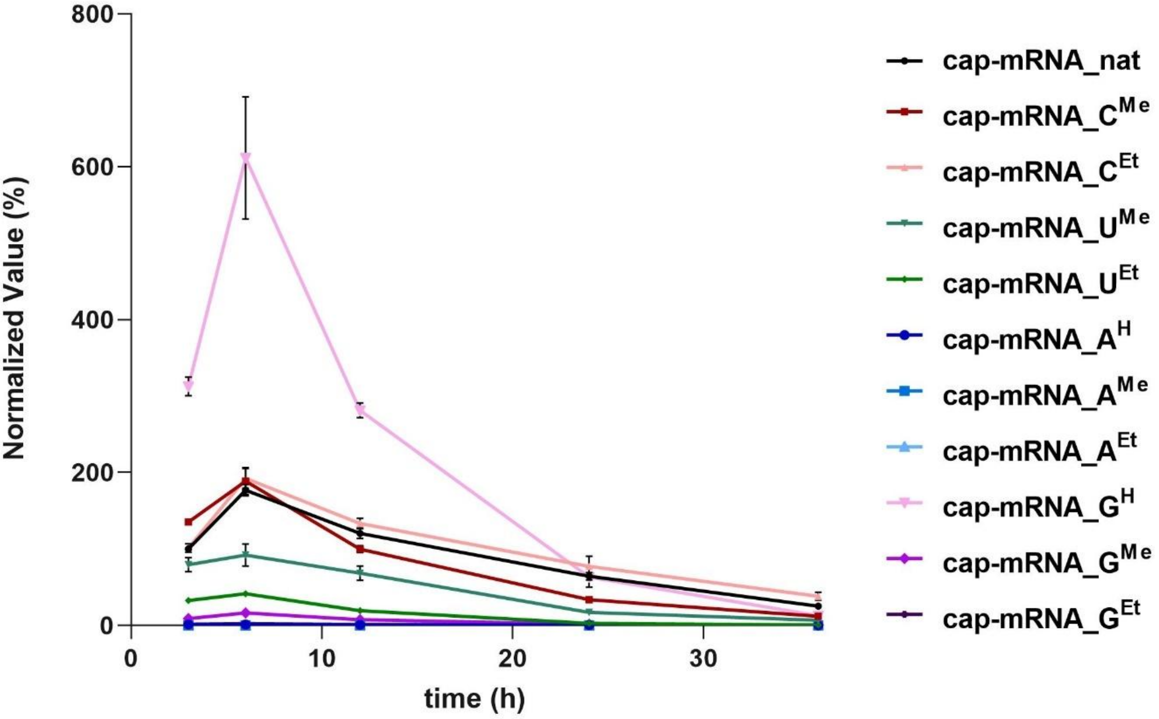

Figure S15: Time-course *Renilla* luciferase activity from cells transfected with natural (**cap-mRNA_nat**) and chemically modified (**cap-mRNA_N^{R}**) mRNAs, using Lipofectamine MessengerMAX and collected at 3, 6, 12, 24, and 36 h post-transfection. Data are normalized to the luminescence signal of **cap-mRNA_nat** at 3 h (set as 100%) and represent mean ± SD from three biological replicates (n = 3), each performed in technical triplicate.

Table S9 Mean of normalized values (%), standard deviation (SD) and standard error of the mean (SEM) from luminescence measurement of *Renilla* luciferase *in cellulo* for n = 3

	3h			6h			12h		
	Mean	SD	SEM	Mean	SD	SEM	Mean	SD	SEM
**cap-mRNA_nat**	100.00	0.85	0.30	177.07	7.08	2.50	120.43	6.15	2.18
**cap-mRNA_C^{Me}**	135.31	2.25	0.80	188.77	16.76	5.93	99.80	4.33	1.53
**cap-mRNA_C^{Et}**	101.14	5.28	1.87	192.30	12.36	4.37	133.33	6.36	2.25
**cap-mRNA_U^{Me}**	79.61	8.88	3.14	92.05	13.42	4.75	68.42	8.60	3.04
**cap-mRNA_U^{Et}**	32.55	4.04	1.43	41.68	1.93	0.68	19.55	2.32	0.82
**cap-mRNA_A^{H}**	1.11	0.11	0.04	1.38	0.10	0.04	0.82	0.09	0.03
**cap-mRNA_A^{Me}**	0.26	0.04	0.01	0.36	0.07	0.02	0.33	0.04	0.01
**cap-mRNA_A^{Et}**	0.71	0.15	0.05	0.32	0.06	0.02	0.52	0.09	0.03
**cap-mRNA_G^{H}**	312.63	11.48	4.06	611.64	75.37	26.65	281.36	8.98	3.17
**cap-mRNA_G^{Me}**	9.21	0.35	0.13	16.33	1.68	0.59	7.75	0.45	0.16
**cap-mRNA_G^{Et}**	1.74	0.11	0.04	2.50	0.38	0.13	1.29	0.11	0.04

	24h			36h		
	Mean	SD	SEM	Mean	SD	SEM
**cap-mRNA_nat**	64.22	4.64	1.64	25.42	2.04	0.72
**cap-mRNA_C^{Me}**	33.87	3.71	1.31	12.07	0.93	0.33
**cap-mRNA_C^{Et}**	77.17	12.49	4.42	38.14	4.76	1.68
**cap-mRNA_U^{Me}**	17.24	2.06	0.73	6.79	0.52	0.19
**cap-mRNA_U^{Et}**	2.66	0.15	0.05	0.63	0.07	0.02
**cap-mRNA_A^{H}**	0.46	0.04	0.01	0.30	0.03	0.01
**cap-mRNA_A^{Me}**	0.33	0.04	0.01	0.25	0.04	0.01
**cap-mRNA_A^{Et}**	0.29	0.03	0.01	0.53	0.12	0.04
**cap-mRNA_G^{H}**	63.26	12.72	4.50	12.86	1.99	0.70
**cap-mRNA_G^{Me}**	1.59	0.25	0.09	0.53	0.08	0.03
**cap-mRNA_G^{Et}**	0.41	0.06	0.02	0.34	0.05	0.02

2.8.2 RNA isolation and quantification by digital droplet PCR (ddPCR)

Total RNA was isolated from 70 µL of cell lysate, mixed with 280 µL of RNA lysis buffer, using the Quick-RNA Microprep Kit (Zymo Research), following the manufacturer's protocol and including on-column DNase I treatment. RNA was eluted in 20 µL of nuclease-free water. For quantification of transfected mRNA, reverse transcription was performed from 200 ng total RNA using Quantitect Reverse Transcription Kit (Qiagen) following the manufacture's protocol. The resulting cDNA was analyzed by digital droplet PCR on the QX200 Droplet Digital PCR System (Bio-Rad) with primer specific for *Renilla* luciferase **hRL_Fwd** and **hRL_Rev**. Droplets were generated using the Automated Droplet Generator and amplified using the EvaGreen Supermix (Bio-Rad) following the manufacturer's protocol. Droplet fluorescence was measured with the QX200 Droplet Reader, and absolute transcript copy numbers were calculated using QuantaSoft analysis software. All ddPCR-measured mRNA levels were normalized to the value of **cap-mRNA_nat** at 3 hours, which was set to 100% (**Table S10**). The data shown represents the results of three biological replicates (n = 3), each performed in technical triplicate (**Figure S16**).

Table S10 Mean of normalized values (%) and standard deviation (SD) from ddPCR measurement of cDNA encoding for *Renilla* luciferase for n = 3

	3h			6h		
	Mean	SD	SEM	Mean	SD	SEM
**cDNA_nat**	100.00	2.29	0.76	34.35	46.42	15.47
**cDNA_C^{Me}**	95.57	19.71	6.57	40.62	39.23	13.08
**cDNA_U^{Me}**	80.35	15.22	5.07	33.55	33.35	11.12
**cDNA_A^{H}**	69.62	19.41	6.47	31.83	27.23	9.08
**cDNA_A^{Me}**	39.11	15.69	5.23	20.01	14.17	4.72
**cDNA_G^{H}**	94.28	16.48	5.49	38.75	39.52	13.17
**cDNA_G^{Me}**	21.54	13.84	4.61	13.33	6.92	2.31
**cDNA_G^{Et}**	14.61	7.56	2.52	8.23	4.96	1.65

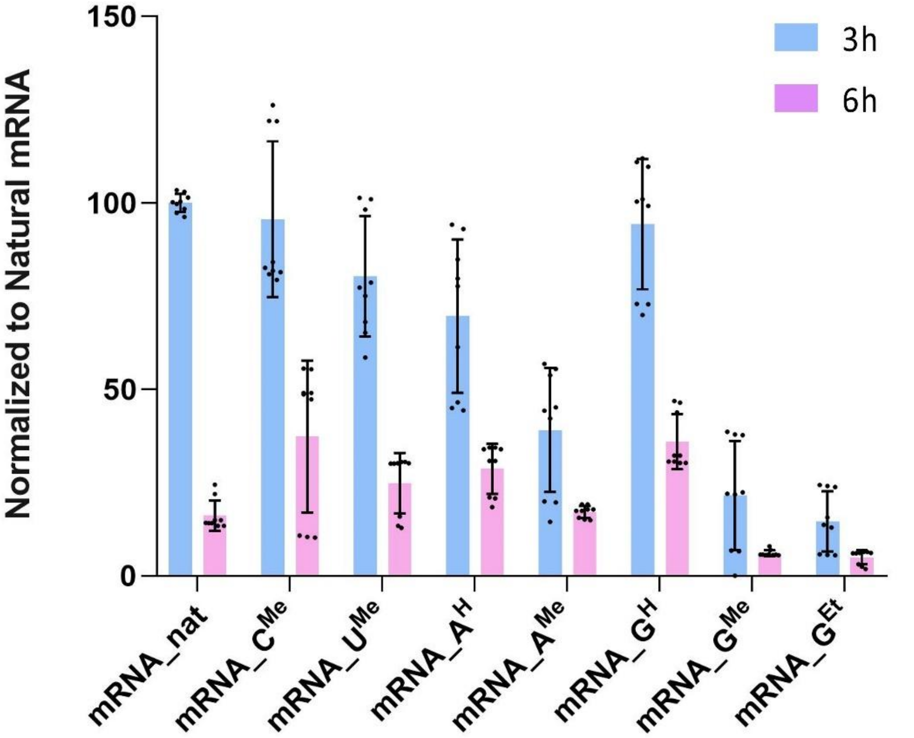

Figure S16: RNA was isolated, reverse transcribed, and the corresponding cDNA was quantified using EvaGreen-based digital droplet PCR (ddPCR). As cDNA levels reflect original mRNA abundance, absolute transcript copies/ng RNA are used to represent the levels of the transfected mRNA. Values are normalized to **cDNA_nat** at 3 h (set as 100%) and represent mean ± SD from three biological replicates (n = 3), each performed in technical triplicate.

2.8.3 Co-transfection of *Renilla* luciferase mRNA with *Firefly* luciferase

The **cap-mRNA_nat** and **cap-mRNA_N^{R}** were co-transfected with Firefly luciferase (**mRNA_natFF**) to HeLa S3 cells using Lipofectamine MessengerMax transfection reagent according to manufacturer's instructions: One day before the cells were transfected at 50% confluency in a 24-well plate. The transfection mixture per one well contained 70 ng of **cap-mRNA_N^{R}** and 50 ng of **mRNA_natFF**. Three hours after transfection, the transfection reagent was washed away by substituting the medium for fresh complete medium. At defined time points (3 h, 6 h, 12 h, 24 h, and 36 h post-transfection), cells were washed with 1X PBS and stored at –80 °C until further processing. For lysis, the cells were removed refrozen and lysed with 100 µL PPBT lysis buffer (0.2% v/v Triton X-100, 100 mM potassium phosphate buffer, pH 7.8) per well for 10 min at room temperature with soft shaking. For luminescence measurement, 5 µL of lysate was transferred into a 384-well plate. Using TECAN Spark® Multimode Microplate Reader, 40 µL of *Firefly* luciferase substrate was injected and

luminescence was measured, followed by addition of 40 µL of *Renilla* luciferase substrate that stops the FF luciferase activity, and allows the assessment of *Renilla* luciferase expression (**Figure S17**). The data shown represents the results of three biological replicates, each performed in technical triplicate. Raw luminescence values were normalized to the signal obtained from the natural mRNA control encoding Firefly luciferase **mRNA_natFF** and then normalized to **cap-mRNA_nat** at 3h, which was set as 100%. The relative translation efficiency of modified **cap-mRNA_N^{R}** constructs was expressed as a percentage (**Table S11**).

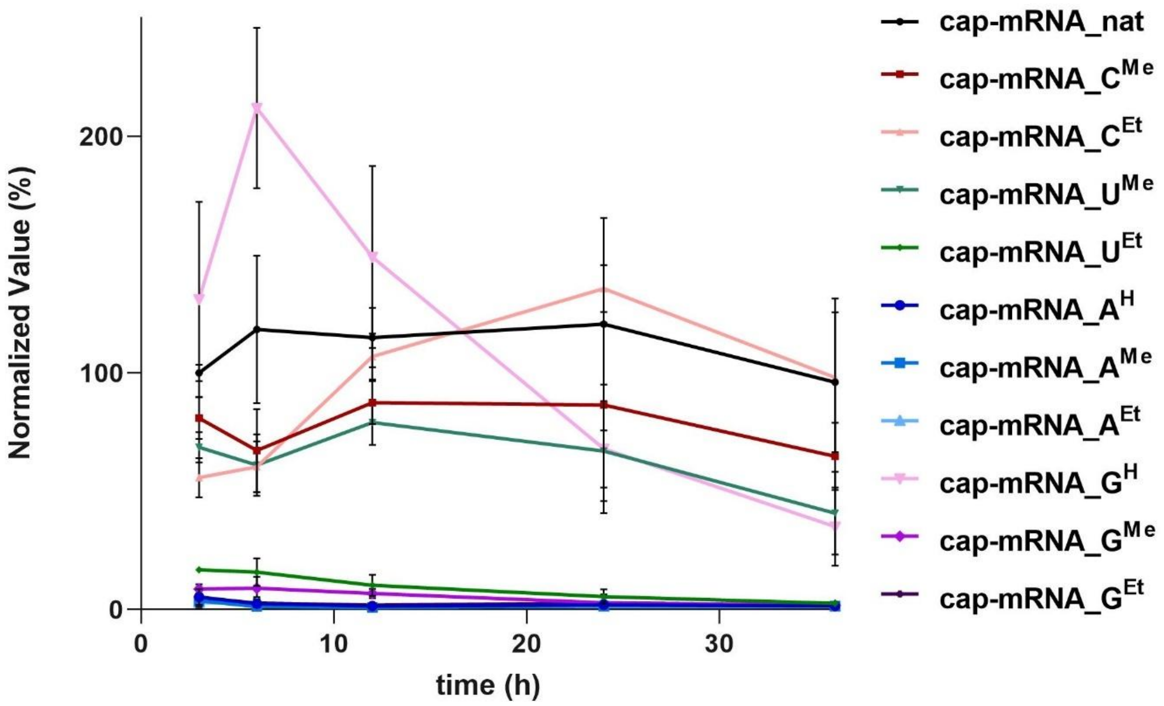

Figure S17: Time-course analysis of *Renilla* luciferase expression in HeLa S3 cells co-transfected with natural (**cap-mRNA_nat**) or modified (**cap-mRNA_N^{R}**) with natural mRNA encoding *Firefly* luciferase (**mRNA_natFF)**, using Lipofectamine MessengerMAX and collected at 3, 6, 12, 24, and 36 h post-transfection. Data are normalized to the luminescence signal of **mRNA_natFF** and **cap-mRNA_nat** at 3 h (set as 100%) and represent mean ± SD from three biological replicates (n = 3), each performed in technical triplicate.

Table S11 Mean of normalized values (%), standard deviation (SD) and standard error of the mean (SEM) from luminescence measurement of *Renilla* luciferase co-transfected with *Firefly* luciferase *in cellulo* for n = 3

	3h			6h			12h		
	Mean	SD	SEM	Mean	SD	SEM	Mean	SD	SEM
**cap-mRNA_nat**	100.00	3.26	1.15	118.44	29.44	10.41	114.91	11.86	4.19
**RNA_C^{Me}**	80.96	8.25	2.92	67.24	16.56	5.85	87.47	8.59	3.04
**RNA_C^{Et}**	55.72	7.88	2.78	60.35	10.17	3.60	106.95	9.25	3.27
**RNA_U^{Me}**	68.55	6.09	2.15	61.14	12.20	4.31	79.08	9.00	3.18
**RNA_U^{Et}**	16.89	1.36	0.48	15.85	5.47	1.93	10.26	4.22	1.49
**RNA_A^{H}**	5.41	3.15	1.12	2.48	1.57	0.56	1.51	1.14	0.40
**RNA_A^{Me}**	3.76	2.34	0.83	1.25	0.87	0.31	0.92	0.65	0.23
**RNA_A^{Et}**	2.98	2.46	0.87	2.58	2.53	0.89	1.19	0.99	0.35
**RNA_G^{H}**	131.13	38.85	13.73	211.97	31.94	11.29	148.93	36.34	12.85
**RNA_G^{Me}**	8.72	1.88	0.66	9.02	4.55	1.61	6.81	1.93	0.68
**RNA_G^{Et}**	5.00	3.01	1.06	2.82	1.72	0.61	1.89	0.50	0.18

	24h			36h		
	Mean	SD	SEM	Mean	SD	SEM
**cap-mRNA_nat**	120.39	42.67	15.09	96.15	27.89	9.86
**RNA_C^{Me}**	88.83	30.95	10.94	64.76	13.39	4.73
**RNA_C^{Et}**	136.62	9.66	3.41	98.06	31.47	11.12
**RNA_U^{Me}**	65.30	22.24	7.86	40.69	16.50	5.83
**RNA_U^{Et}**	5.29	3.03	1.07	2.63	0.84	0.30
**RNA_A^{H}**	1.88	1.45	0.51	1.53	0.82	0.29
**RNA_A^{Me}**	1.42	0.97	0.34	1.49	0.78	0.28
**RNA_A^{Et}**	1.20	0.90	0.32	1.28	0.75	0.27
**RNA_G^{H}**	66.80	26.85	9.49	35.02	15.58	5.51
**RNA_G^{Me}**	2.89	1.19	0.42	1.92	0.67	0.24
**RNA_G^{Et}**	2.75	3.51	1.24	1.84	1.35	0.48

2.8.4 Confluency analysis

Cell proliferation was monitored in real time using the IncuCyte® SX1 Live-Cell Analysis System (Sartorius). Cells were seeded in 24-well plates and transfected with mRNA as described in section 2.8.1. Following transfection, plates were maintained under standard

incubation conditions (37 °C, 5% CO_2) and imaged every four hours over 48 h. Confluency (%) was automatically quantified from phase contrast images using AI confluence segmentation module in IncuCyte® software and plotted over time. Transfection only mRNA encoding *Renilla* luciferase (**Figure S18**), and co-transfection of mRNA encoding *Renilla* luciferase with mRNA encoding *Firefly* luciferase (**Figure S19**).

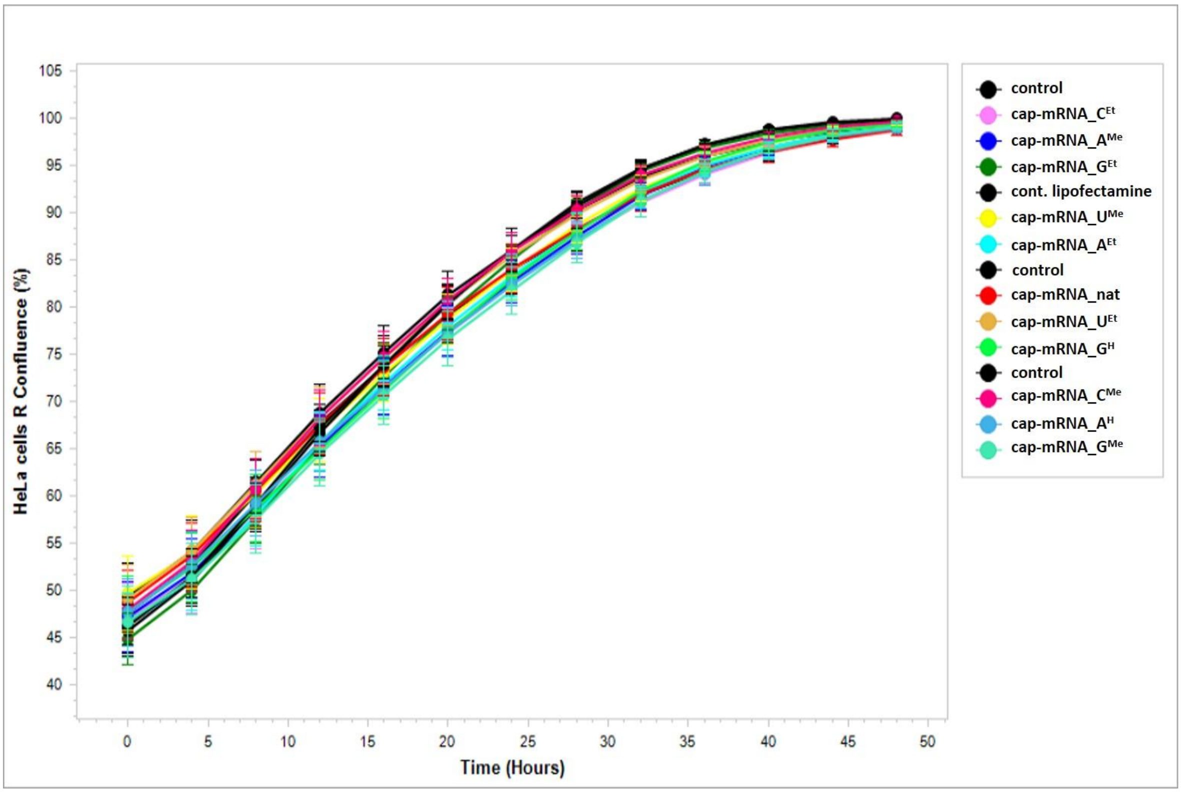

Figure S18: Real-time analysis of cell proliferation following mRNA transfection. HeLa cells were transfected with natural (**cap-mRNA_nat**) or chemically modified mRNAs (**cap-mRNA_N^{R}**) and monitored using IncuCyte. Confluency (%) was quantified from phase contrast images using AI confluence segmentation module in IncuCyte® software over time. Control means untreated cells (no mRNA or Lipofectamine MessengerMax) and control lipofectamine means cells treated with transfection reagent but no mRNA.

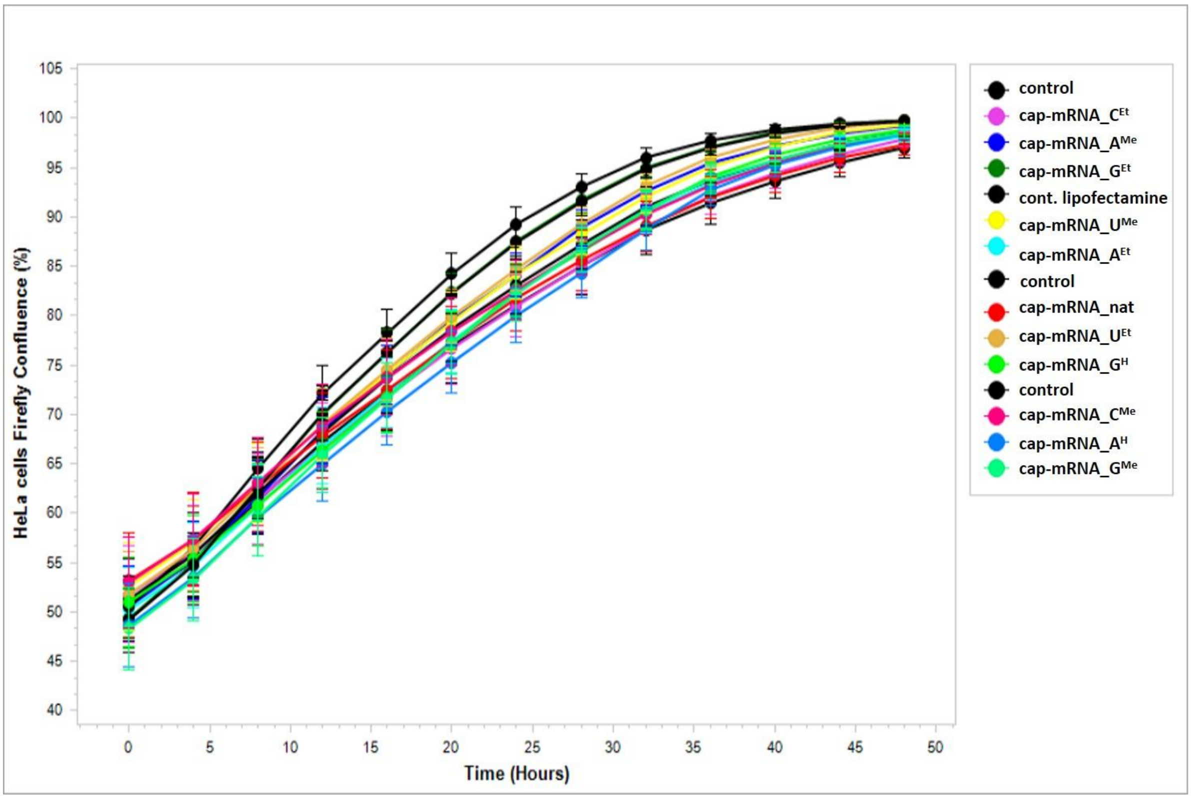

Figure S19: Real-time analysis of cell proliferation following mRNA transfection. HeLa cells were transfected with natural (**cap-mRNA_nat**) or chemically modified mRNAs (**cap-mRNA_N^{R}**) and natural mRNA encoding *Firefly* luciferase **mRNA_natFF** and monitored using IncuCyte. Confluency (%) was quantified from phase contrast images using AI confluence segmentation module in IncuCyte® software over time. Control means untreated cells (no mRNA or Lipofectamine MessengerMax) and control lipofectamine means cells treated with transfection reagent but no mRNA.

2.9 RNA quantification

2.9.1 Synthesis of RNA by PEX

The reaction mixture (10 µL) contained RNA primer LT25TH-Cy5 (4 µM), template FVL-A (4.8 µM), TGK DNA polymerase (5 µM), two natural NTPs (0.8 mM), either natural or modified **A^{H}TP** or **G^{H}TP** (0.8 mM) in enzyme reaction buffer (1X). The reaction mixture was incubated for 6 h at 60°C in a thermal cycler. The PEX reaction was stopped by addition of 2X gel loading dye and heated for 5 min at 95 °C for preparation of gel or by adding TurboDNase (1 µl) and heated for 30 min at 37 °C, purified with QIAquick nucleotide removal kit following suplier's protocol (**Figure S20**).

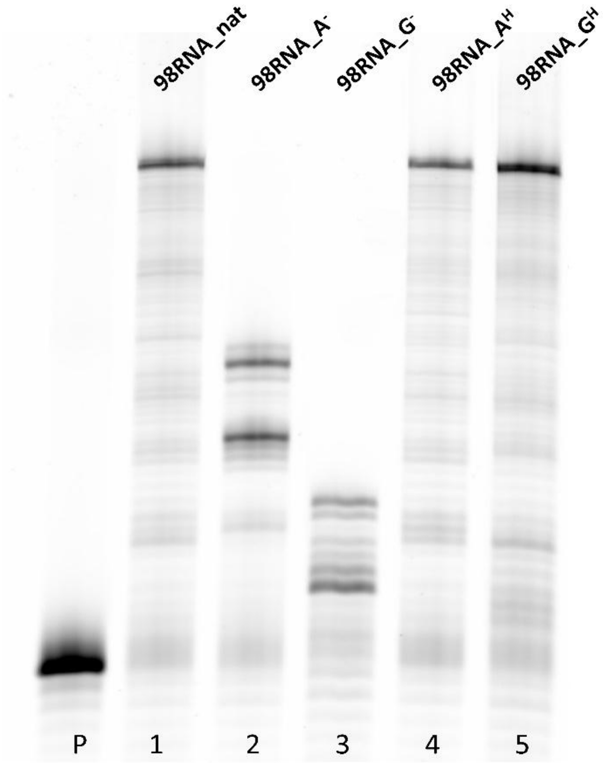

Figure S20: 20% dPAGE analysis of PEX reaction. (P) Primer LT25TH-Cy5 (1) Positive control, all natural NTPs; (2,3) negative controls, three natural NTPs, without NTP or **N^{R}TP** of interest; (4,5) three natural NTPs with the modified **N^{R}TP** of interest.

2.9.2 Fluorescence measurements

Fluorescence intensity of different concentration of natural **98RNA_nat** and modified **98RNA_A^{H}** and **98RNA_G^{H}** synthesized as described in section **2.8.1** was measured using a TECAN Spark® Multimode Microplate Reader. The calibration curve was generated based on fluorescence intensity data measured from **98RNA_nat** (**Figure S21**) and the fluorescence intensity values of **98RNA_A^{H}** and **98RNA_G^{H}** were extrapolated using the equation derived from the calibration curve. The calculated concentrations were compared to those measured using a Nanodrop spectrophotometer (**Table S12**). The ratio between the calculated [c_1] and measured concentrations [c_2] was close to 1, indicating that the Nanodrop measurement provides a reliable RNA concentration.

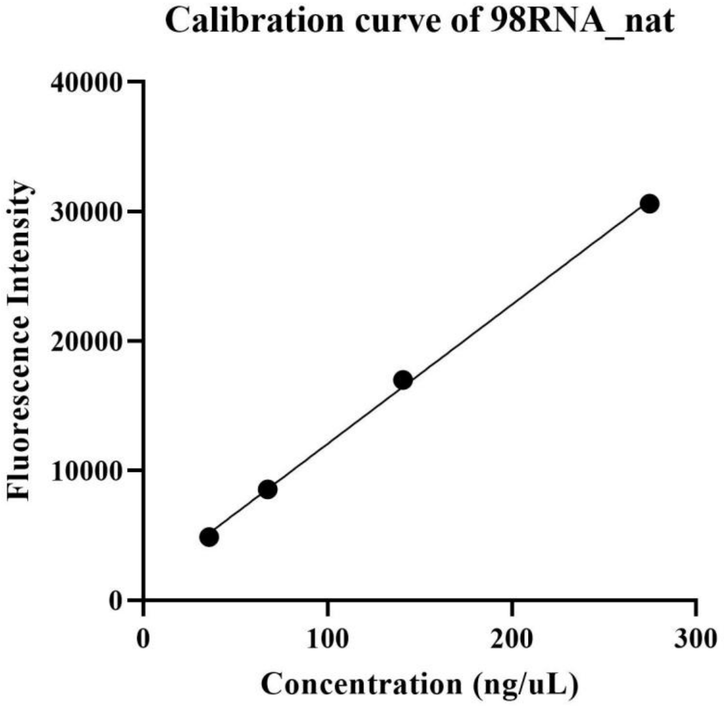

Figure S21: Calibration curve generated as measurement of fluorescence intensity versus different concentration (ng/µl) of natural RNA (**98RNA_nat)** as shown in **Table S12**.

Table S12 Fluorescence measurement and calculated/measured concentrations.

98RNA_16A^H				98RNA_18G^H			
Fluorescence intensity	Ccalc.[ng/µl] [c_1]	Cmeas.[ng/µl] [c_2]	[c_1]/[c_2]	Fluorescence intensity	Ccalc.[ng/µl] [c_1]	Cmeas.[ng/µl] [c_2]	[c_1]/[c_2]
29675	263.8	257.8	1	31760	283.2	317	0.9
16356	139.8	132	1.1	20009	173.8	171.9	1
9200	73.2	64.5	1.1	10406	84.4	89.2	0.9
4565	30	33.5	0.9	5479	38.5	45.5	0.8

Ccalc. [c_1] from TECAN Spark® Multimode Microplate Reader; Cmeas. [c_2] from Nanodrop spectrophotometer.

2.10 Molecular dynamics

2.10.1 Computational models

The sgRNA–Cas9–DNA complexes were modelled based on the chain A of the crystal structure PDB:4OO8.[11] Three different systems with **sgRNA_nat**, **sgRNA_A^{H}**, and **sgRNA_A^{Me}** were prepared. The starting solution structure was obtained from charmm-gui,[12] and the missing hydrogens and protein loops were added within it. All the systems were solvated in cubic simulation boxes of 16X16X16 nm3 with approximately 125000 tip3p water molecules.[13] All the models were parameterized with the Amber-compatible OL3 force field[14] for the RNA, OL15[15] for the DNA, and ff14SB[16] for the protein. 150 mM of K+ and Cl- ions were added, together with the needed excess of K+ ions to ensure overall charge neutrality. The topologies were translated to gromacs format with Acpype.[17] The parameters for the modified **sgRNA_A^{Me}** and **sgRNA_A^{H}** bases were generated using Antechamber[18] with the GAFF force field[19] and AM1-BCC charges,[20] in a similar manner as done by Zahorska et al.[21] To maintain compatibility with standard nucleic acid topologies, all parameters associated with the phosphate backbone and pentose sugar rings were restored to the values from the OL13 force field. Only the parameters of the modified adenine nucleobases were retained, with their AM1-BCC charges redistributed across the nucleobase to preserve overall charge neutrality. Although the experimental first 20 nucleotides differ from the simulated structure, they are not vital for sgRNA recognition by Cas9 (schematic representation, **Figure S23**).

2.10.2 Simulation protocol

All systems were shortly energy minimized with restraints in the protein, RNA, and DNA. Then, they were submitted to a series of three 100 ps equilibrations with decreasing restraints (1000, 250 and 10 kJ/mol nm2 for DNA and RNA and 1000, 250 and 250 kJ/mol nm2 for the protein) in the NVT ensemble. From the resulting structures, three replicates for each system were prepared. Each of the replicates was submitted to a short minimization and equilibration in the NVT ensemble without restraints. The starting velocities were randomly drawn from a Maxwell-Boltzmann distribution a 310 K to ensure replica independence. Subsequently, production molecular dynamics simulations were conducted for 1 μs. The temperature was kept at 310 K with the V-rescale thermostats and a 1 ps coupling constant.[22] Pressure was regulated at 1 bar using the C-rescale barostat, with a coupling time of 5 ps.[23] Short-range electrostatic interactions were truncated at 1.0 nm, while long-range electrostatics were treated using the

particle mesh Ewald (PME) method.[24] A 1.0 nm cut-off was also employed for Lennard-Jones interactions. To maintain water geometry, the SETTLE algorithm[25] was applied, and all other hydrogen-involving covalent bonds were constrained using the LINCS algorithm.[26,27] Simulations were performed using GROMACS version 2023.[28] All relevant simulation files, including input and output data necessary for reproducibility, are accessible via Zenodo at DOI: 10.5281/zenodo.16085542.

2.10.3 Simulation analysis

The trajectories were analyzed using the MDAnalysis Python package.[29] The per-residue root mean squared deviation (RMSD) of the RNA backbone was calculated using the phosphate atoms with respect to the reference PDB:4oo8 after addition of hydrogens and missing loops. The contact probability between the RNA residues 71-78 and the S719, L720, H721, and E722 was calculated as the percentage of frames in which at least one atom from both selections is at 5 Å or less from another atom of the other selection. All the results shown are average values between replicates. The simulation snapshots are presented using Pymol[30] (**Figure S22** and **Figure 6**).

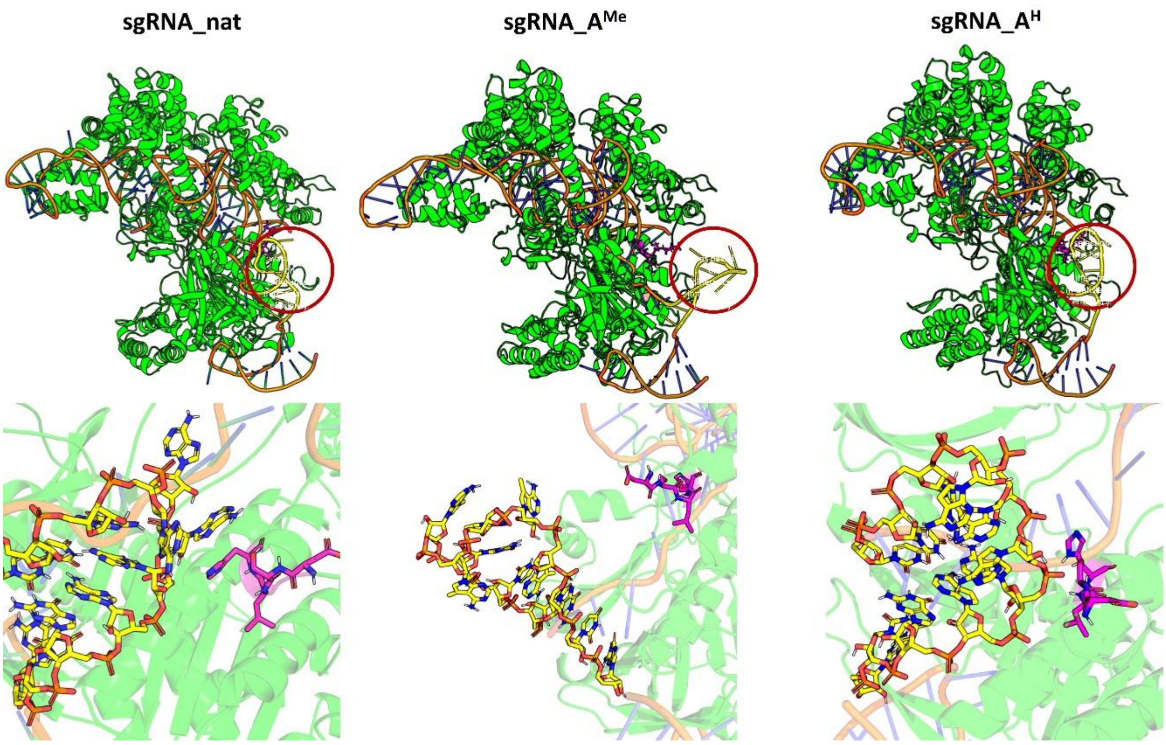

Figure S22: (A) Top: representative structure from the molecular dynamics of the Cas9 protein (green) with **sgRNA_nat, sgRNA_A^{Me}** and **sgRNA_A^{H}**. RNA Stem Loop 2 region, employed in previous analysis, is highlighted in yellow. The four selected residues from the protein are

shown in magenta. Bottom: zoom highlighting the interaction between the Stem Loop 2 and the Cas9.

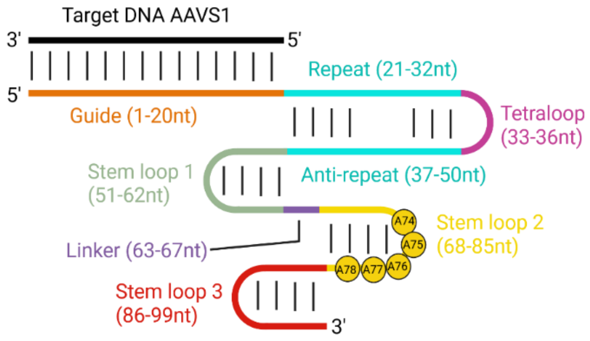

Figure S23: Schematic representation of the sgRNA:target DNA complex, also based on a previously published work.[11] In yellow is highlighted the adenosines situated in the Stem Loop 2, the most affected RNA region.

3 Copies of NMR spectra

3.1 ^{1}H and ^{13}C NMR spectra of 7-Ethyl-7-deazaadenosine (15, A^{Et})

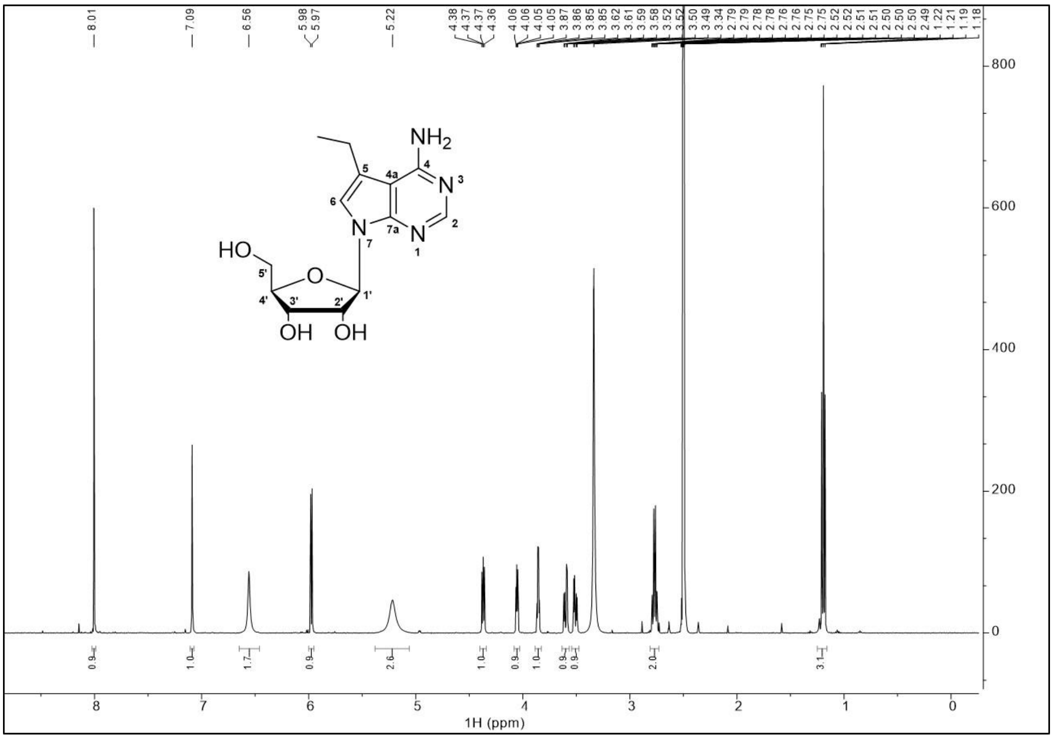

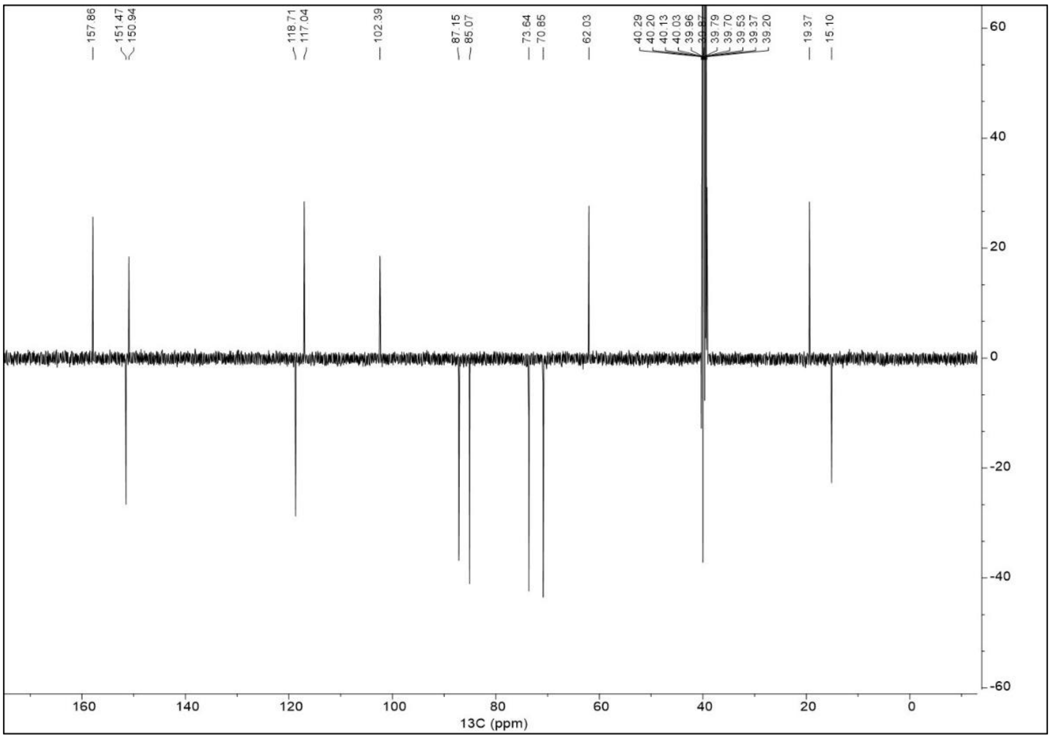

3.2 ^{1}H and ^{13}C NMR spectra of 7-Ethyl-7-deazaguanosine (16, G^{Et})

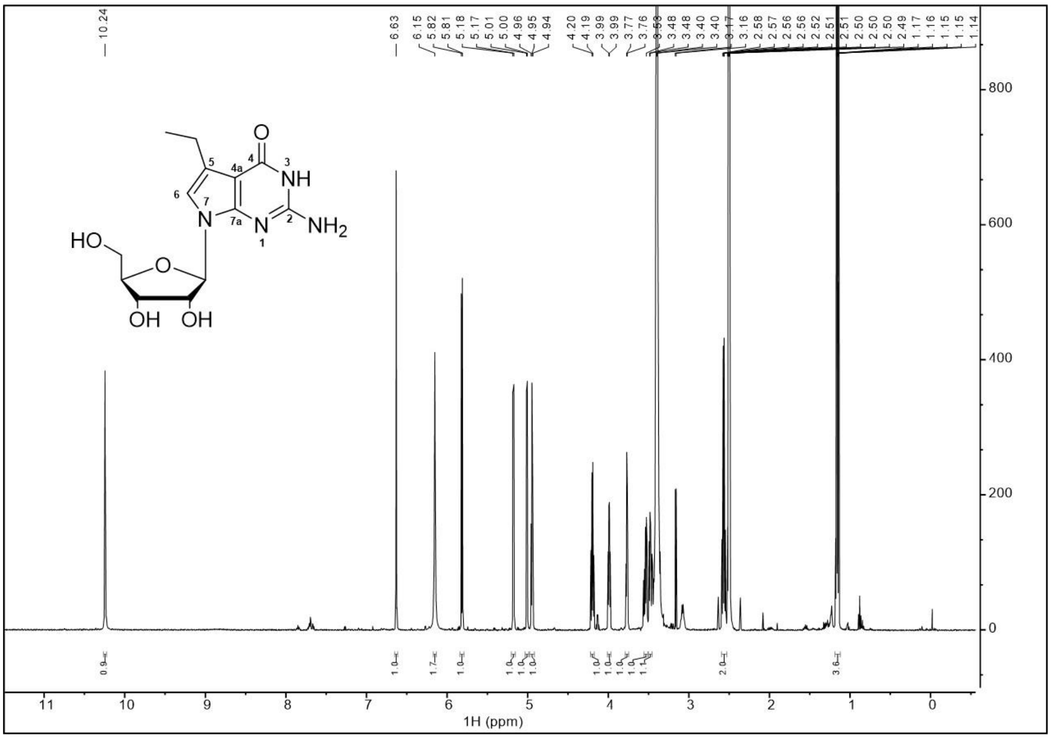

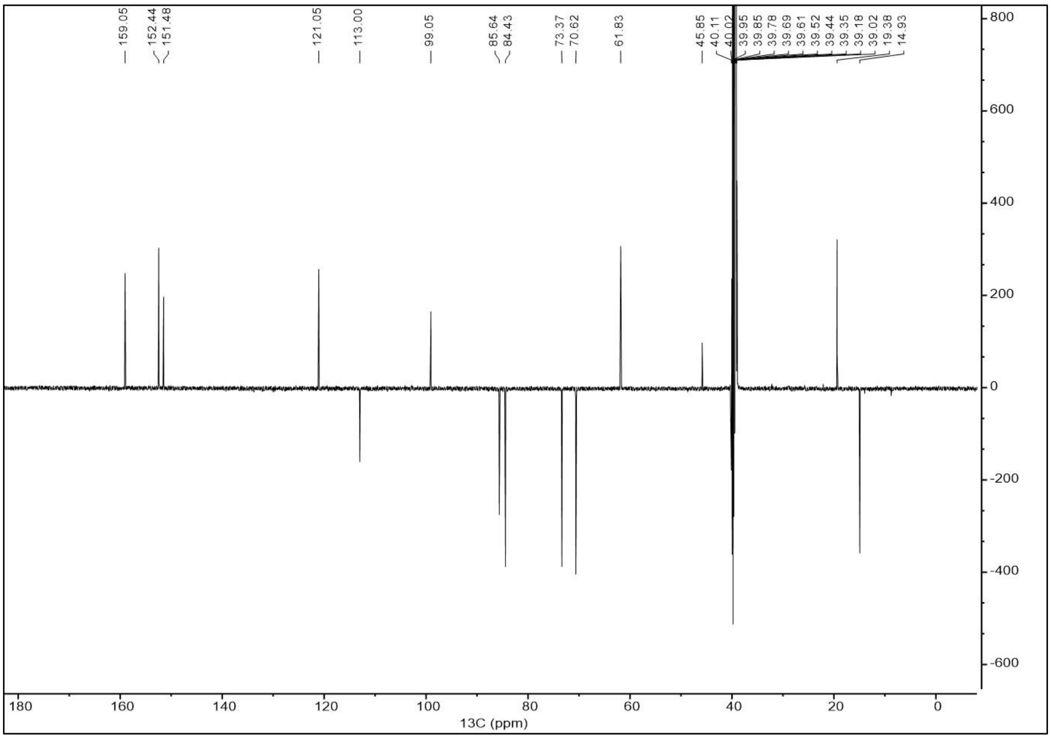

3.3 ^{1}H, ^{13}C and ^{31}P {^{1}H} NMR spectra of 5-Ethylcytidine 5'-*O*-triphosphate (17, $C^{Et}TP$)

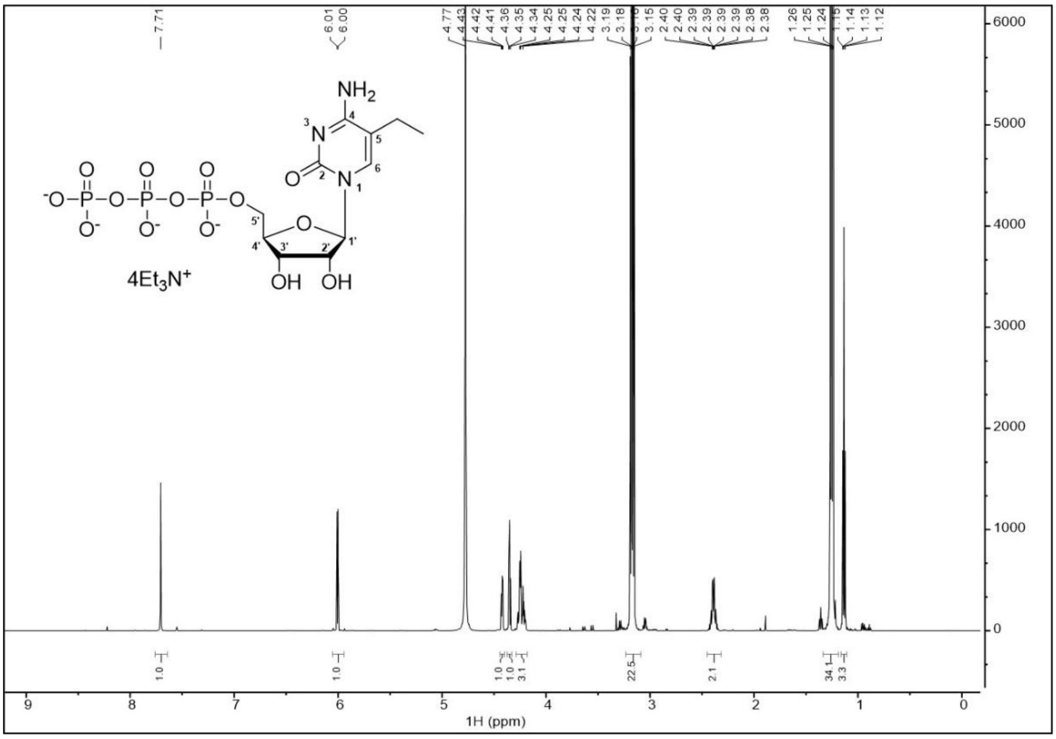

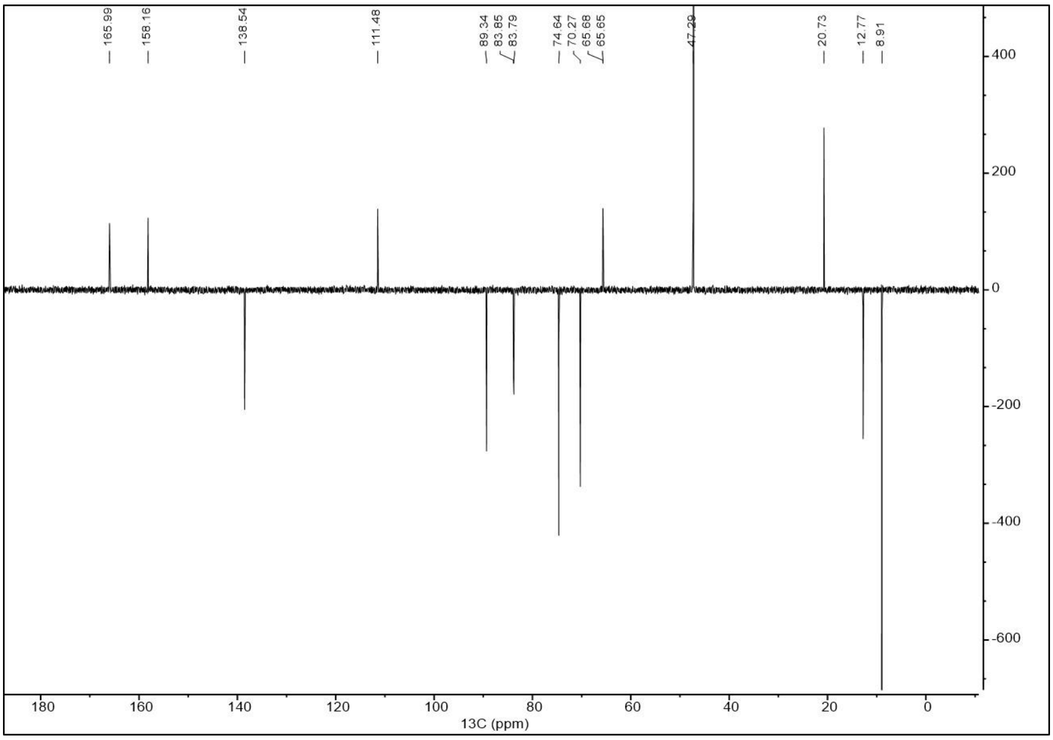

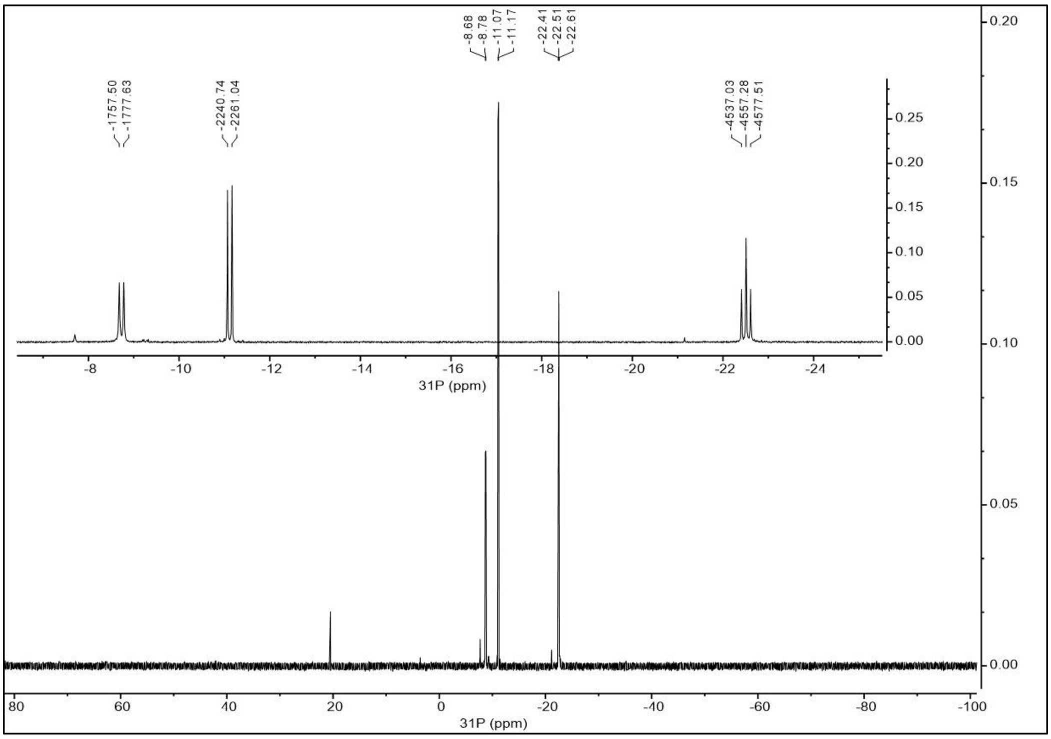

3.4 ^{1}H, ^{13}C and ^{31}P {^{1}H} NMR spectra of 5-Ethyluridine 5'-*O*-triphosphate (18, U^{Et}TP)

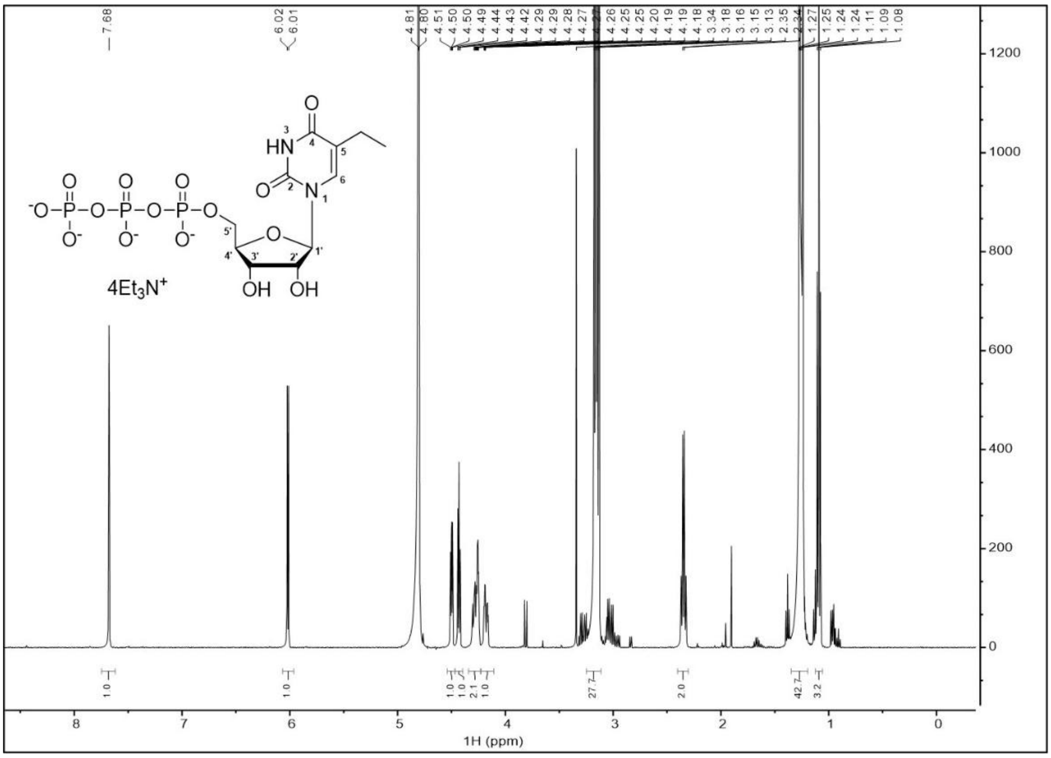

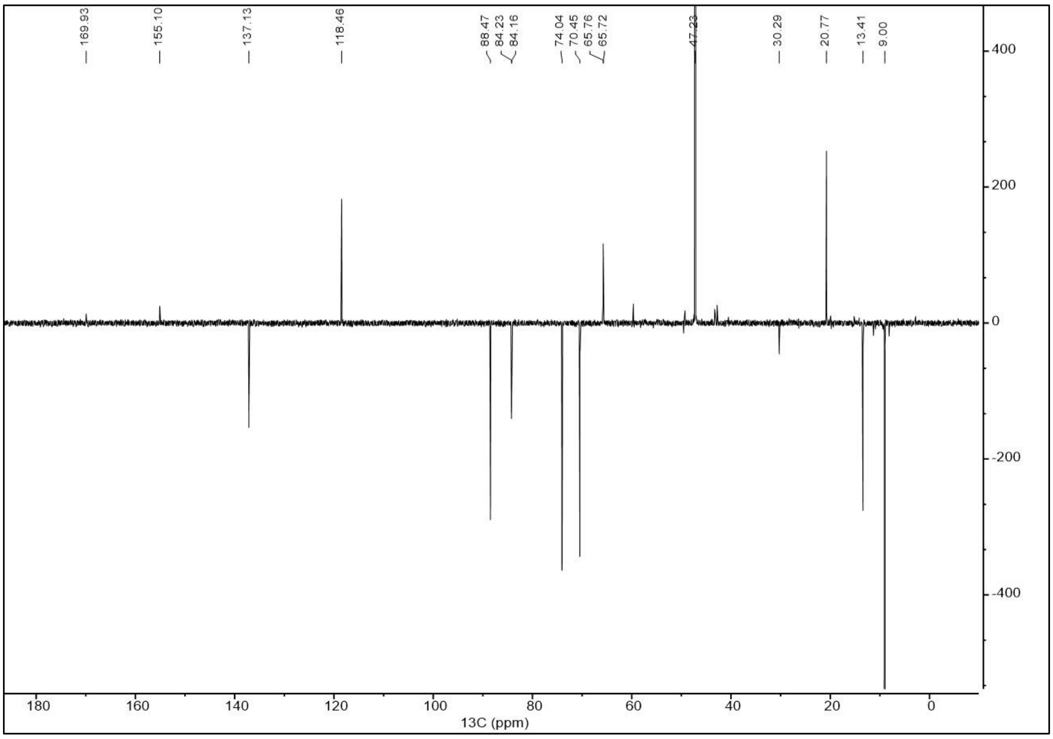
169.93
155.10
137.13
118.46
88.47
84.23
84.16
74.04
70.45
65.76
65.72
47.23
30.29
20.77
13.41
9.00
13C (ppm)

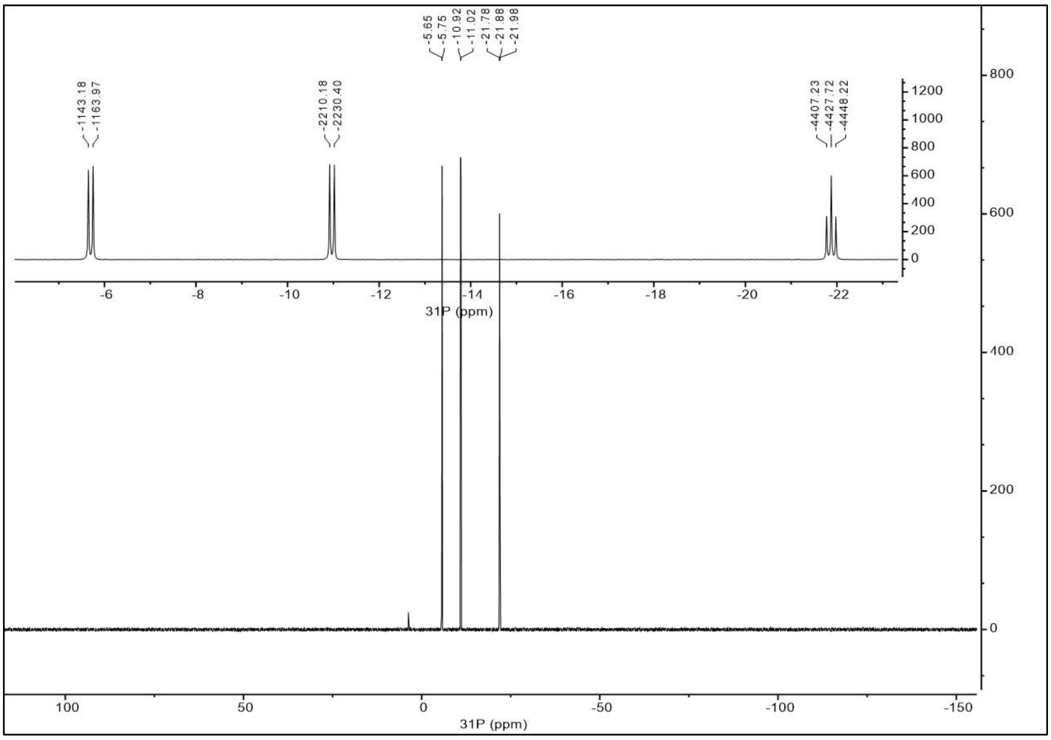
-5.65
-5.75
-10.92
-11.02
-21.78
-21.88
-21.98
-1143.18
-1163.97
-2210.18
-2230.40
-4407.23
-4427.72
-4448.22
31P (ppm)
31P (ppm)

3.5 ^{1}H, ^{13}C and ^{31}P {^{1}H} NMR spectra of 7-Ethyl-7-deazaadenosine 5'-*O*-triphosphate (19, $A^{Et}TP$)

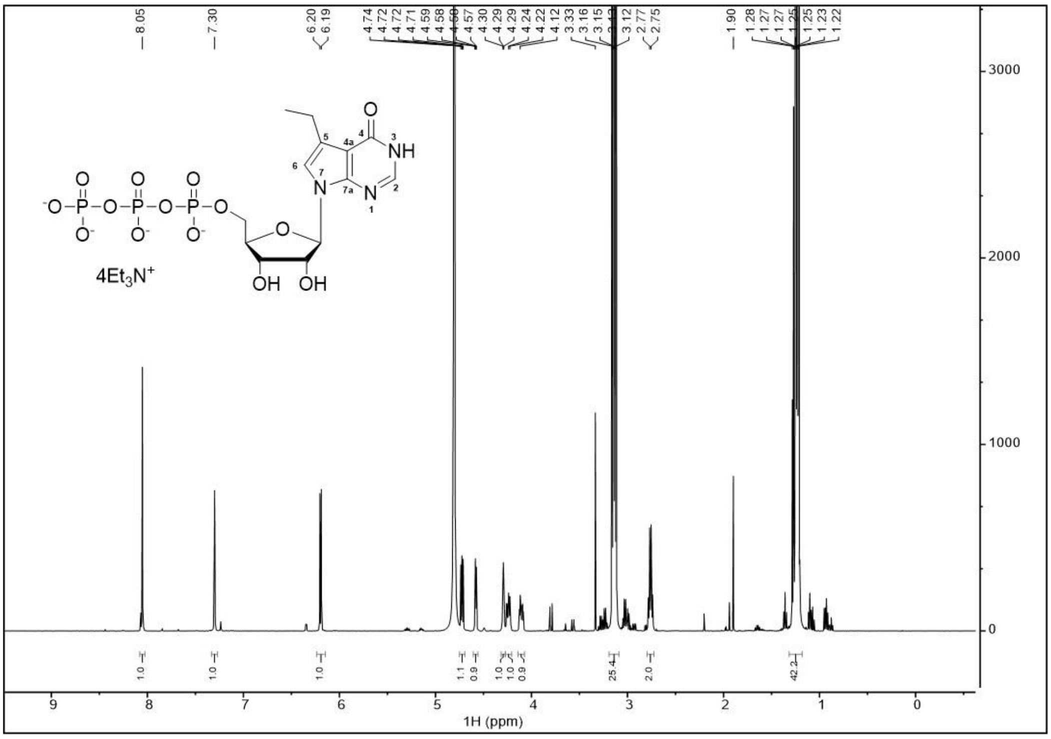

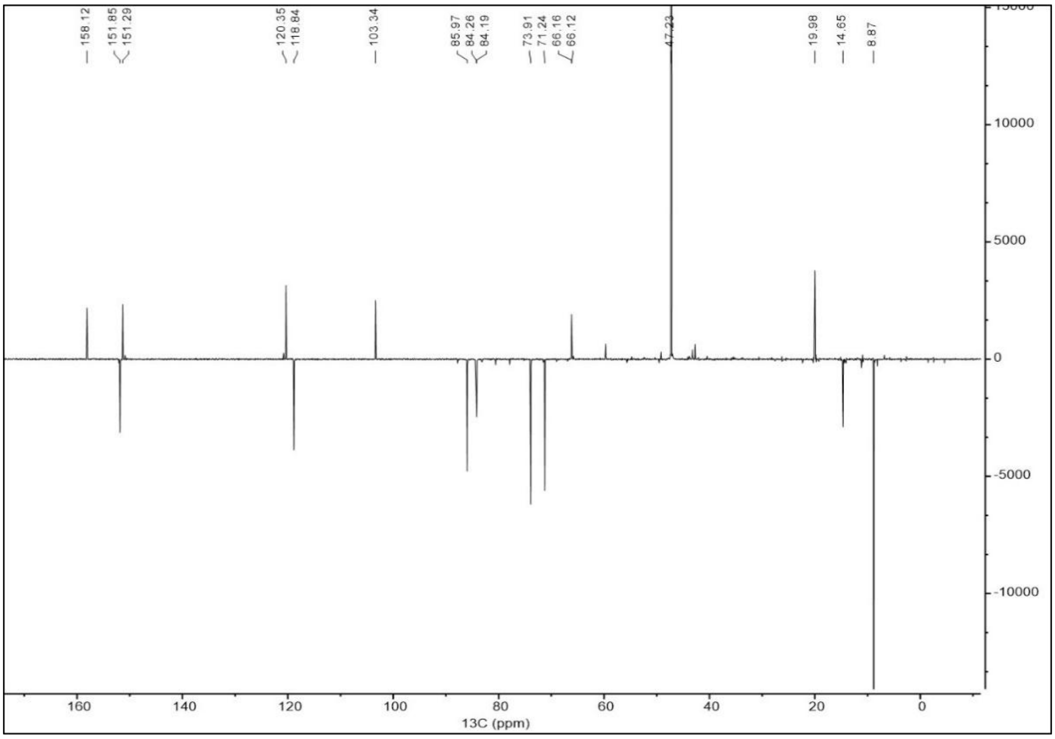

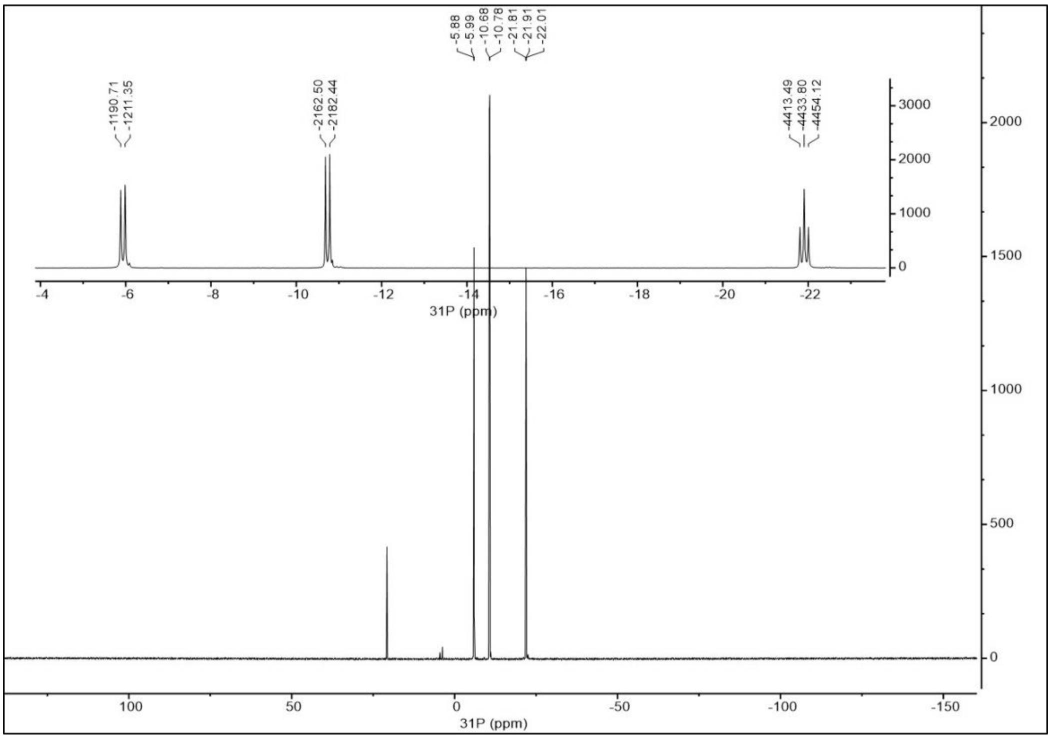

3.6 ^{1}H, ^{13}C and ^{31}P {^{1}H} NMR spectra of 7-Ethyl-7-deazaguanosine 5'-*O*-triphosphate (20, G^{Et}TP)

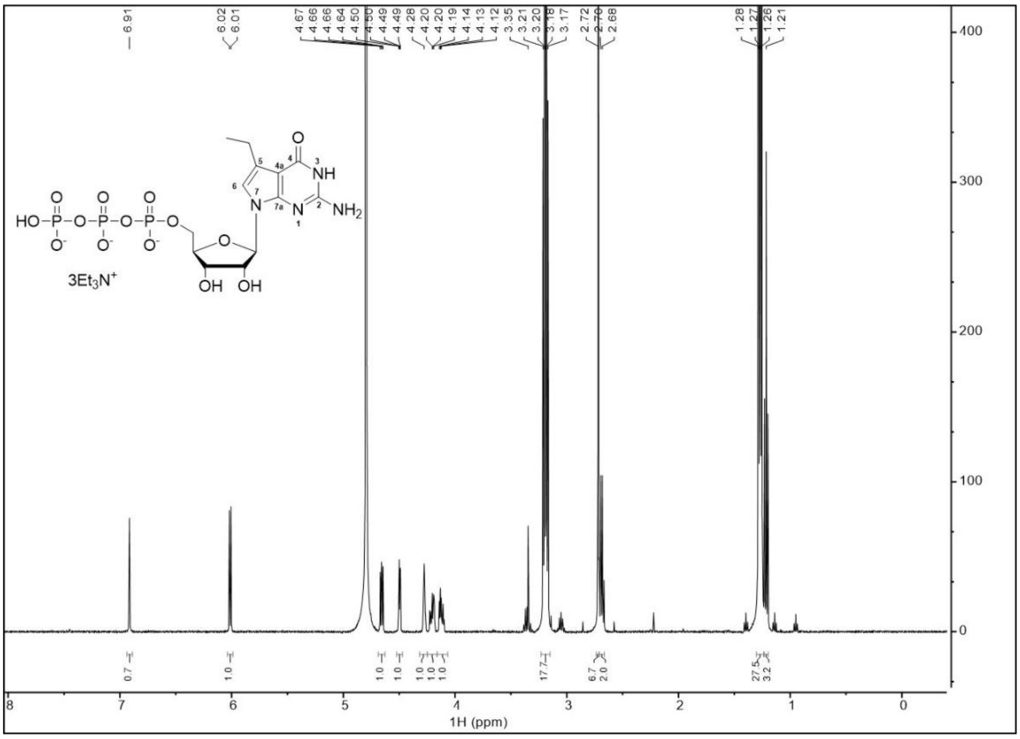

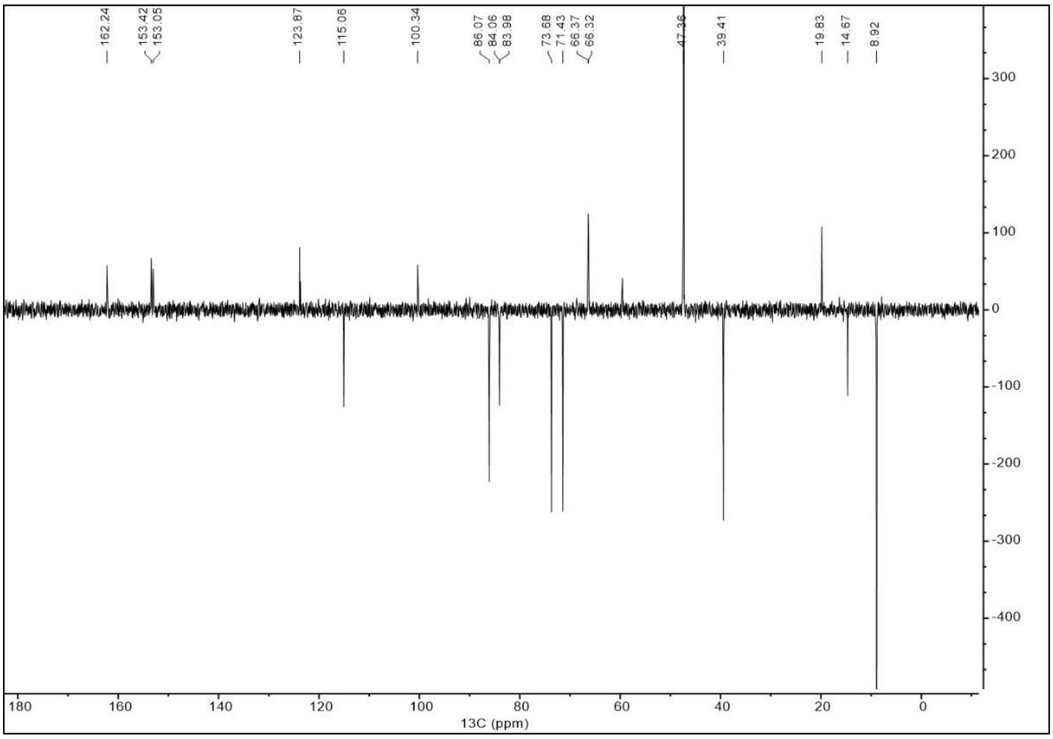
162.24
153.42
153.05
123.87
115.06
100.34
86.07
84.06
83.98
73.68
71.43
66.37
66.32
47.36
39.41
19.83
14.67
8.92
13C (ppm)

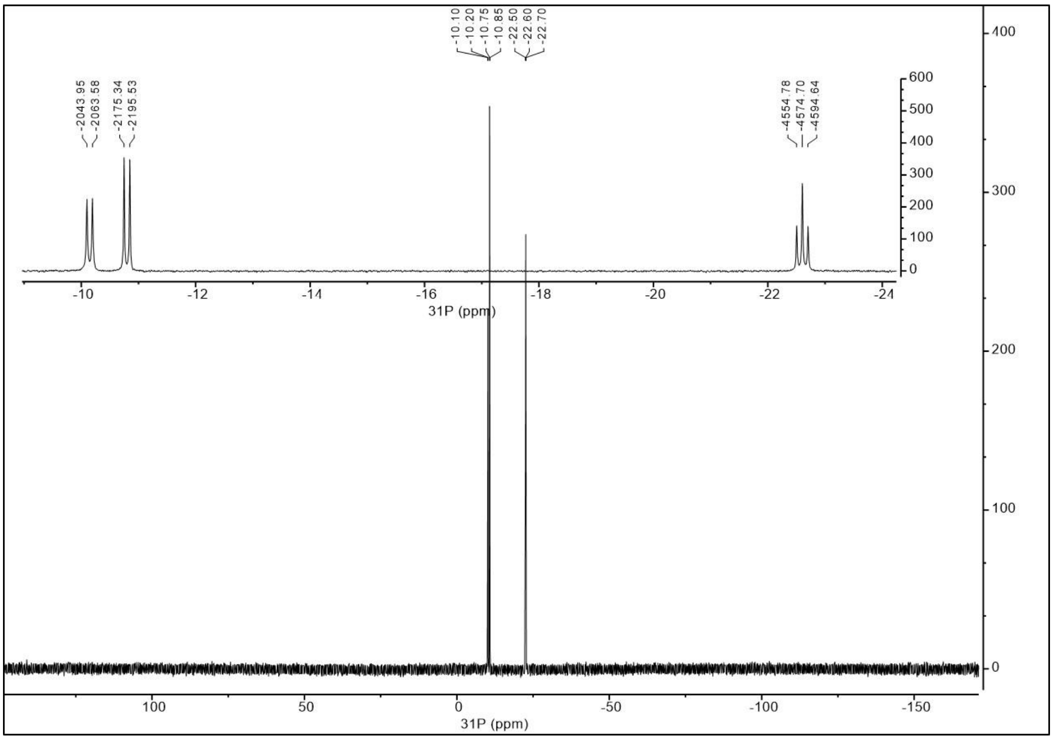
-10.10
-10.20
-10.75
-10.85
-22.50
-22.60
-22.70
-2043.95
-2063.58
-2175.34
-2195.53
-4554.78
-4574.70
-4594.64
31P (ppm)

4 Copies of MS ESI- spectra of nucleosides and nucleotides

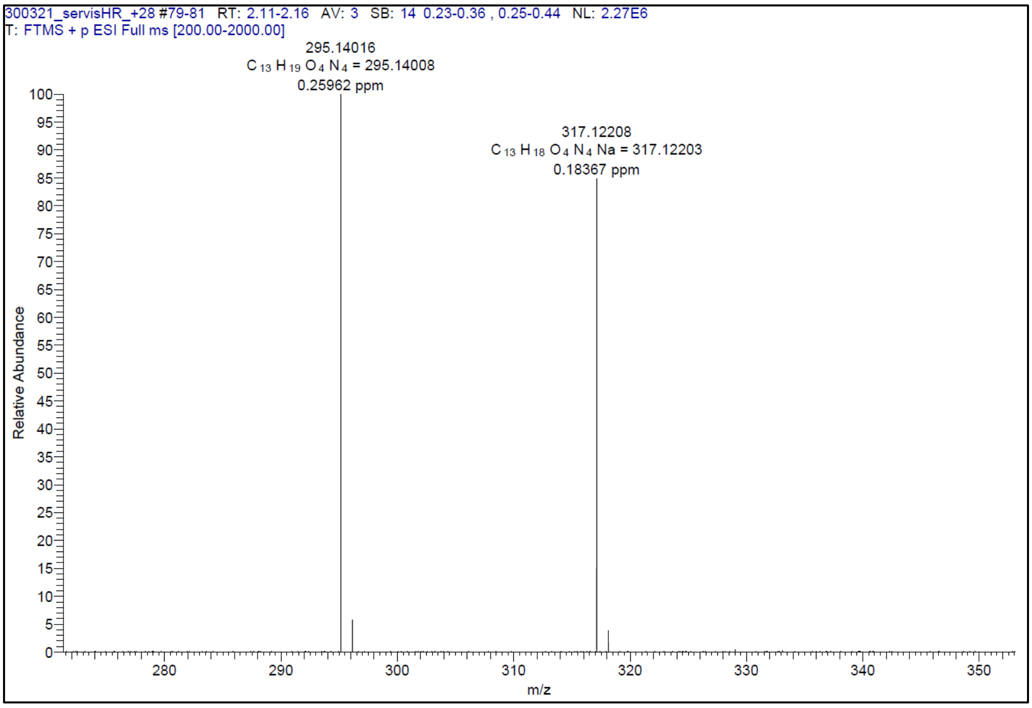

Figure S24: HR MS (ESI) of **A^{Et}**: [M + Na]$^{+}$: calcd 295.1401; found 295.1402, for [M + Na]$^{+}$: calcd 317.1220; found 317.1201.

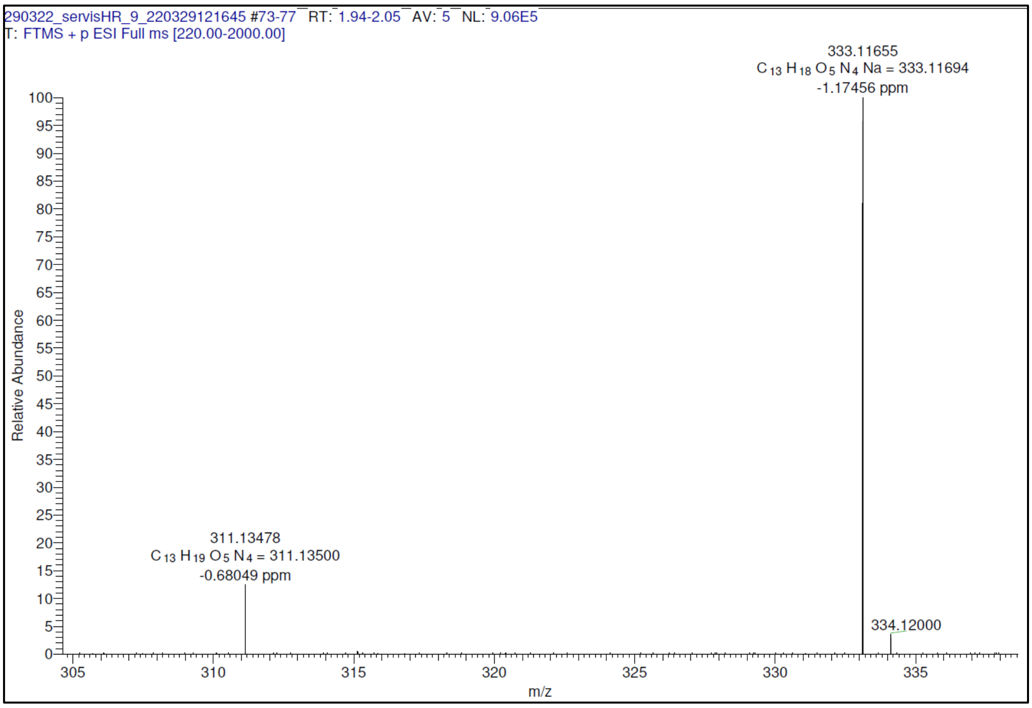

Figure S25: HR MS (ESI) of **G^{Et}**: [M + H]$^{+}$: calcd 311.1350; found 311.1348; for [M + Na]$^{+}$: calcd 333.1169; found 333.1165.

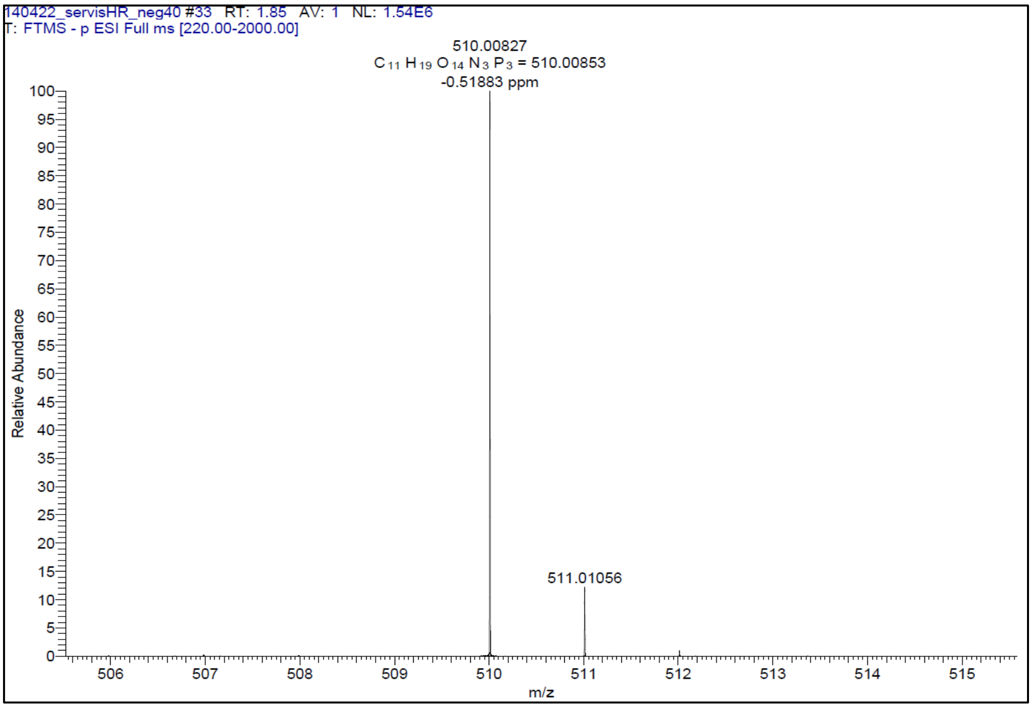

Figure S26: HR MS (ESI) OF **C^{Et}TP**: [M – H]$^-$: calcd 510.0085; found 510.0083.

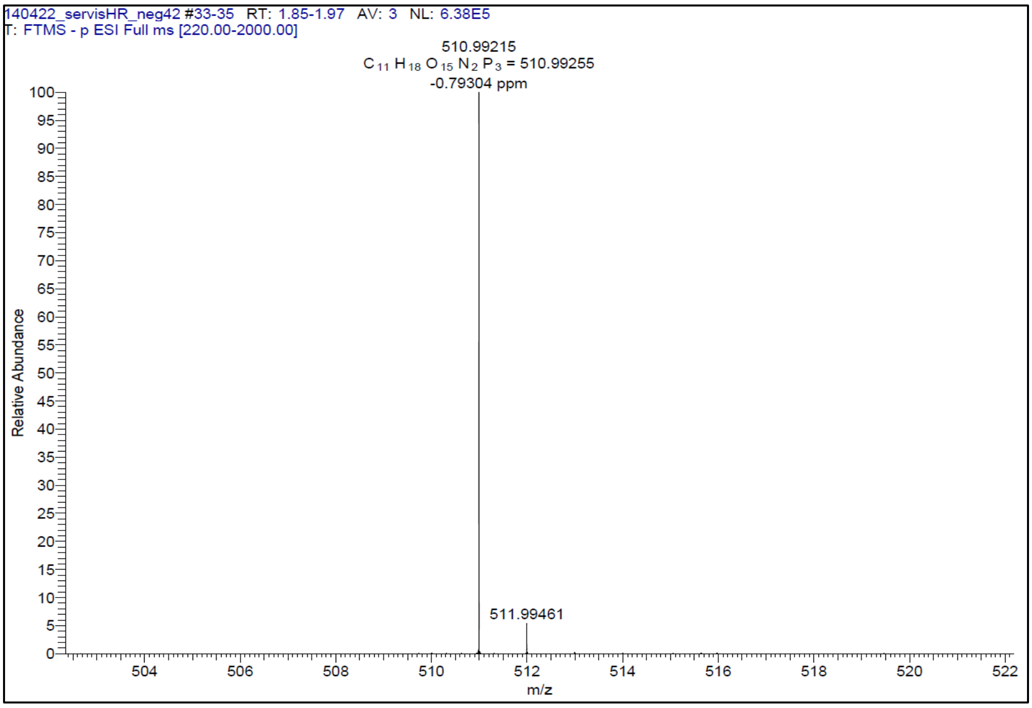

Figure S27: HR MS (ESI) of U^{Et}TP [M – H]$^-$: calcd 510.9925; found 510.9921.

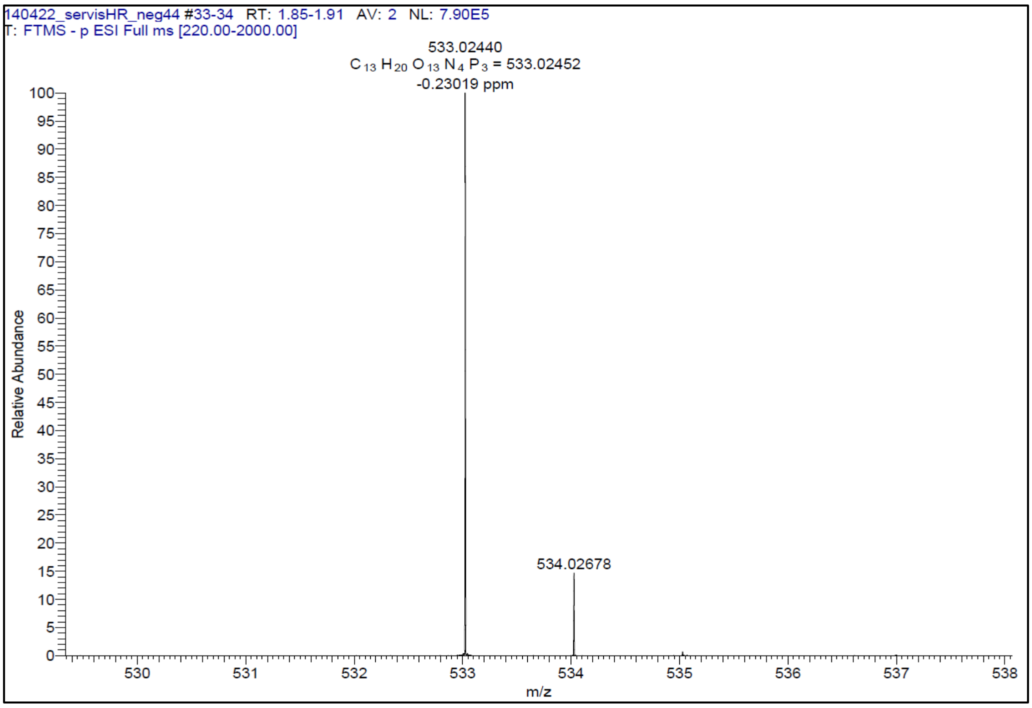

Figure S28: HR MS (ESI) of A^{Et}TP: $[M-H]^-$: calcd 533.0245; found 533.0244.

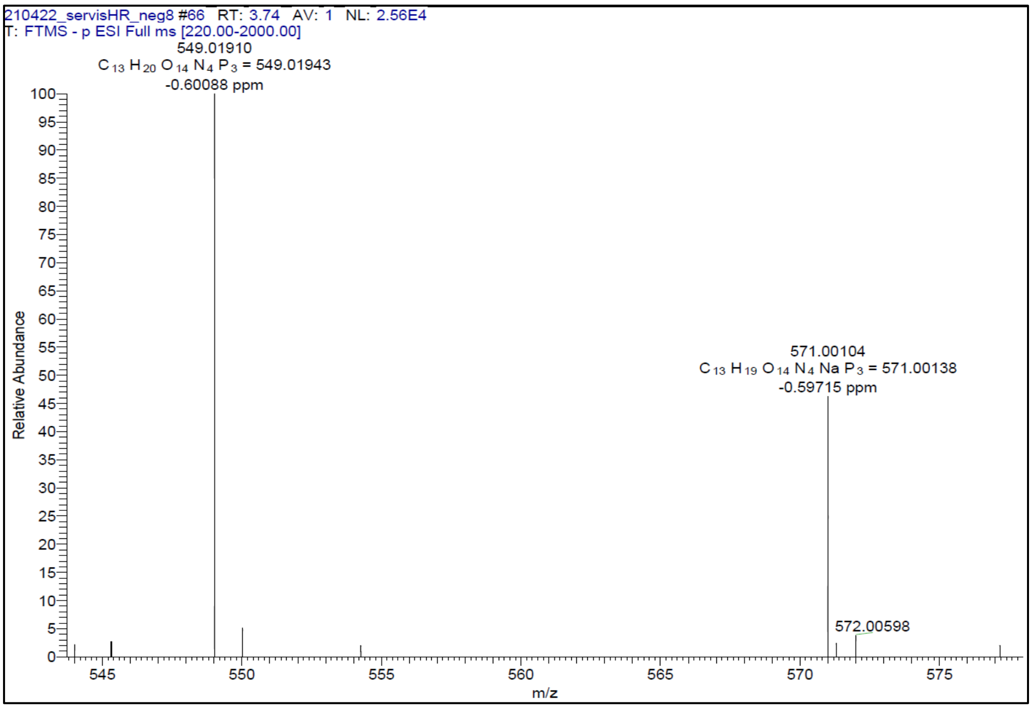

Figure S29: HR MS (ESI) of G^{Et}TP: $[M-H]^-$: calcd 549.0194; found 549.0191, for $[M+\mathrm{Na}-2\mathrm{H}]^-$: calcd 571.0014; found 571.0010.

5 Copy of LC-ESI Spectra of Oligonucletotides

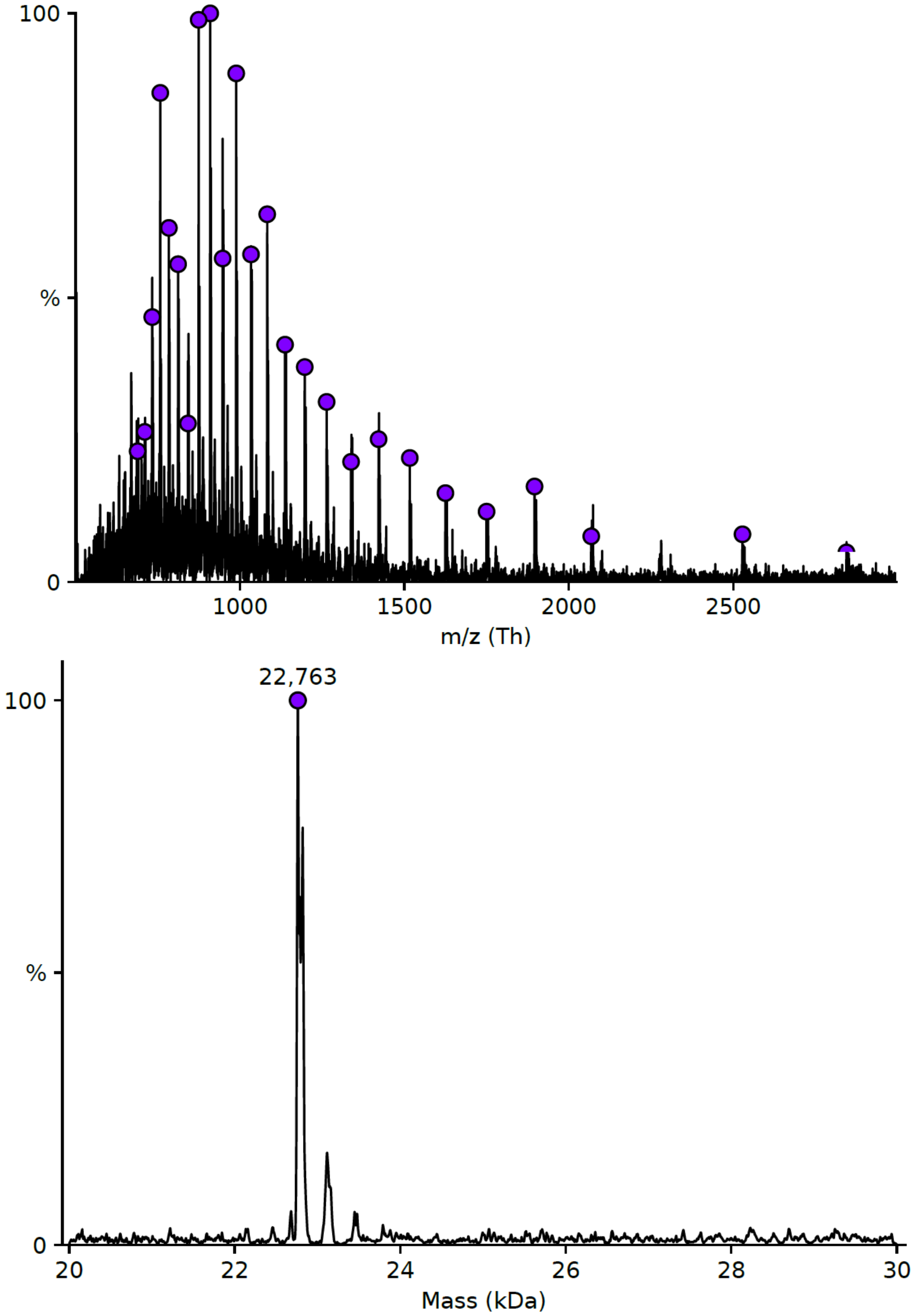

Figure S30: Mass spectra of **70RNA_uncap**. a) raw spectrum. b) deconvoluted spectrum; calculated mass: 22763 Da, found mass: 23763 Da, Δ = 0 Da (**70RNA_uncap**).

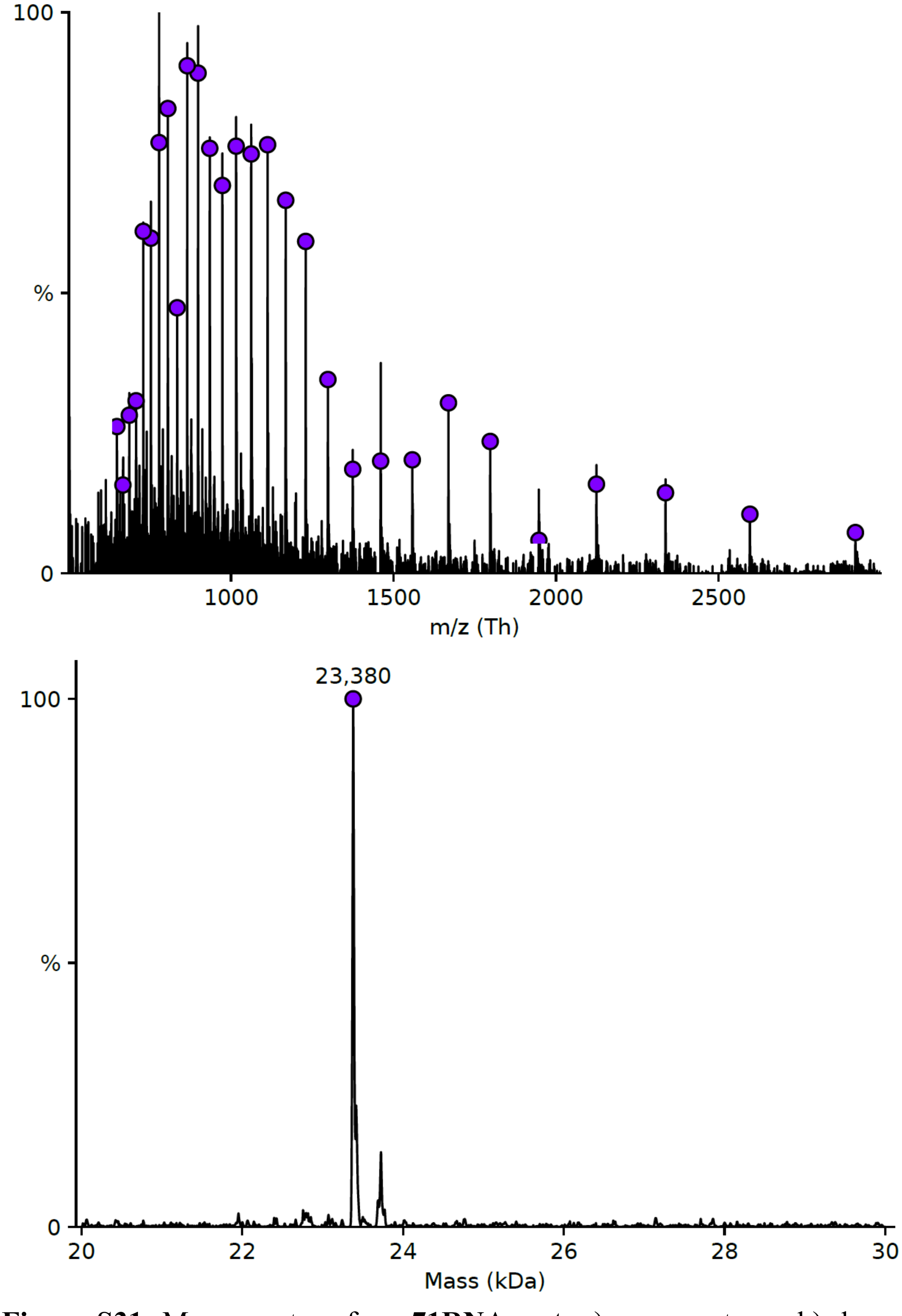

Figure S31: Mass spectra of **cap71RNA_nat**. a) raw spectrum. b) deconvoluted spectrum; calculated mass: 23378 Da, found mass: 23380 Da, Δ = 2 Da (**cap71RNA_nat**).

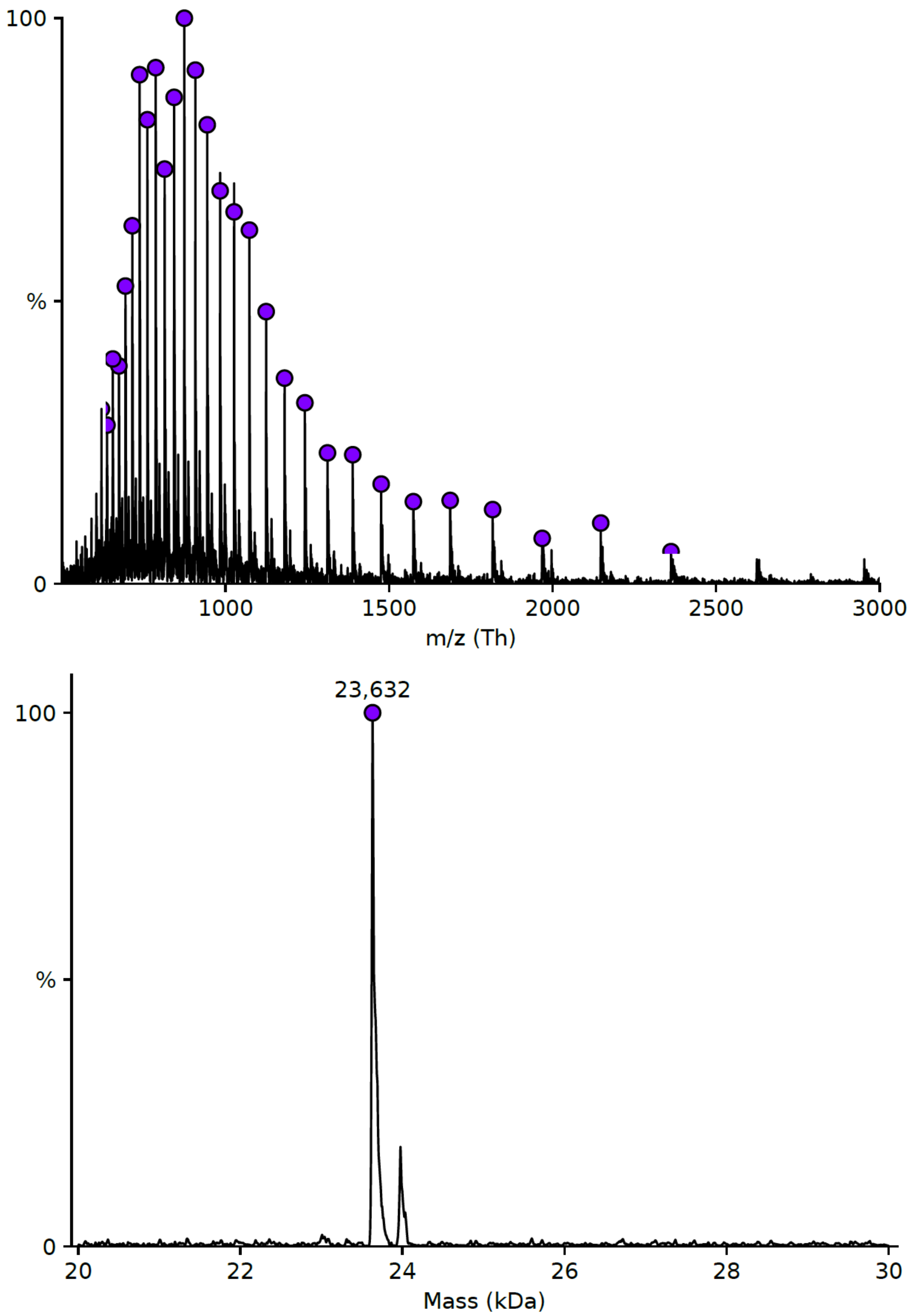

Figure S32: Mass spectra of **cap71RNA_C^{Me}**. a) raw spectrum. b) deconvoluted spectrum; calculated mass: 23630 Da, found mass: 23632 Da, Δ = 2 Da (**cap71RNA_C^{Me}**).

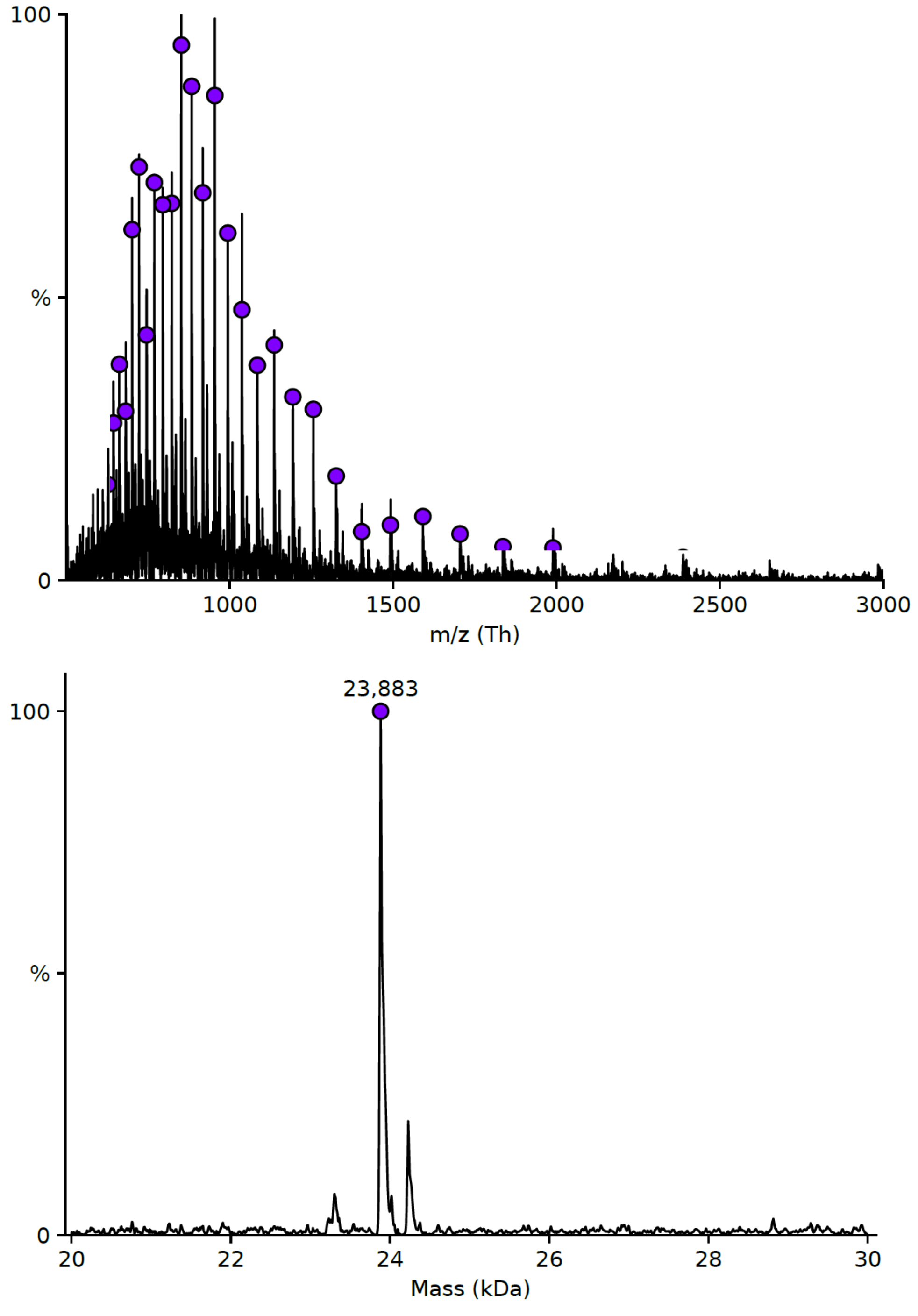

Figure S33: Mass spectra of **cap71RNA_C^{Et}**. a) raw spectrum. b) deconvoluted spectrum; calculated mass: 23882 Da, found mass: 23883 Da, Δ = 1 Da (**cap71RNA_C^{Et}**).

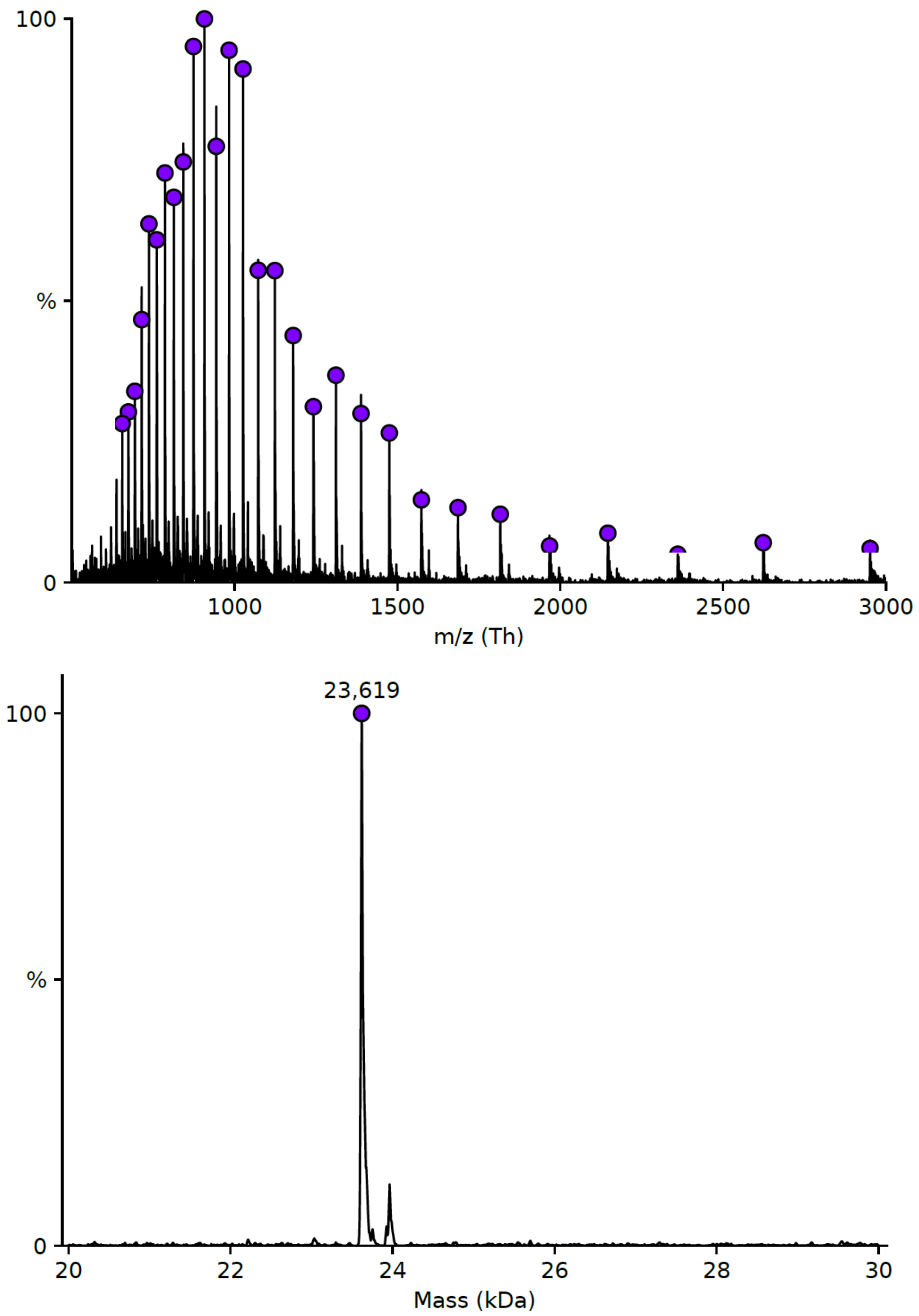

Figure S34: Mass spectra of **cap71RNA_U^{Me}**. a) raw spectrum. b) deconvoluted spectrum; calculated mass: 23616 Da, found mass: 23619 Da, Δ = 3 Da (**cap71RNA_U^{Me}**).

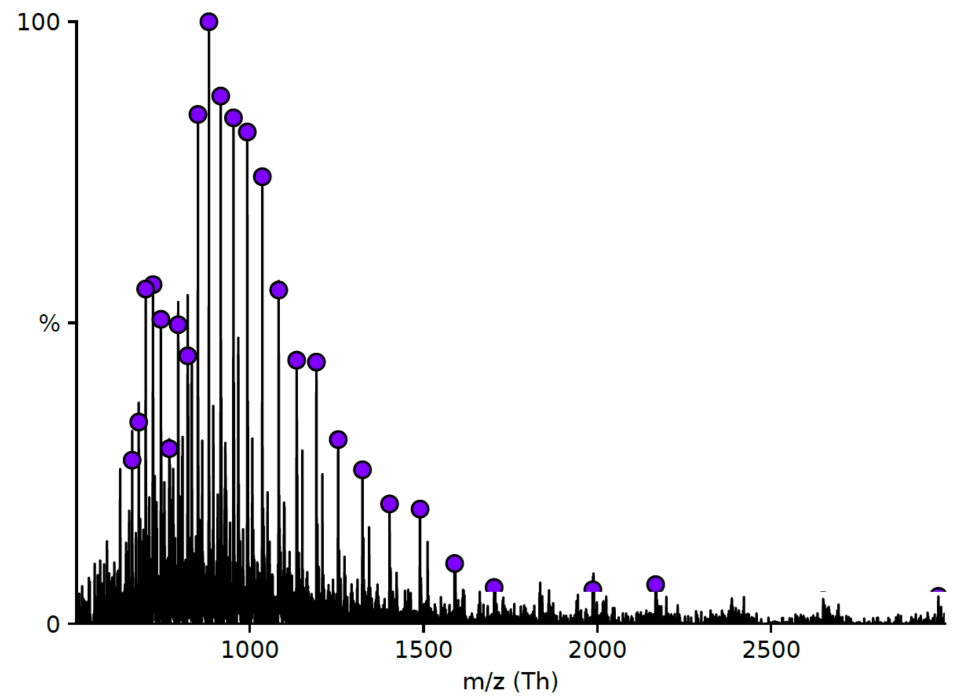

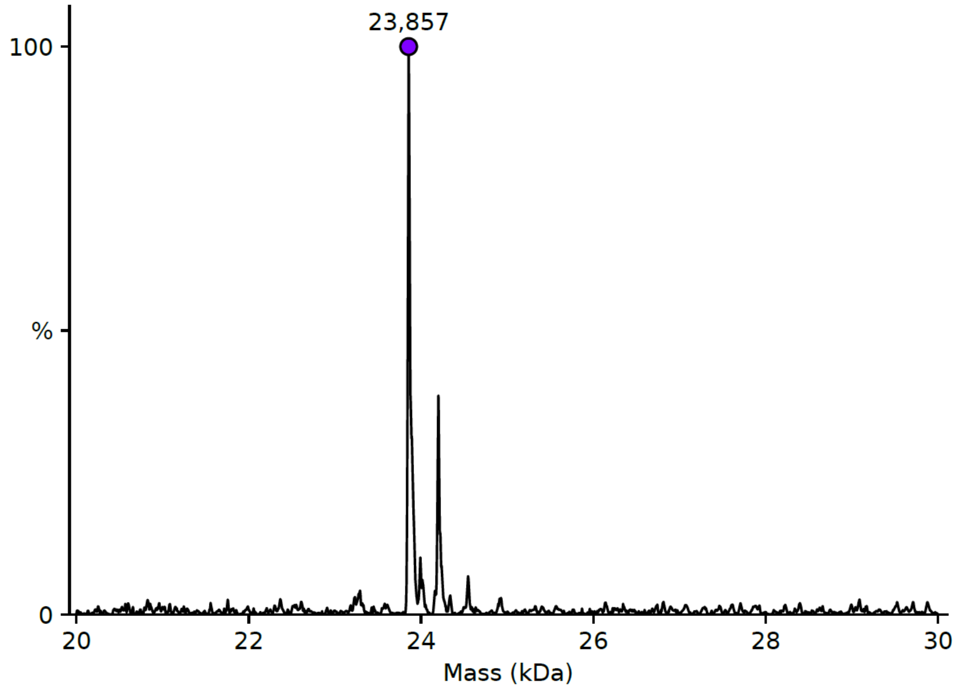

Figure S35: Mass spectra of **cap71RNA_U^{Et}**. a) raw spectrum. b) deconvoluted spectrum; calculated mass: 23854 Da, found mass: 23857 Da, Δ = 3 Da (**cap71RNA_U^{Et}**).

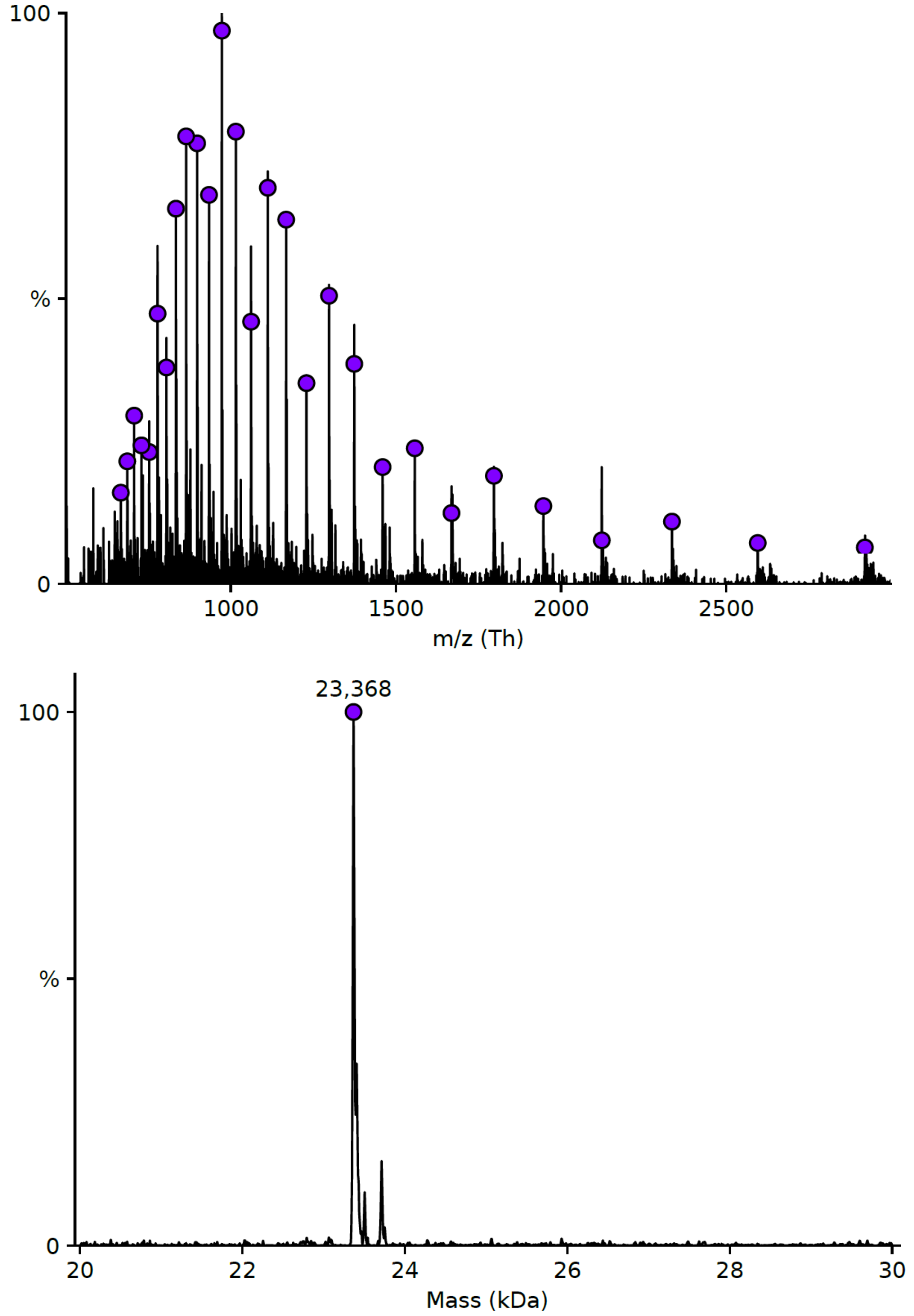

Figure S36: Mass spectra of **cap71RNA_A^{H}**. a) raw spectrum. b) deconvoluted spectrum; calculated mass: 23366 Da, found mass: 23368 Da, Δ = 2 Da (**cap71RNA_A^{H}**).

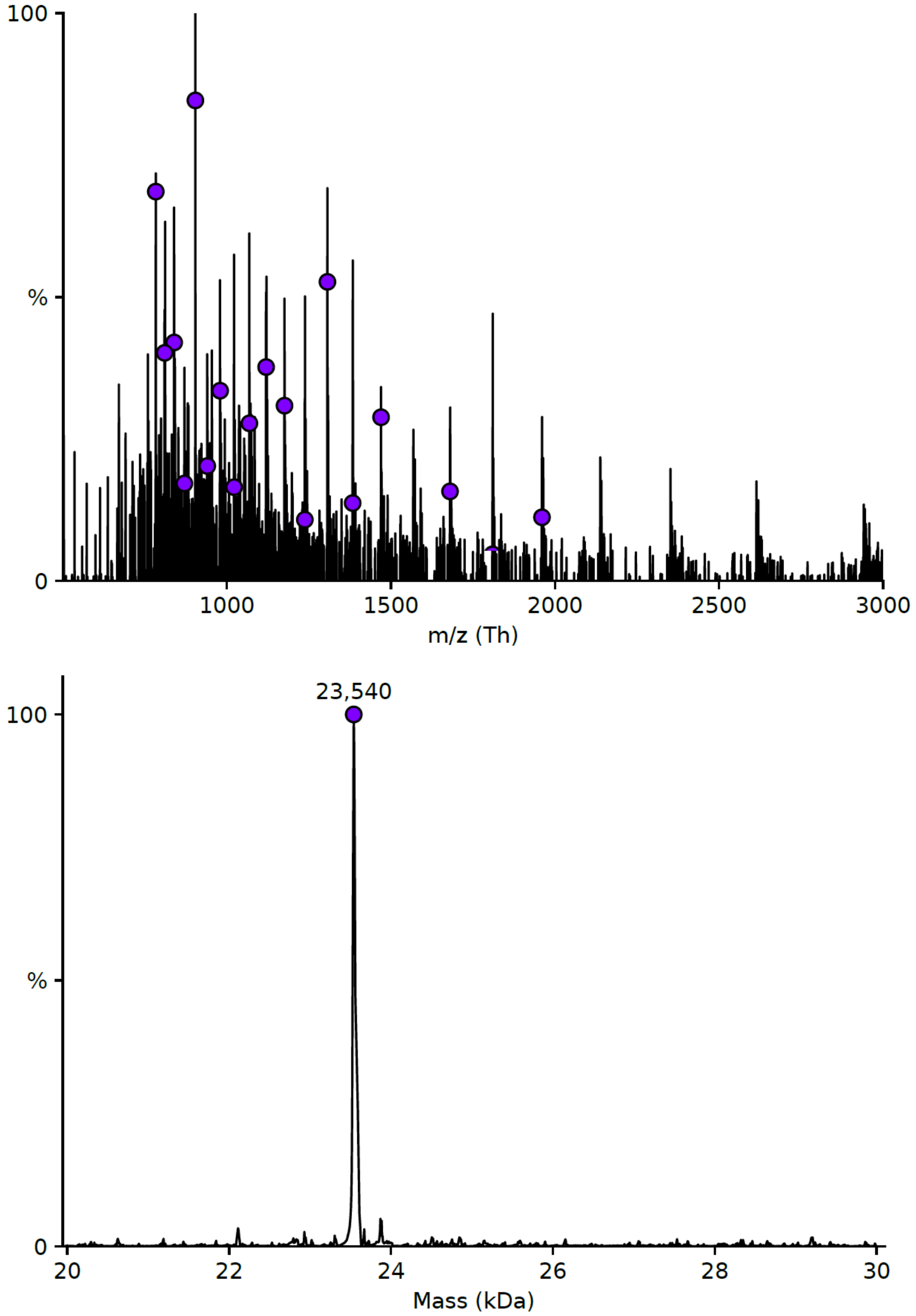

Figure S37: Mass spectra of **cap71RNA_A^{Me}**. a) raw spectrum. b) deconvoluted spectrum; calculated mass: 23546 Da, found mass: 23540 Da, Δ = 6 Da (**cap71RNA_A^{Me}**).

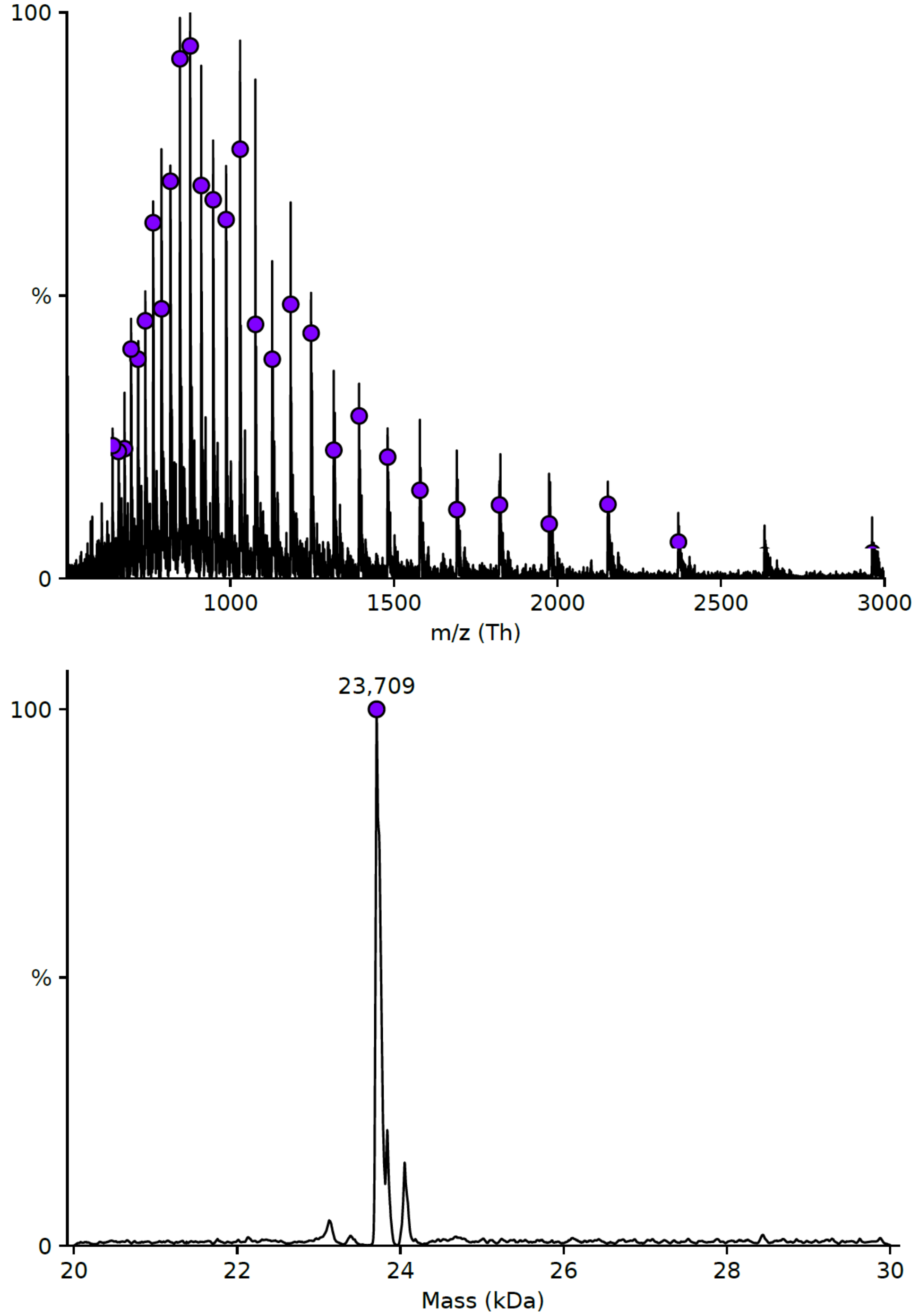

Figure S38: Mass spectra of **cap71RNA_A^{Et}**. a) raw spectrum. b) deconvoluted spectrum; calculated mass: 23714 Da, found mass: 23709 Da, Δ = 5 Da (**cap71RNA_A^{Et}**).

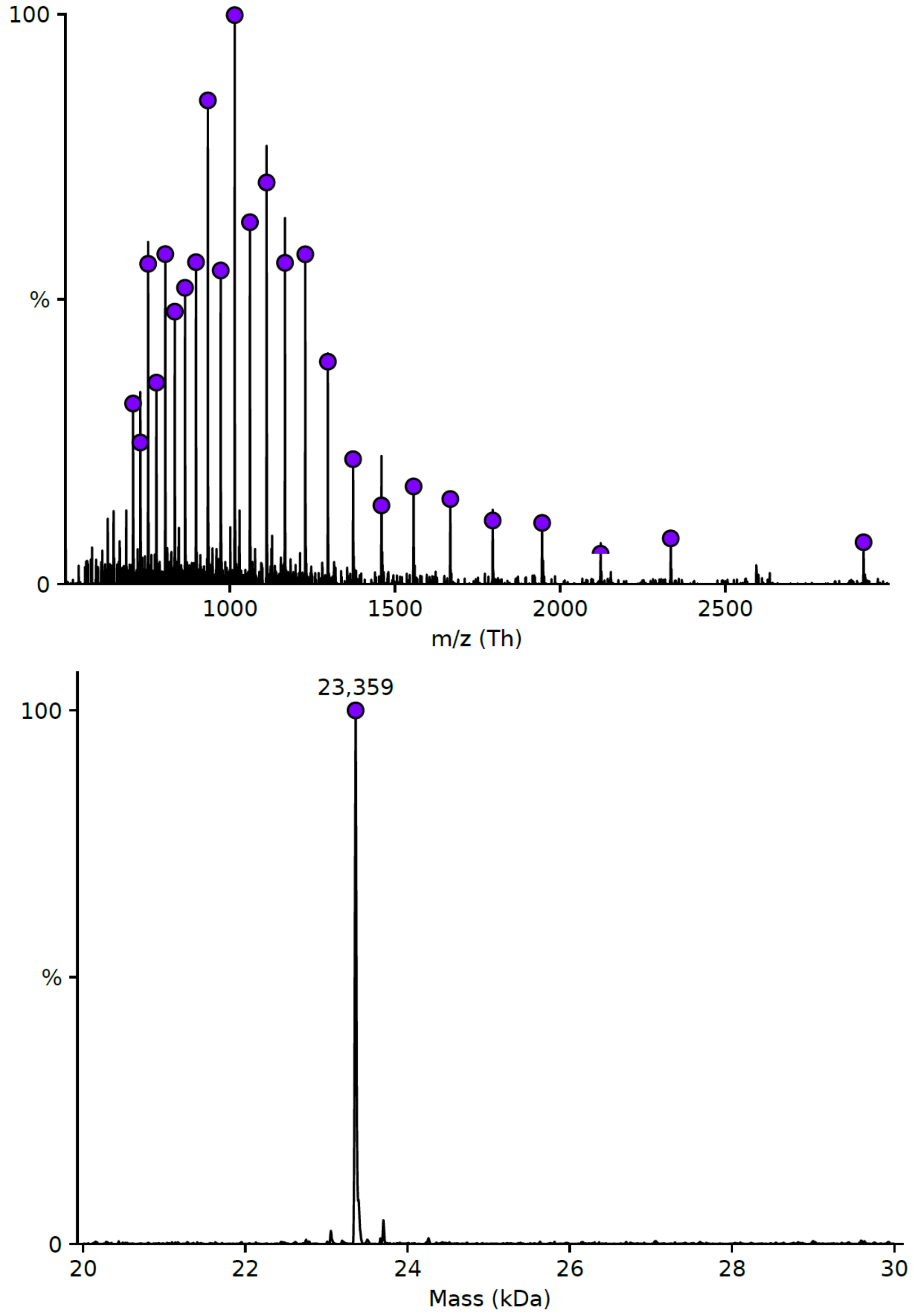

Figure S39: Mass spectra of **cap71RNA_G^{H}**. a) raw spectrum. b) deconvoluted spectrum; calculated mass: 23356 Da, found mass: 23359 Da, Δ = 3 Da (**cap71RNA_G^{H}**).

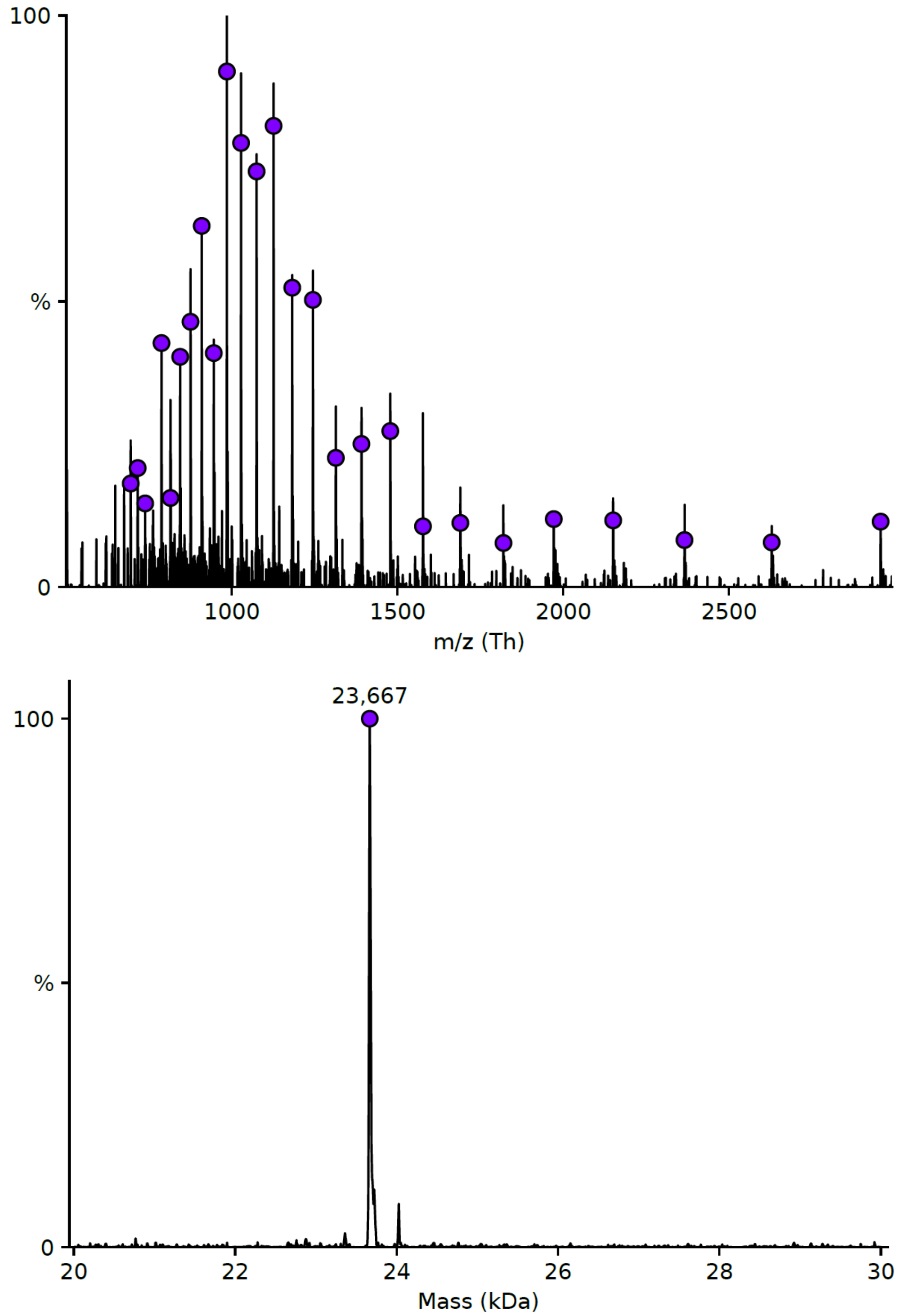

Figure S40: Mass spectra of **cap71RNA_G^{Me}**. a) raw spectrum. b) deconvoluted spectrum; calculated mass: 23686 Da, found mass: 23667 Da, Δ = 19 Da (**cap71RNA_G^{Me}**).

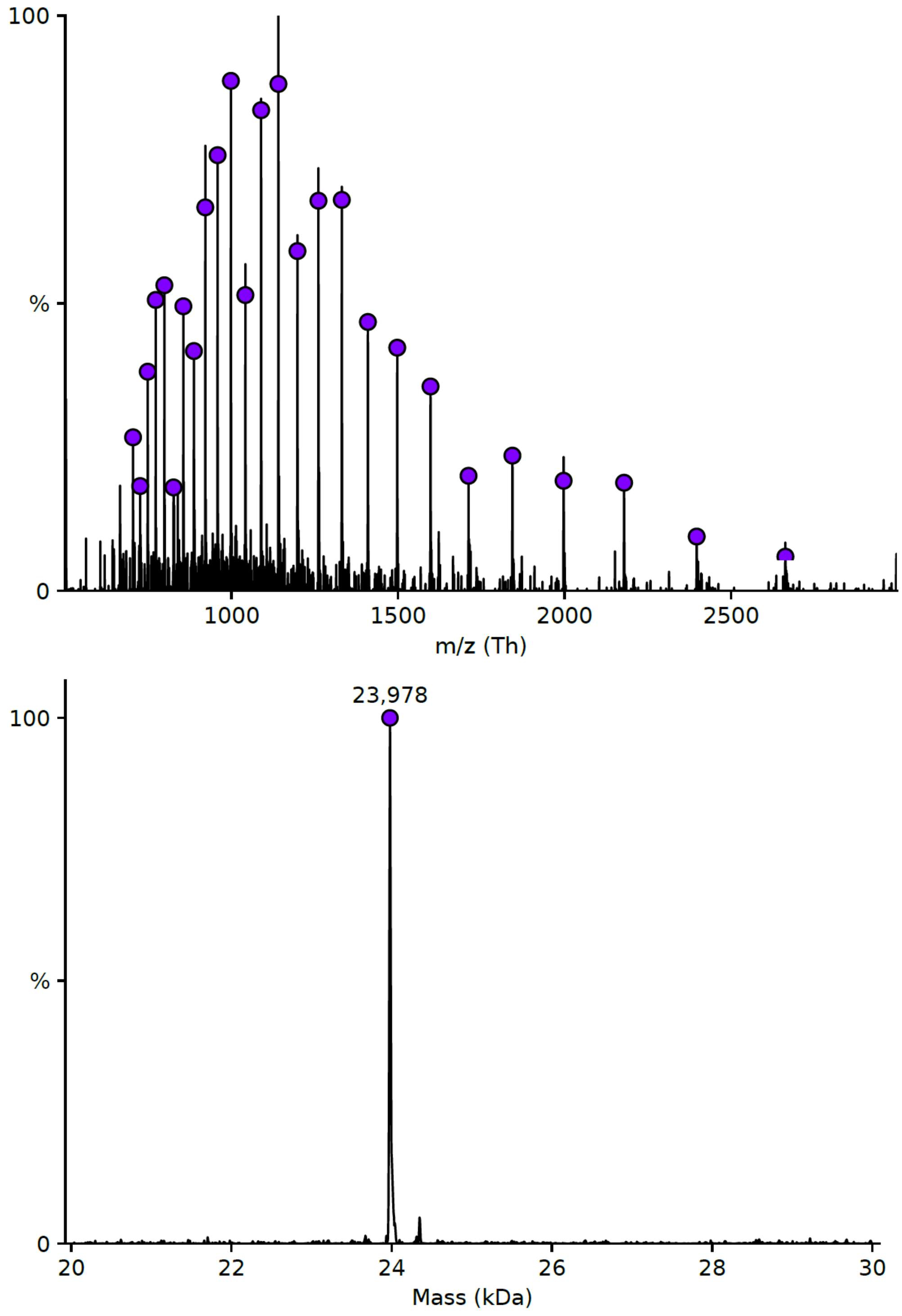

Figure S41: Mass spectra of **cap71RNA_G^{Et}**. a) raw spectrum. b) deconvoluted spectrum; calculated mass: 23994 Da, found mass: 23978 Da, Δ = 16 Da (**cap71RNA_G^{Et}**).

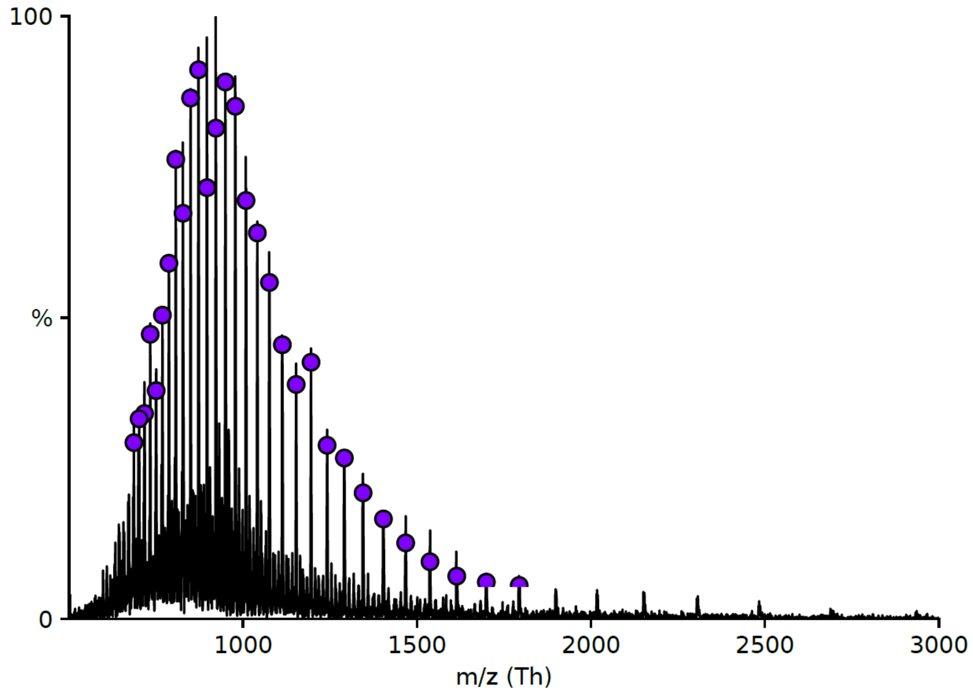

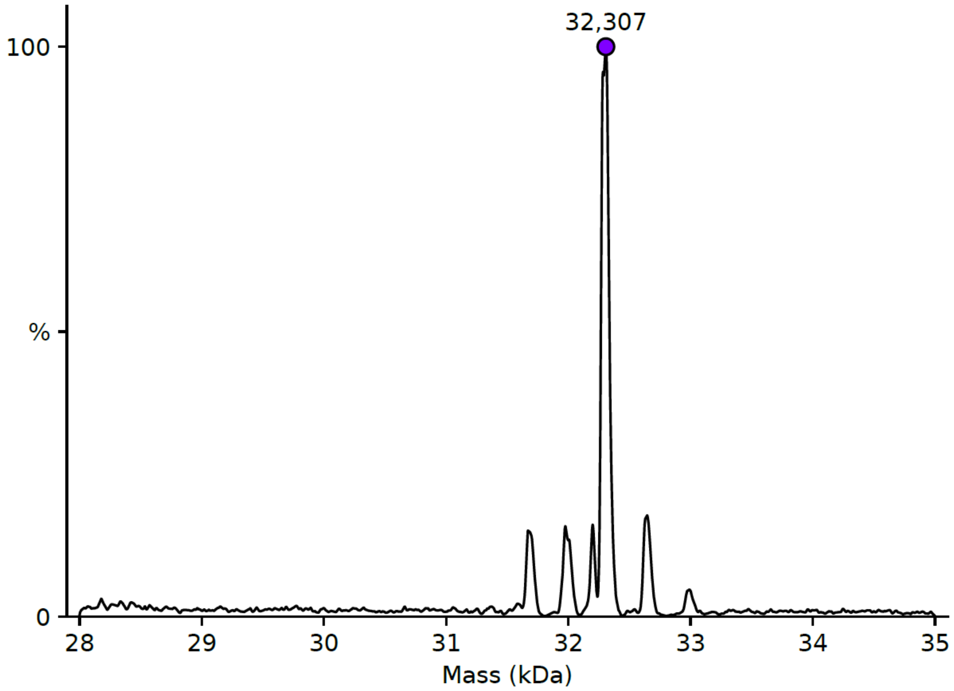

Figure S42: Mass spectra of **sgRNA_nat** a) raw spectrum. b) deconvoluted spectrum; calculated mass: 32280 Da, found mass: 31307 Da, Δ = 27 Da (**sgRNA_nat** + Na^+).

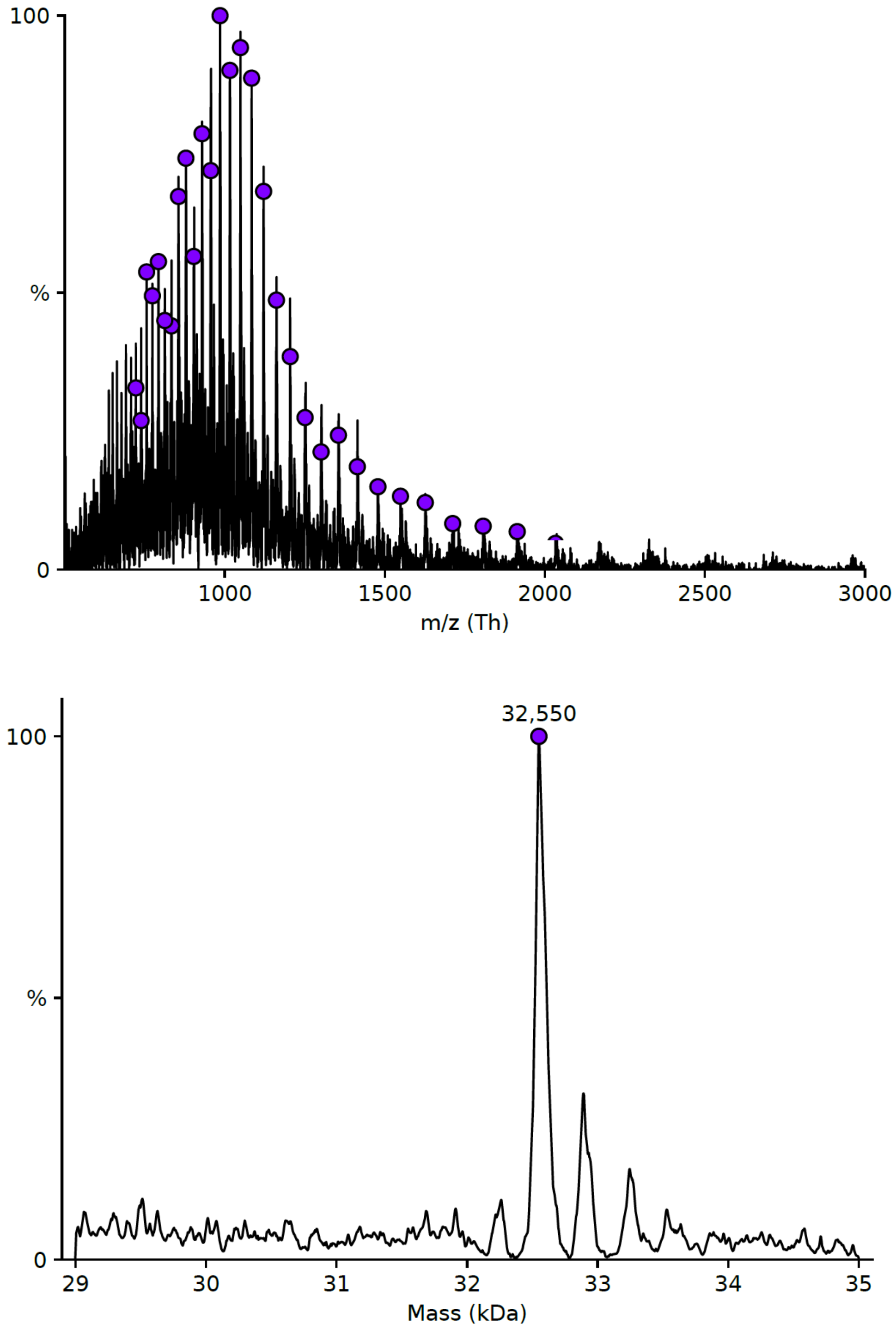

Figure S43: Mass spectra of **sgRNA_C^{Me}** a) raw spectrum. b) deconvoluted spectrum; calculated mass: 32504 Da, found mass: 32550 Da, Δ = 46 Da (**sgRNA_C^{Me}** + 2 Na^{+}).

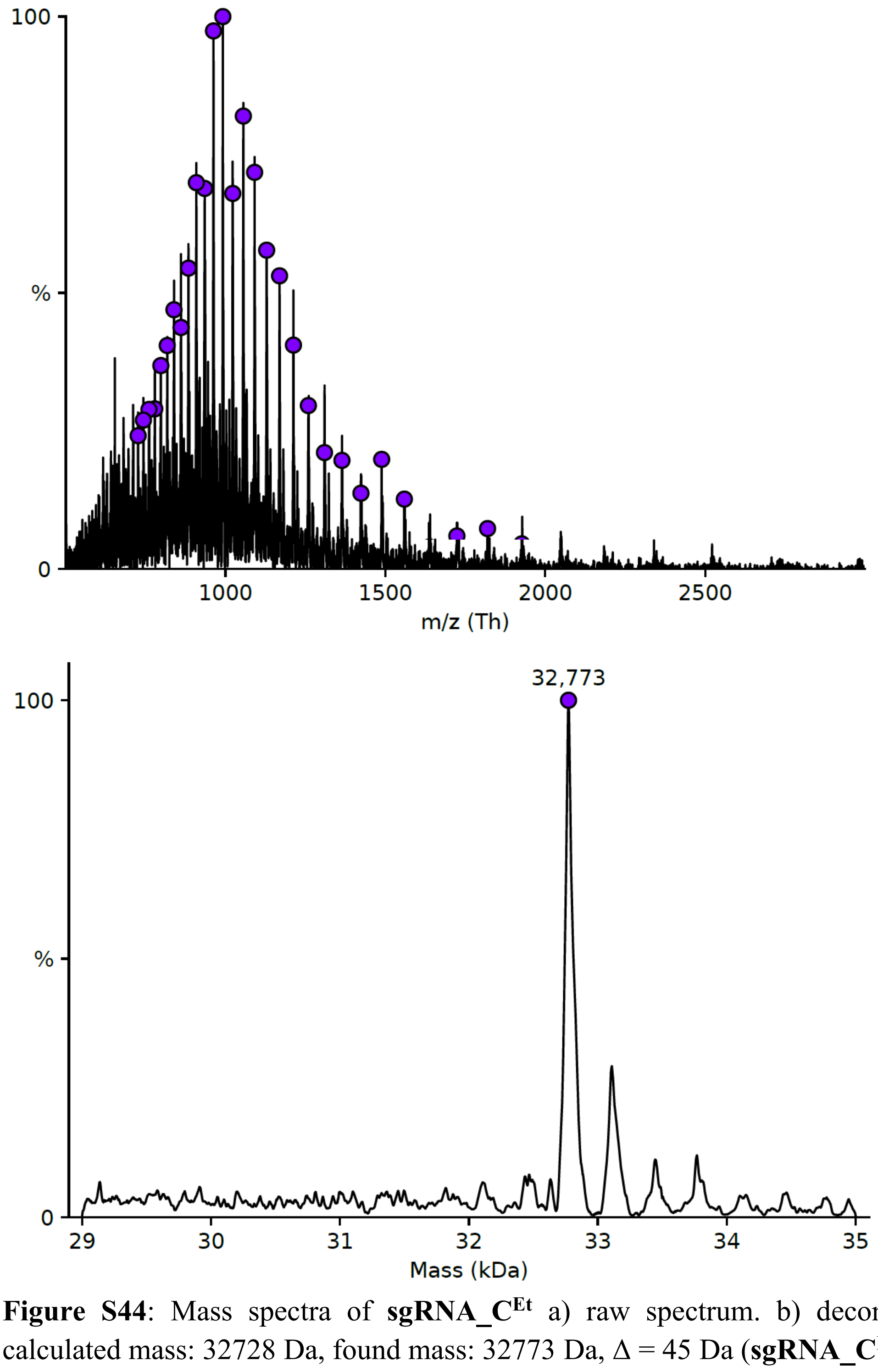

Figure S44: Mass spectra of **sgRNA_C^{Et}** a) raw spectrum. b) deconvoluted spectrum; calculated mass: 32728 Da, found mass: 32773 Da, Δ = 45 Da (**sgRNA_C^{Et}** + 2 Na^{+}).

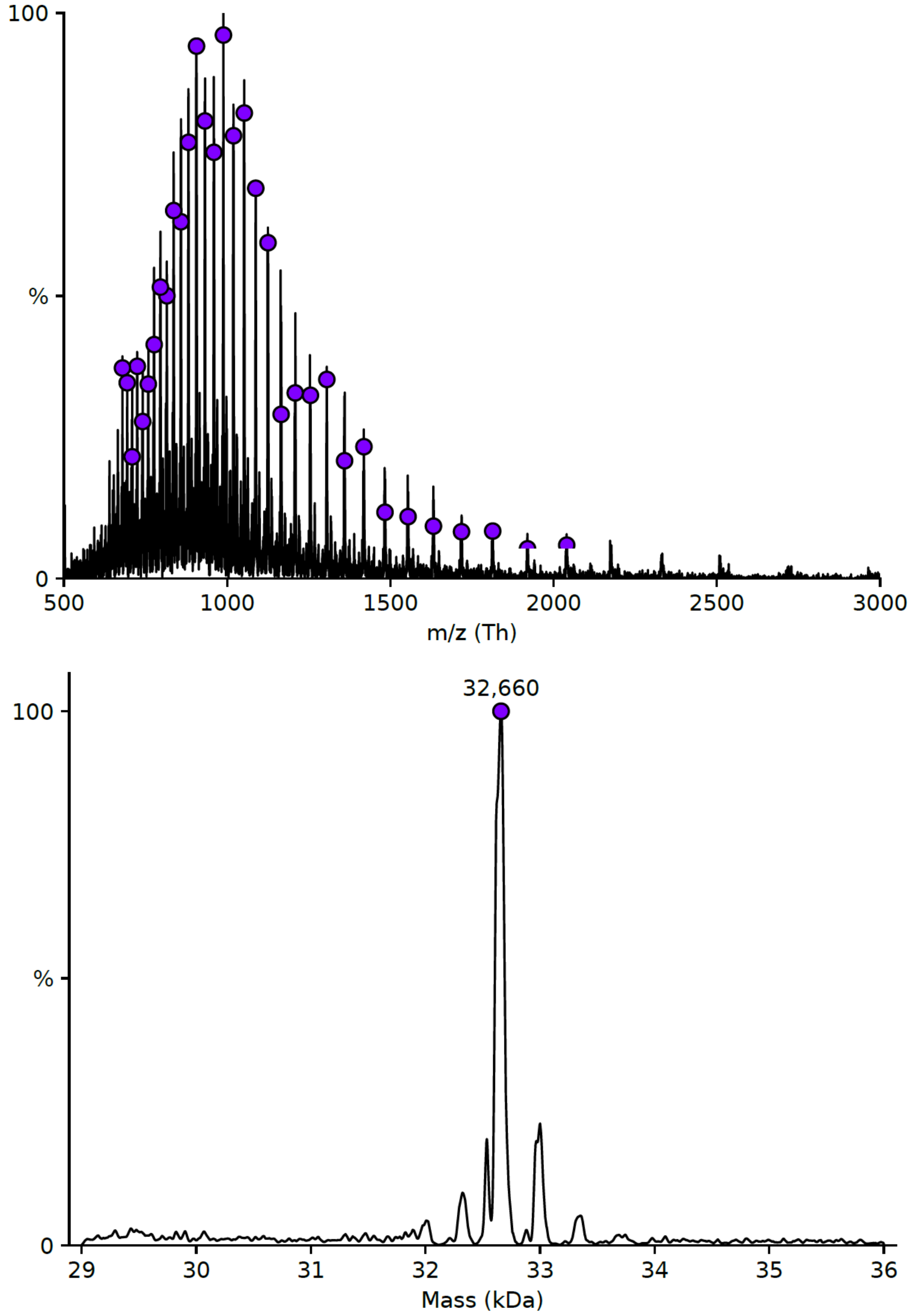

Figure S45: Mass spectra of **sgRNA_U^{Me}** a) raw spectrum. b) deconvoluted spectrum; calculated mass: 32616 Da, found mass: 32660 Da, Δ = 44 Da (**sgRNA_U^{Me}** + K^+).

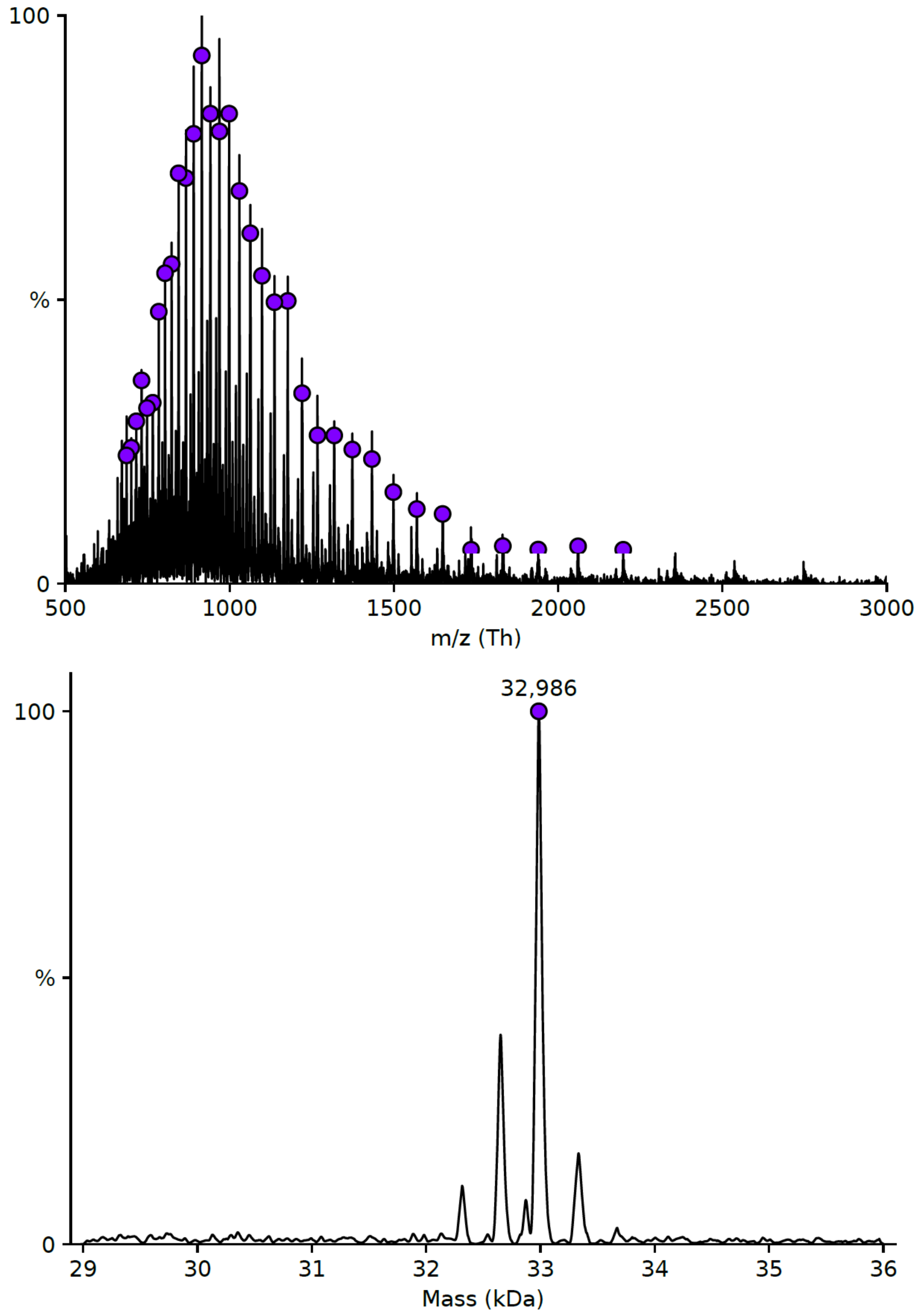

Figure S46: Mass spectra of **sgRNA_U^{Et}** a) raw spectrum. b) deconvoluted spectrum; calculated mass: 32952 Da, found mass: 32986 Da, Δ = 34 Da (**sgRNA_U^{Et}** + K^+).

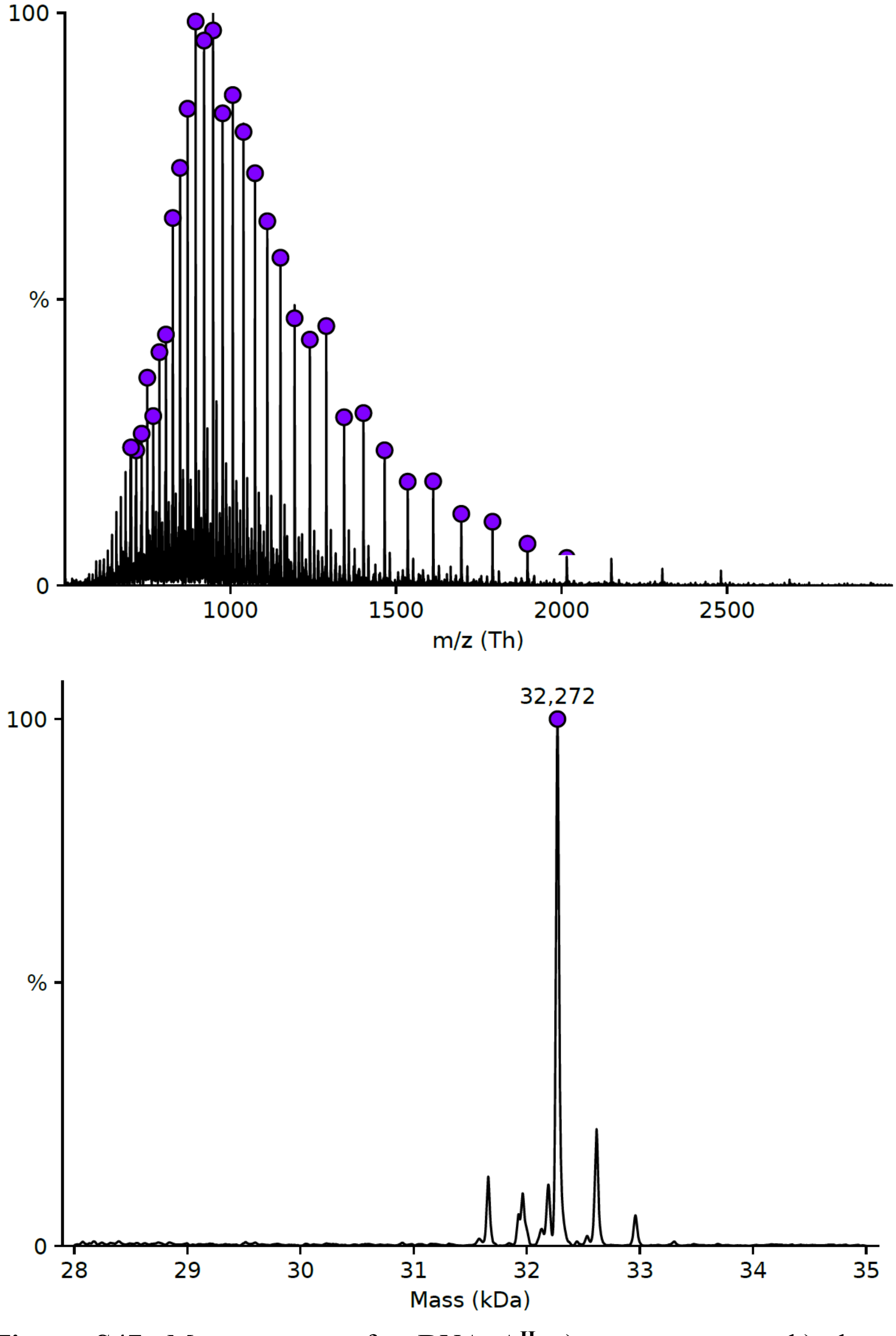

Figure S47: Mass spectra of **sgRNA_A^{H}** a) raw spectrum. b) deconvoluted spectrum; calculated mass: 32249 Da, found mass: 32272 Da, Δ = 35 Da (**sgRNA_A^{H}** + Na^{+}).

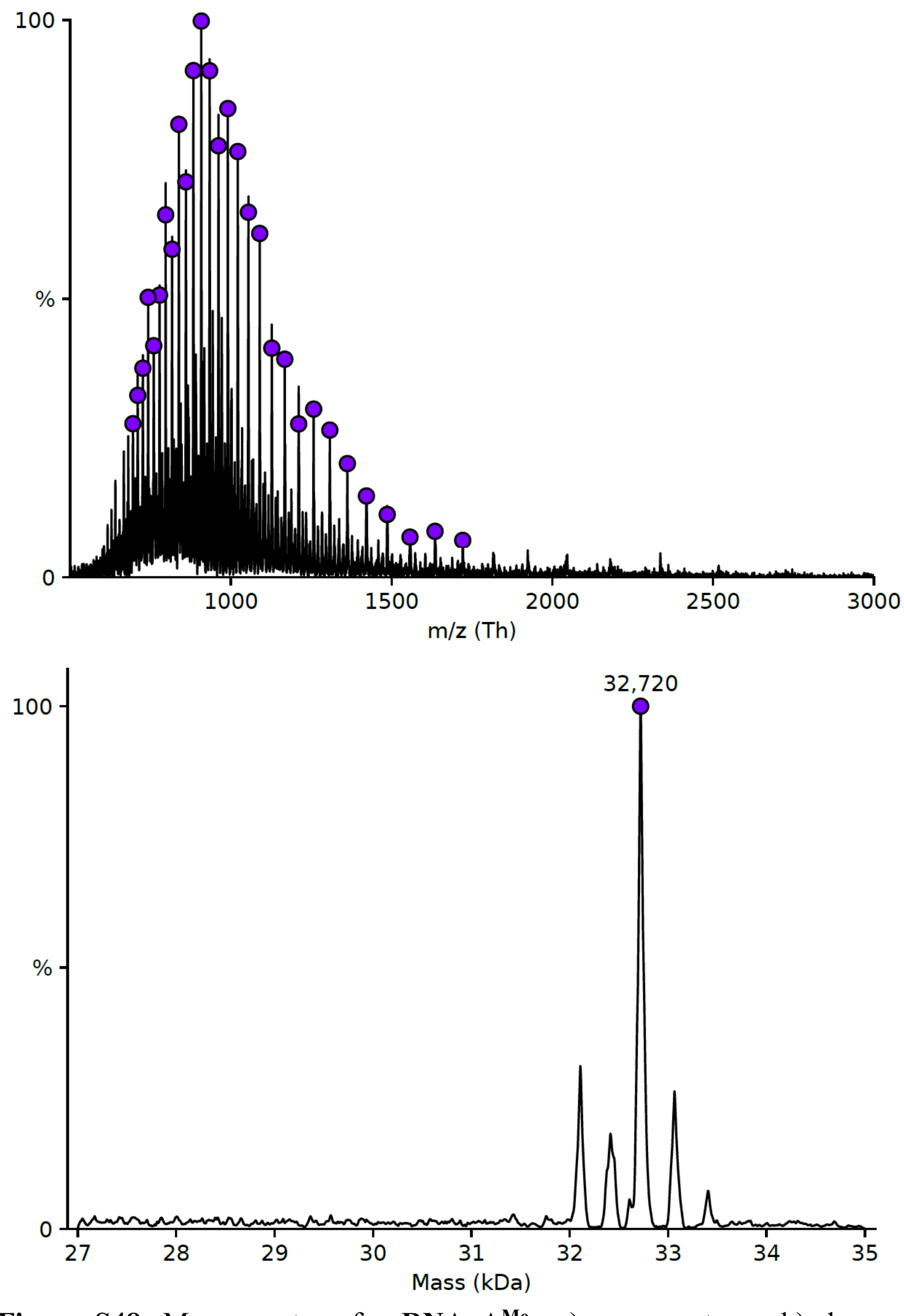

Figure S48: Mass spectra of **sgRNA_A^{Me}** a) raw spectrum. b) deconvoluted spectrum; calculated mass: 32714 Da, found mass: 32720 Da, Δ = 6 Da (**sgRNA_A^{Me}**).

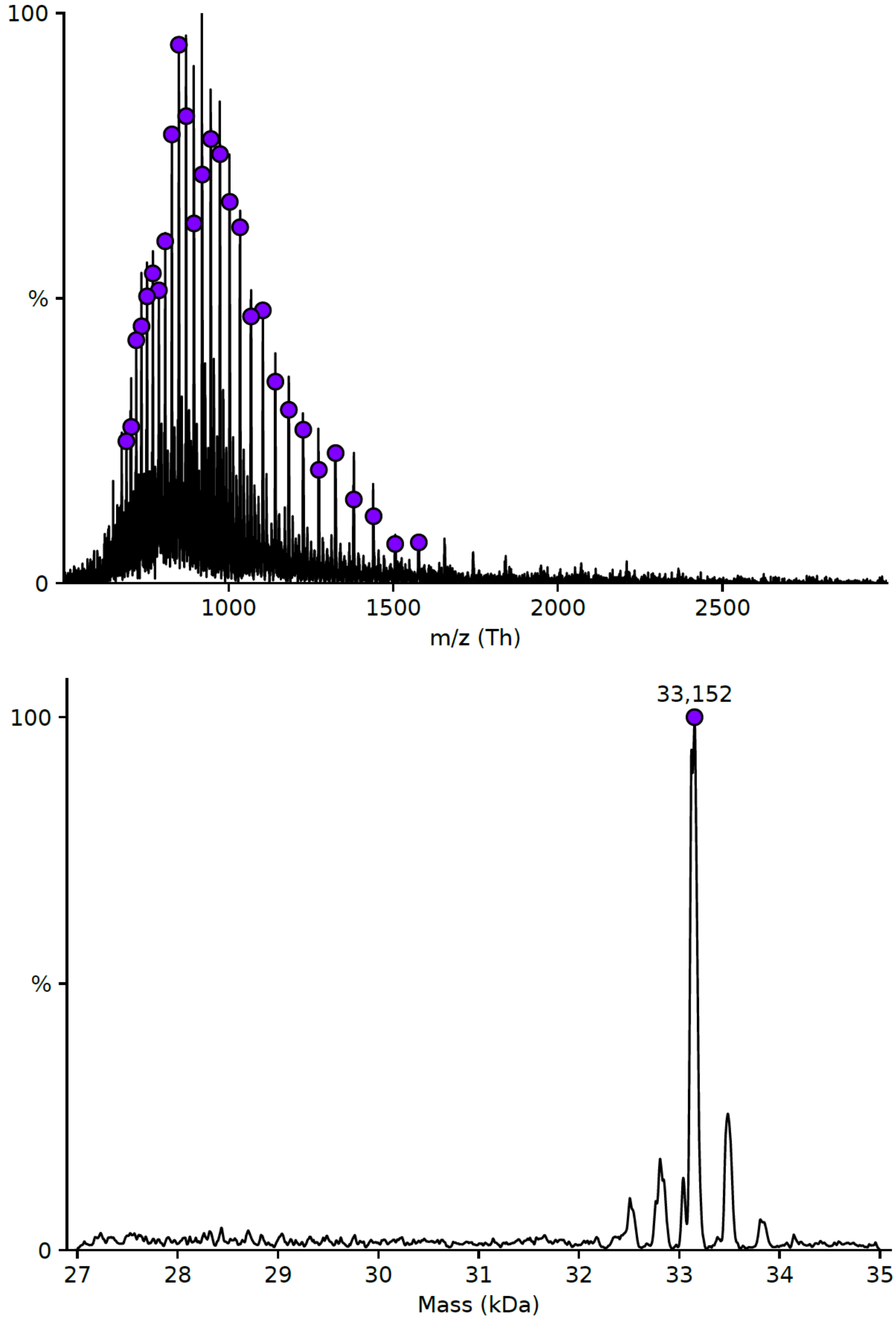

Figure S49: Mass spectra of **sgRNA_A^{Et}** a) raw spectrum. b) deconvoluted spectrum; calculated mass: 33148 Da, found mass: 33152 Da, Δ = 4 Da (**sgRNA_A^{Et}**).

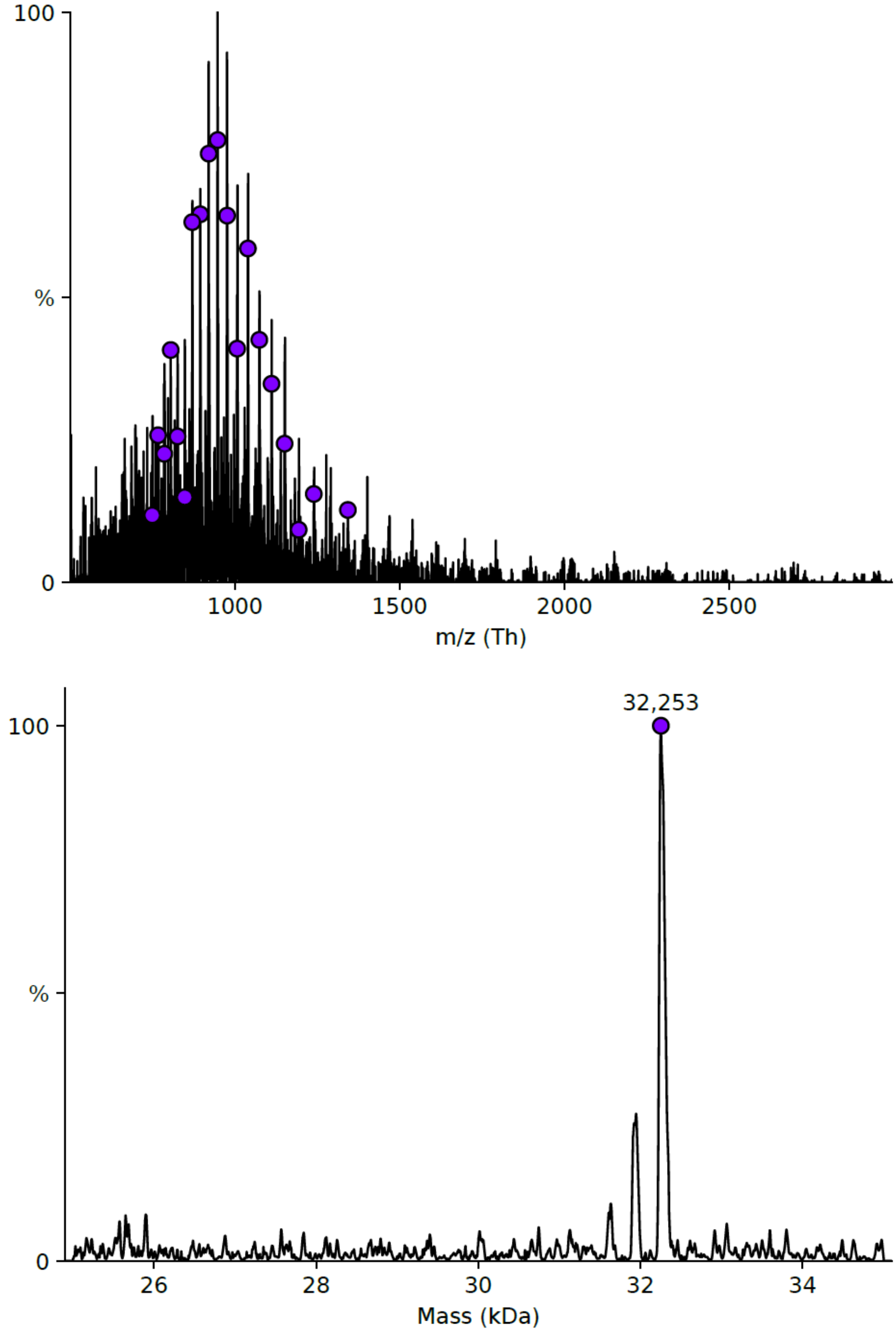

Figure S50: Mass spectra of **sgRNA_G^H^** a) raw spectrum. b) deconvoluted spectrum; calculated mass: 32252 Da, found mass: 32253 Da, Δ = 1 Da (**sgRNA_G^H^**)

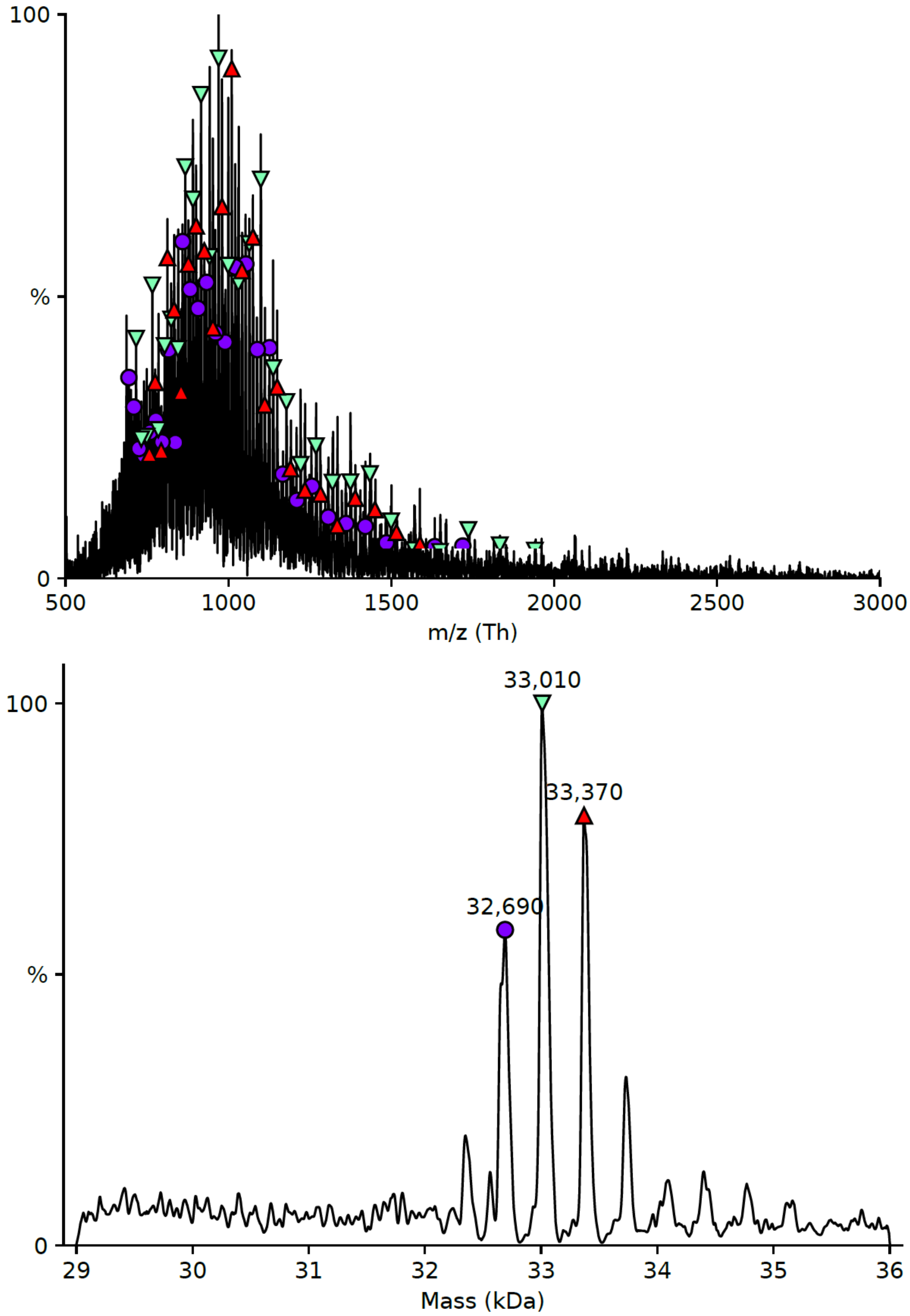

Figure S51: Mass spectra of **sgRNA_G^{Me}** a) raw spectrum. b) deconvoluted spectrum; calculated mass: 32644 Da, found mass: 32690 Da, Δ = 46 Da (**sgRNA_G^{Me}** + 2 Na^+); found mass: 33010 Da, Δ = 366 Da (**sgRNA_G^{Me}** + Na^+ + rGMP); found mass: 33370 Da, Δ = 726 Da (**sgRNA_G^{Me}** + K^+ + 2 rGMP).

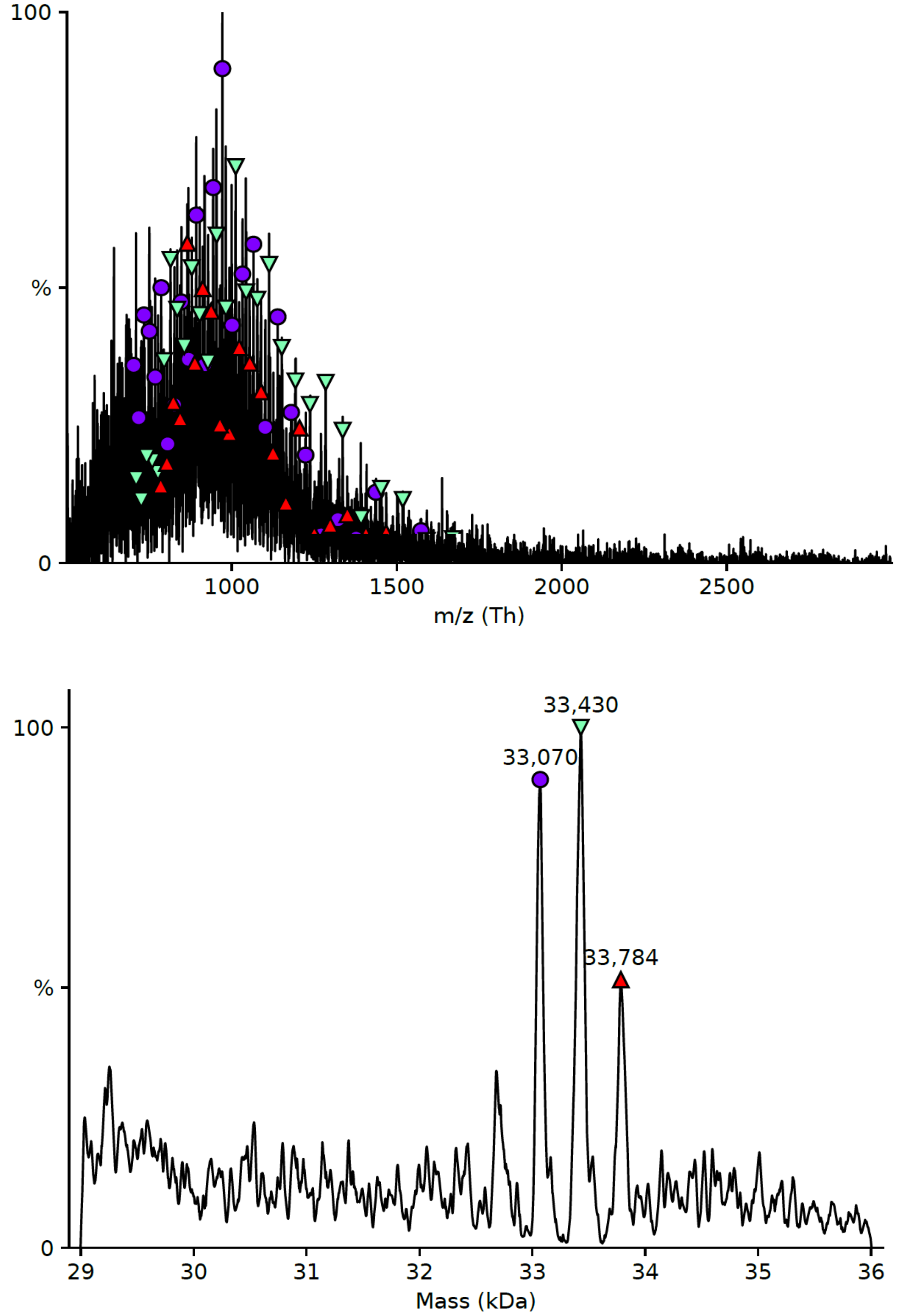

Figure S52: Mass spectra of **sgRNA_G^{Et}** a) raw spectrum. b) deconvoluted spectrum; calculated mass: 33036 Da, found mass: 33070 Da, Δ = 34 Da (**sgRNA_G^{Et}** + K^+); found mass: 33430 Da, Δ = 394 Da (**sgRNA_G^{Et}** + rGMP + K^+); found mass: 33784 Da, Δ = 748 Da (**sgRNA_G^{Et}** + 2 rGMP + K^+ + Na^+).

6 Appendix

6.1 Sequence of AAVS1

AAVS1 DNA amplicon (strand 1)

CTCCCTCCCAGGATCCTCTCTGGCTCCATCGTAAGCAAACCTTAGAGGTTCTGGC
AAGGAGAGAGATGGCTCCAGGAAATGGGGGTGTGTCACCAGATAAGGAATCTGC
CTAACAGGAGGTGGGGGTTAGACCCAATATCAGGAGACTAGGAAGGAGGAGGC
CTAAGGATGGGGCTTTTCTGTCACCAATCCTGTCCCTAGTGGCCCCACTGTGGG
GTGGAGGGGACAGATAAAAGTACCCAGAACCAGAGCCACATTAACCGGCCCTGG
GAATATAAGGTGGTCCCAGCTCGGGGACACAGGATCCCTGGAGGCAGCAAACAT
GCTGTCCTGAAGTGGACATAGGGGCCCGGGTTGGAGGAAGAAGACTAGCTGAGC
TCTCGGACCCCTGGAAGATGCCATGACAGGGGGCTGGAAGAGCTAGCACAGACT
AGAGAGGTAAGGGGGGTAGGGGAGCTGCCCAAATGAAAGGAGTGAGAGGTGAC
CCGAATCCACAGGAGAACGGGG

AAVS1 DNA amplicon (strand 2)

CCCCGTTCTCCTGTGGATTCGGGTCACCTCTCACTCCTTTCATTTGGGCAGCTCCC
CTACCCCCCTTACCTCTCTAGTCTGTGCTAGCTCTTCCAGCCCCCTGTCATGGCAT
CTTCCAGGGGTCCGAGAGCTCAGCTAGTCTTCTTCCTCCAACCCGGGCCCCTATG
TCCACTTCAGGACAGCATGTTTGCTGCCTCCAGGGATCCTGTGTCCCCGAGCTGG
GACCACCTTATATTCCCAGGGCCGGTTAATGTGGCTCTGGTTCTGGGTACTTTTAT
CTGTCCCCTCCACCCCACAGTGGGGCCACTAGGGACAGGATTGGTGACAGAAAA
GCCCCATCCTTAGGCCTCCTCCTTCCTAGTCTCCTGATATTGGGTCTAACCCCCAC
CTCCTGTTAGGCAGATTCCTTATCTGGTGACACACCCCCATTTCCTGGAGCCATCT
CTCTCCTTGCCAGAACCTCTAAGGTTTGCTTACGATGGAGCCAGAGAGGATCCTG
GGAGGGAG

In orange PAM sequence, in blue protospacer.

6.2 Sequences and map of plasmid

6.2.1 phRL-SV40

```
  1 agatctgcgc agcaccatgg cctgaaataa cctctgaaag aggaacttgg ttaggtacct
 61 tctgaggcgg aaagaaccag ctgtggaatg tgtgtcagtt agggtgtgga aagtccccag
121 gctccccagc aggcagaagt atgcaaagca tgcatctcaa ttagtcagca accaggtgtg
181 gaaagtcccc aggctcccca gcaggcagaa gtatgcaaag catgcatctc aattagtcag
241 caaccatagt cccgccccta actccgccca tcccgcccct aactccgccc agttccgccc
301 attctccgcc ccatggctga ctaatttttt ttatttatgc agaggccgag gccgcctcgg
361 cctctgagct attccagaag tagtgaggag gcttttttgg aggcctaggc ttttgcaaaa
421 agcttgattc ttctgacaca acagtctcga acttaagctg cagaagttgg tcgtgaggca
481 ctgggcaggt aagtatcaag gttacaagac aggtttaagg agaccaatag aaactgggct
541 tgtcgagaca gagaagactc ttgcgtttct gataggcacc tattggtctt actgacatcc
601 actttgcctt tctctccaca ggtgtccact cccagttcaa ttacagctct taaggctaga
```

661 gtacttaata cgactcacta taggctagcc accatggctt ccaaggtgta cgaccccgag
721 caacgcaaac gcatgatcac tgggcctcag tggtgggctc gctgcaagca aatgaacgtg
781 ctggactcct tcatcaacta ctatgattcc gagaagcacg ccgagaacgc cgtgattttt
841 ctgcatggta acgctgcctc cagctacctg tggaggcacg tcgtgcctca catcgagccc
901 gtggctagat gcatcatccc tgatctgatc ggaatgggta agtccggcaa gagcgggaat
961 ggctcatatc gcctcctgga tcactacaag tacctcaccg cttggttcga gctgctgaac
1021 cttccaaaga aaatcatctt tgtgggccac gactgggggg cttgtctggc ctttcactac
1081 tcctacgagc accaagacaa gatcaaggcc atcgtccatg ctgagagtgt cgtggacgtg
1141 atcgagtcct gggacgagtg gcctgacatc gaggaggata tcgccctgat caagagcgaa
1201 gagggcgaga aaatggtgct tgagaataac ttcttcgtcg agaccatgct cccaagcaag
1261 atcatgcgga aactggagcc tgaggagttc gctgcctacc tggagccatt caaggagaag
1321 ggcgaggtta gacggcctac cctctcctgg cctcgcgaga tccctctcgt taagggaggc
1381 aagcccgacg tcgtccagat tgtccgcaac tacaacgcct accttcgggc cagcgacgat
1441 ctgcctaaga tgttcatcga gtccgaccct gggttctttt ccaacgctat tgtcgaggga
1501 gctaagaagt tccctaacac cgagttcgtg aaggtgaagg gcctccactt cagccaggag
1561 gacgctccag atgaaatggg taagtacatc aagagcttcg tggagcgcgt gctgaagaac
1621 gagcagtaat tctagagcgg ccgcttcgag cagacatgat aagatacatt gatgagtttg
1681 gacaaaccac aactagaatg cagtgaaaaa aatgctttat ttgtgaaatt tgtgatgcta
1741 ttgctttatt tgtaaccatt ataagctgca ataaacaagt taacaacaac aattgcattc
1801 attttatgtt tcaggttcag ggggaggtgt gggaggtttt ttaaagcaag taaaacctct
1861 acaaatgtgg taaaatcgat aaggatccag gtggcacttt tcggggaaat gtgcgcggaa
1921 cccctatttg tttatttttc taaatacatt caaatatgta tccgctcatg agacaataac
1981 cctgataaat gcttcaataa tattgaaaaa ggaagagtat gagtattcaa catttccgtg
2041 tcgcccttat tccctttttt gcggcatttt gccttcctgt ttttgctcac ccagaaacgc
2101 tggtgaaagt aaaagatgct gaagatcagt tgggtgcacg agtgggttac atcgaactgg
2161 atctcaacag cggtaagatc cttgagagtt ttcgccccga agaacgtttt ccaatgatga
2221 gcacttttaa agttctgcta tgtggcgcgg tattatcccg tattgacgcc gggcaagagc
2281 aactcggtcg ccgcatacac tattctcaga atgacttggt tgagtactca ccagtcacag
2341 aaaagcatct tacggatggc atgacagtaa gagaattatg cagtgctgcc ataaccatga
2401 gtgataacac tgcggccaac ttacttctga caacgatcgg aggaccgaag gagctaaccg
2461 cttttttgca caacatgggg gatcatgtaa ctcgccttga tcgttgggaa ccggagctga
2521 atgaagccat accaaacgac gagcgtgaca ccacgatgcc tgtagcaatg gcaacaacgt
2581 tgcgcaaact attaactggc gaactactta ctctagcttc ccggcaacaa ttaatagact
2641 ggatggaggc ggataaagtt gcaggaccac ttctgcgctc ggcccttccg gctggctggt
2701 ttattgctga taaatctgga gccggtgagc gtgggtctcg cggtatcatt gcagcactgg
2761 ggccagatgg taagccctcc cgtatcgtag ttatctacac gacggggagt caggcaacta
2821 tggatgaacg aaatagacag atcgctgaga taggtgcctc actgattaag cattggtaac
2881 tgtcagacca agtttactca tatatacttt agattgattt aaaacttcat ttttaattta
2941 aaaggatcta ggtgaagatc ctttttgata atctcatgac caaaatccct taacgtgagt
3001 tttcgttcca ctgagcgtca gaccccgtag aaaagatcaa aggatcttct tgagatcctt
3061 tttttctgcg cgtaatctgc tgcttgcaaa caaaaaaacc accgctacca gcggtggttt
3121 gtttgccgga tcaagagcta ccaactcttt ttccgaaggt aactggcttc agcagagcgc
3181 agataccaaa tactgttctt ctagtgtagc cgtagttagg ccaccacttc aagaactctg
3241 tagcaccgcc tacatacctc gctctgctaa tcctgttacc agtggctgct gccagtggcg
3301 ataagtcgtg tcttaccggg ttggactcaa gacgatagtt accggataag gcgcagcggt
3361 cgggctgaac ggggggttcg tgcacacagc ccagcttgga gcgaacgacc tacaccgaac
3421 tgagatacct acagcgtgag ctatgagaaa gcgccacgct tcccgaaggg agaaaggcgg
3481 acaggtatcc ggtaagcggc agggtcggaa caggagagcg cacgagggag cttccagggg

3541 gaaacgcctg gtatctttat agtcctgtcg ggtttcgcca cctctgactt gagcgtcgat
3601 ttttgtgatg ctcgtcaggg gggcggagcc tatggaaaaa cgccagcaac gcggcctttt
3661 tacggttcct ggccttttgc tggccttttg ctcacatggc tcgac

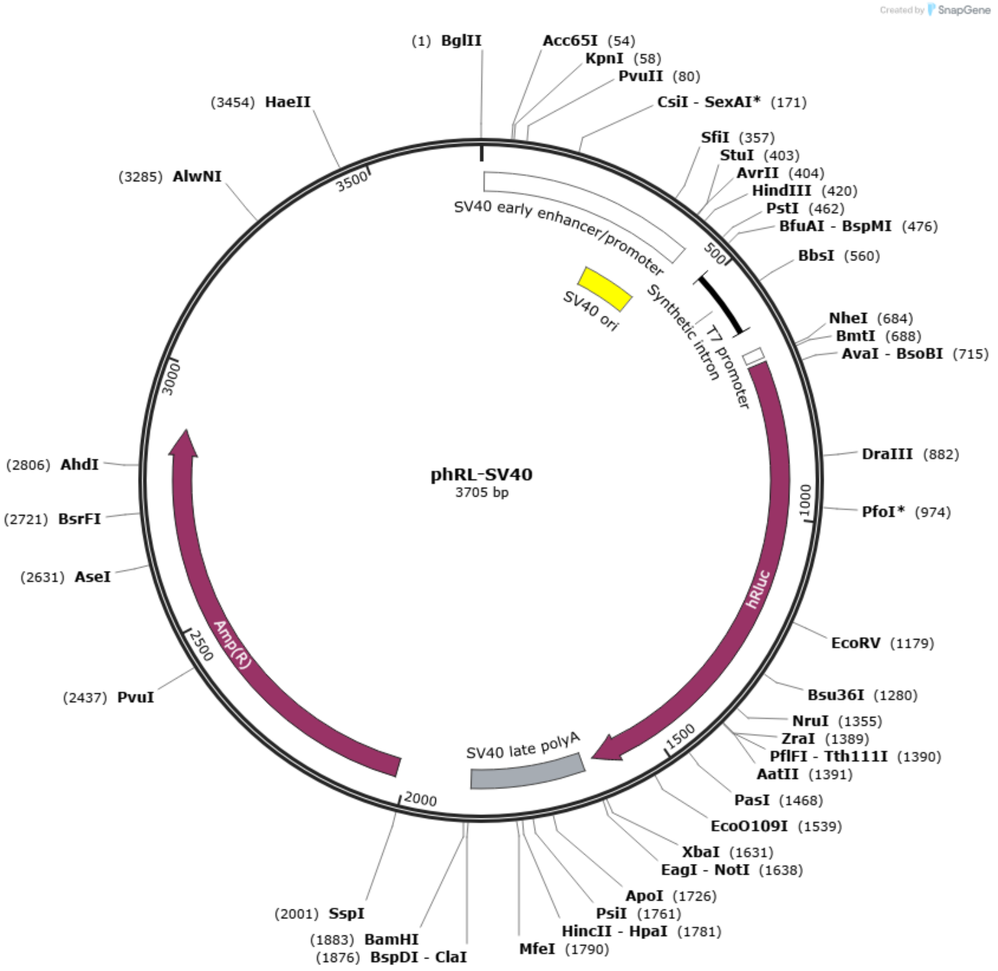

6.2.2 pGL4-CMV

1 ggcctaactg gccggtacct gagctcgcta gcctcgagga tatcaagatc tcccgatccc
61 ctatggtgca ctctcagtac aatctgctct gatgccgcat agttaagcca gtatctgctc
121 cctgcttgtg tgttggaggt cgctgagtag tgcgcgagca aaatttaagc tacaacaagg
181 caaggcttga ccgacaattg catgaagaat ctgcttaggg ttaggcgttt tgcgctgctt
241 cgcgatgtac gggccagata tacgcgttga cattgattat tgactagtta ttaatagtaa
301 tcaattacgg ggtcattagt tcatagccca tatatggagt tccgcgttac ataacttacg
361 gtaaatggcc cgcctggctg accgcccaac gacccccgcc cattgacgtc aataatgacg
421 tatgttccca tagtaacgcc aatagggact ttccattgac gtcaatgggt ggagtattta
481 cggtaaactg cccacttggc agtacatcaa gtgtatcata tgccaagtac gccccctatt
541 gacgtcaatg acggtaaatg gcccgcctgg cattatgccc agtacatgac cttatgggac
601 tttcctactt ggcagtacat ctacgtatta gtcatcgcta ttaccatggt gatgcggttt
661 tggcagtaca tcaatgggcg tggatagcgg tttgactcac ggggatttcc aagtctccac
721 cccattgacg tcaatgggag tttgttttgg caccaaaatc aacgggactt tccaaaatgt
781 cgtaacaact ccgccccatt gacgcaaatg ggcggtaggc gtgtacggtg ggaggtctat

841 ataagcagag ctctctggct aactagagaa cccactgctt actggcttat cgaaattaat
901 acgactcact atagggagac ccaagctggc tagcgtttaa acgggccctc tagactcgag
961 cggccgccac tgtgctggat atctgcagaa ttccaccaca ctggactagt ggatccgagc
1021 tcggtaccaa gcttggcaat ccggtactgt tggtaaagcc accatggaag atgccaaaaa
1081 cattaagaag ggcccagcgc cattctaccc actcgaagac gggaccgccg gcgagcagct
1141 gcacaaagcc atgaagcgct acgccctggt gcccggcacc atcgccttta ccgacgcaca
1201 tatcgaggtg gacattacct acgccgagta cttcgagatg agcgttcggc tggcagaagc
1261 tatgaagcgc tatgggctga atacaaacca tcggatcgtg gtgtgcagcg agaatagctt
1321 gcagttcttc atgcccgtgt tgggtgccct gttcatcggt gtggctgtgg ccccagctaa
1381 cgacatctac aacgagcgcg agctgctgaa cagcatgggc atcagccagc ccaccgtcgt
1441 attcgtgagc aagaaagggc tgcaaaagat cctcaacgtg caaaagaagc taccgatcat
1501 acaaaagatc atcatcatgg atagcaagac cgactaccag ggcttccaaa gcatgtacac
1561 cttcgtgact tcccatttgc cacccggctt caacgagtac gacttcgtgc ccgagagctt
1621 cgaccgggac aaaaccatcg ccctgatcat gaacagtagt ggcagtaccg gattgcccaa
1681 gggcgtagcc ctaccgcacc gcaccgcttg tgtccgattc agtcatgccc gcgaccccat
1741 cttcggcaac cagatcatcc ccgacaccgc tatcctcagc gtggtgccat ttcaccacgg
1801 cttcggcatg ttcaccacgc tgggctactt gatctgcggc tttcgggtcg tgctcatgta
1861 ccgcttcgag gaggagctat tcttgcgcag cttgcaagac tataagattc aatctgccct
1921 gctggtgccc acactattta gcttcttcgc taagagcact ctcatcgaca agtacgacct
1981 aagcaacttg cacgagatcg ccagcggcgg ggcgccgctc agcaaggagg taggtgaggc
2041 cgtggccaaa cgcttccacc taccaggcat ccgccagggc tacggcctga cagaaacaac
2101 cagcgccatt ctgatcaccc ccgaagggga cgacaagcct ggcgcagtag gcaaggtggt
2161 gcccttcttc gaggctaagg tggtggactt ggacaccggt aagacactgg gtgtgaacca
2221 gcgcggcgag ctgtgcgtcc gtggccccat gatcatgagc ggctacgtta acaaccccga
2281 ggctacaaac gctctcatcg acaaggacgg ctggctgcac agcggcgaca tcgcctactg
2341 ggacgaggac gagcacttct tcatcgtgga ccggctgaag agcctgatca aatacaaggg
2401 ctaccaggta gccccagccg aactggagag catcctgctg caacacccca acatcttcga
2461 cgccggggtc gccggcctgc ccgacgacga tgccggcgag ctgcccgccg cagtcgtcgt
2521 gctggaacac ggtaaaacca tgaccgagaa ggagatcgtg gactatgtgg ccagccaggt
2581 tacaaccgcc aagaagctgc gcggtggtgt tgtgttcgtg gacgaggtgc ctaaaggact
2641 gaccggcaag ttggacgccc gcaagatccg cgagattctc attaaggcca agaagggcgg
2701 caagatcgcc gtgtaataat tctagagtcg ggcggccggc ccgcttcgag cagacatgat
2761 aagatacatt gatgagtttg gacaaaccac aactagaatg cagtgaaaaa aatgctttat
2821 ttgtgaaatt tgtgatgcta ttgctttatt tgtaaccatt ataagctgca ataaacaagt
2881 taacaacaac aattgcattc attttatgtt tcaggttcag ggggaggtgt gggaggtttt
2941 ttaaagcaag taaaacctct acaaatgtgg taaaatcgat aaggatccgt cgaccgatgc
3001 ccttgagagc cttcaaccca gtcagctcct tccggtgggc gcggggcatg actatcgtcg
3061 ccgcacttat gactgtcttc tttatcatgc aactcgtagg acaggtgccg gcagcgctct
3121 tccgcttcct cgctcactga ctcgctgcgc tcggtcgttc ggctgcggcg agcggtatca
3181 gctcactcaa aggcggtaat acggttatcc acagaatcag gggataacgc aggaaagaac
3241 atgtgagcaa aaggccagca aaaggccagg aaccgtaaaa aggccgcgtt gctggcgttt
3301 ttccataggc tccgcccccc tgacgagcat cacaaaaatc gacgctcaag tcagaggtgg
3361 cgaaacccga caggactata aagataccag gcgtttcccc ctggaagctc cctcgtgcgc
3421 tctcctgttc cgaccctgcc gcttaccgga tacctgtccg cctttctccc ttcgggaagc
3481 gtggcgcttt ctcatagctc acgctgtagg tatctcagtt cggtgtaggt cgttcgctcc
3541 aagctgggct gtgtgcacga accccccgtt cagcccgacc gctgcgcctt atccggtaac
3601 tatcgtcttg agtccaaccc ggtaagacac gacttatcgc cactggcagc agccactggt
3661 aacaggatta gcagagcgag gtatgtaggc ggtgctacag agttcttgaa gtggtggcct

3721 aactacggct acactagaag aacagtattt ggtatctgcg ctctgctgaa gccagttacc
3781 ttcggaaaaa gagttggtag ctcttgatcc ggcaaacaaa ccaccgctgg tagcggtggt
3841 ttttttgttt gcaagcagca gattacgcgc agaaaaaaag gatctcaaga agatcctttg
3901 atcttttcta cggggtctga cgctcagtgg aacgaaaact cacgttaagg gattttggtc
3961 atgagattat caaaaaggat cttcacctag atccttttaa attaaaaatg aagttttaaa
4021 tcaatctaaa gtatatatga gtaaacttgg tctgacagcg gccgcaaatg ctaaaccact
4081 gcagtggtta ccagtgcttg atcagtgagg caccgatctc agcgatctgc ctatttcgtt
4141 cgtccatagt ggcctgactc cccgtcgtgt agatcactac gattcgtgag ggcttaccat
4201 caggccccag cgcagcaatg atgccgcgag agccgcgttc accggccccc gatttgtcag
4261 caatgaacca gccagcaggg agggccgagc gaagaagtgg tcctgctact ttgtccgcct
4321 ccatccagtc tatgagctgc tgtcgtgatg ctagagtaag aagttcgcca gtgagtagtt
4381 tccgaagagt tgtggccatt gctactggca tcgtggtatc acgctcgtcg ttcggtatgg
4441 cttcgttcaa ctctggttcc cagcggtcaa gccgggtcac atgatcaccc atattatgaa
4501 gaaatgcagt cagctcctta gggcctccga tcgttgtcag aagtaagttg gccgcggtgt
4561 tgtcgctcat ggtaatggca gcactacaca attctcttac cgtcatgcca tccgtaagat
4621 gcttttccgt gaccggcgag tactcaacca agtcgttttg tgagtagtgt atacggcgac
4681 caagctgctc ttgcccggcg tctatacggg acaacaccgc gccacatagc agtactttga
4741 aagtgctcat catcgggaat cgttcttcgg ggcggaaaga ctcaaggatc ttgccgctat
4801 tgagatccag ttcgatatag cccactcttg cacccagttg atcttcagca tcttttactt
4861 tcaccagcgt ttcggggtgt gcaaaaacag gcaagcaaaa tgccgcaaag aagggaatga
4921 gtgcgacacg aaaatgttgg atgctcatac tcgtcctttt tcaatattat tgaagcattt
4981 atcagggtta ctagtacgtc tctcaaggat aagtaagtaa tattaaggta cgggaggtat
5041 tggacaggcc gcaataaaat atctttattt tcattacatc tgtgtgttgg ttttttgtgt
5101 gaatcgatag tactaacata cgctctccat caaaacaaaa cgaaacaaaa caaactagca
5161 aaataggctg tccccagtgc aagtgcaggt gccagaacat ttctct

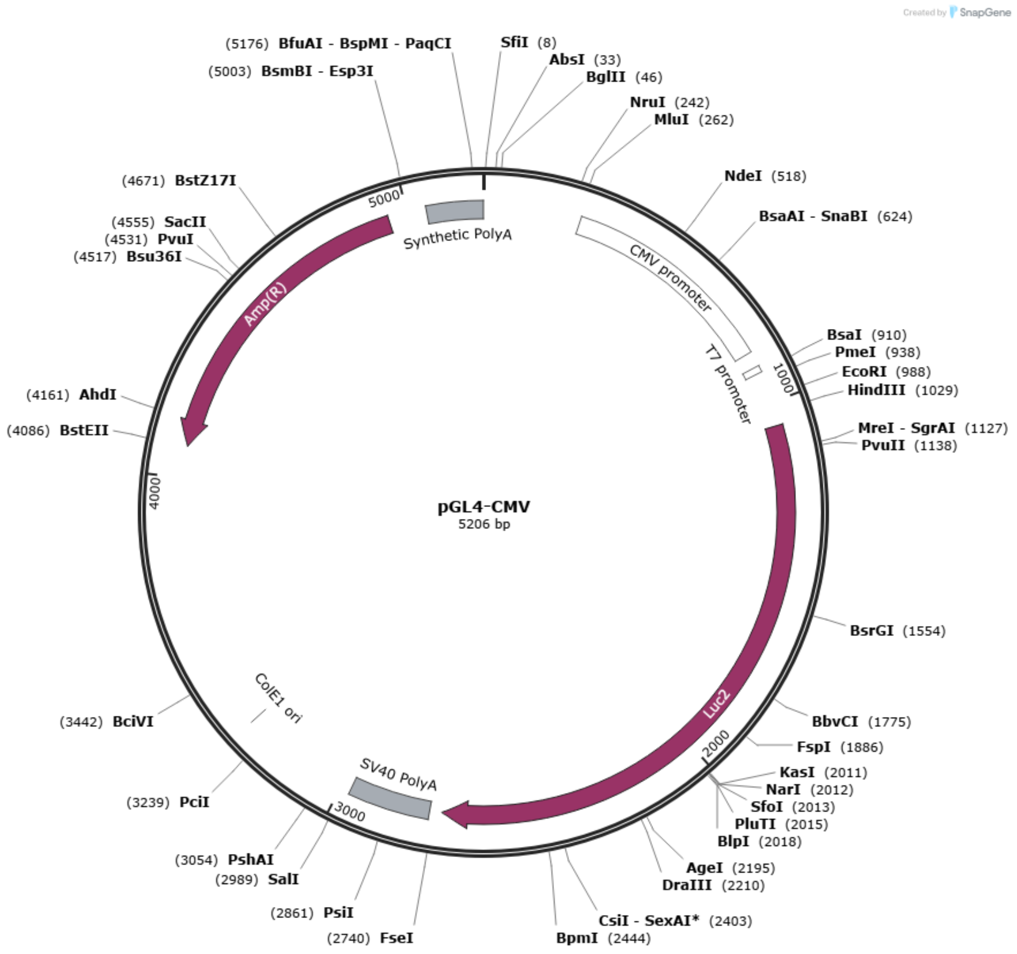
Created by SnapGene
pGL4-CMV
5206 bp
(5176) BfuAI - BspMI - PaqCI
(5003) BsmBI - Esp3I
SfiI (8)
AbsI (33)
BglII (46)
NruI (242)
MluI (262)
NdeI (518)
BsaAI - SnaBI (624)
BsaI (910)
PmeI (938)
EcoRI (988)
HindIII (1029)
MreI - SgrAI (1127)
PvuII (1138)
BsrGI (1554)
BbvCI (1775)
FspI (1886)
KasI (2011)
NarI (2012)
SfoI (2013)
PluTI (2015)
BlpI (2018)
AgeI (2195)
DraIII (2210)
CsiI - SexAI* (2403)
BpmI (2444)
(2740) FseI
(2861) PsiI
(2989) SalI
(3054) PshAI
(3239) PciI
(3442) BciVI
(4086) BstEII
(4161) AhdI
(4517) Bsu36I
(4531) PvuI
(4555) SacII
(4671) BstZ17I
Synthetic PolyA
CMV promoter
T7 promoter
Luc2
SV40 PolyA
ColE1 ori
Amp(R)
1000
2000
3000
4000
5000

7 References

1. Terrazas, M.; Eritja, R. Synthesis and Properties of Small Interfering RNA Duplexes Carrying 5-Ethyluridine Residues. *Mol. Divers*. **2011**, *15* (3), 677–686. DOI: 10.1007/s11030-010-9290-1.

2. Milisavljevič, N.; Perlíková, P.; Pohl, R.; Hocek, M. Enzymatic Synthesis of Base-Modified RNA by T7 RNA Polymerase. A Systematic Study and Comparison of 5-Substituted Pyrimidine and 7-Substituted 7-Deazapurine Nucleoside Triphosphates as Substrates. *Org. Biomol. Chem*. **2018**, *16* (32), 5800–5807. DOI: 10.1039/c8ob01498a.

3. Hadj-Bouazza, A.; Zerrouki, R.; Krausz, P.; Laumond, G.; Aubertin, A. M.; Champavier, Y. New Acyclonucleosides: Synthesis and Anti-HIV Activity. *Nucleosides Nucleotides Nucleic Acids*. **2005**, *24* (8), 1249–1263. DOI: 10.1081/NCN-200067423.

4. Ingale, S. A.; Leonard, P.; Seela, F. Glycosylation of Pyrrolo[2,3- d]Pyrimidines with 1- O-Acetyl-2,3,5-Tri- O-Benzoyl-β-d-Ribofuranose: Substituents and Protecting Groups Effecting the Synthesis of 7-Deazapurine Ribonucleosides. *J. Org. Chem*. **2018**, *83* (15), 8589–8595. DOI: 10.1021/acs.joc.8b00343.

5. Seela, F.; Peng, X. 7-Functionalized 7-Deazapurine Ribonucleosides Related to 2-Aminoadenosine, Guanosine, and Xanthosine: Glycosylation of Pyrrolo[2,3- d]Pyrimidines with 1- O -Acetyl-2,3,5-Tri- O -Benzoyl-d -Ribofuranose. *J. Org. Chem*. **2006**, *71* (1), 81–90. DOI: 10.1021/jo0516640.

6. Shanmugasundaram, M.; Senthilvelan, A.; Xiao, Z.; Kore, A. R. An Efficient Protection-Free One-Pot Chemical Synthesis of Modified Nucleoside-5′-Triphosphates. *Nucleosides Nucleotides Nucleic Acids*. **2016**, *35* (7), 356–362. DOI: 10.1080/15257770.2016.1163382.

7. Saneyoshi, M.; Watanabe, S. Synthetic Nucleosides and Nucleotides. XXVIII. Synthesis of 5-Alkylcytidines from 5-Alkylbarbituric Acids. *Chem. Pharm. Bull*. **1988**, *36* (7), 2673–2678. DOI: 10.1248/cpb.36.2673.

8. Marty, M. T.; Baldwin, A. J.; Marklund, E. G.; Hochberg, G. K. A.; Benesch, J. L. P.; Robinson, C. V. Bayesian Deconvolution of Mass and Ion Mobility Spectra: From Binary Interactions to Polydisperse Ensembles. *Anal. Chem*. **2015**, *87* (8), 4370–4376. DOI: 10.1021/acs.analchem.5b00140.

9. Kao, C.; Zheng, M.; Rüdisser, S. A Simple and Efficient Method to Reduce Nontemplated Nucleotide Addition at the 3 Terminus of RNAs Transcribed by T7 RNA Polymerase. *RNA* **1999**, *5* (9), 1268–1272. DOI: 10.1017/s1355838299991033.

10. František Potužník, J.; Nešuta, O.; Škríba, A.; Voleníková, B.; Mititelu, M.-B.; Mancini, F.; Serianni, V.; Fernandez, H.; Spustová, K.; Trylčová, J.; Vopalensky, P.; Cahová, H. Diadenosine Tetraphosphate (Ap4 A) Serves as a 5’ RNA Cap in Mammalian Cells. *Angew. Chem. Int. Ed*. **2024**, *63* (6), e202314951. DOI: 10.1002/anie.202314951.

11. Nishimasu, H.; Ran, F. A.; Hsu, P. D.; Konermann, S.; Shehata, S. I.; Dohmae, N.; Ishitani, R.; Zhang, F.; Nureki, O. Crystal Structure of Cas9 in Complex with Guide RNA and Target DNA. *Cell*. **2014**, *156* (5), 935–949. DOI: 10.1016/j.cell.2014.02.001.

12. Lee, J.; Cheng, X.; Swails, J. M.; Yeom, M. S.; Eastman, P. K.; Lemkul, J. A.; Wei, S.; Buckner, J.; Jeong, J. C.; Qi, Y.; Jo, S.; Pande, V. S.; Case, D. A.; Brooks, C. L.; MacKerell, A. D.; Klauda, J. B.; Im, W. CHARMM-GUI Input Generator for NAMD, GROMACS, AMBER, OpenMM, and CHARMM/OpenMM Simulations Using the CHARMM36 Additive Force Field. *J. Chem. Theory Comput*. **2016**, *12* (1), 405–413. DOI: 10.1021/acs.jctc.5b00935.

13. Jorgensen, W. L.; Chandrasekhar, J.; Madura, J. D.; Impey, R. W.; Klein, M. L. Comparison of Simple Potential Functions for Simulating Liquid Water. *J. Chem. Phys*. **1983**, *79* (2), 926. DOI: 10.1063/1.445869.

14. Zgarbová, M.; Otyepka, M.; Sponer, J.; Mládek, A.; Banáš, P.; Cheatham, T. E.; Jurečka, P. Refinement of the Cornell et al. Nucleic Acids Force Field Based on Reference Quantum Chemical Calculations of Glycosidic Torsion Profiles. *J. Chem. Theory Comput*. **2011**, *7* (9), 2886–2902. DOI: 10.1021/ct200162x.

15. Galindo-Murillo, R.; Robertson, J. C.; Zgarbová, M.; Šponer, J.; Otyepka, M.; Jurečka, P.; Cheatham, T. E. Assessing the Current State of Amber Force Field Modifications for DNA. *J. Chem. Theory Comput*. **2016**, *12* (8), 4114–4127. DOI: 10.1021/acs.jctc.6b00186.

16. Maier, J. A.; Martinez, C.; Kasavajhala, K.; Wickstrom, L.; Hauser, K. E.; Simmerling, C. Ff14SB: Improving the Accuracy of Protein Side Chain and Backbone Parameters from Ff99SB. *J. Chem. Theory Comput*. **2015**, *11* (8), 3696–3713. DOI: 10.1021/acs.jctc.5b00255.

17. Sousa da Silva, A. W.; Vranken, W. F. ACPYPE - AnteChamber PYthon Parser InterfacE. *BMC Res. Notes.* **2012**, *5*, 367. DOI: 10.1186/1756-0500-5-367.

18. Wang, J.; Wang, W.; Kollman, P. A.; Case, D. A. Automatic Atom Type and Bond Type Perception in Molecular Mechanical Calculations. *J. Mol. Graph. Model.* **2006**, *25*, 247–260. DOI: 10.1016/j.jmgm.2005.12.005.

19. Wang, J.; Wolf, R. M.; Caldwell, J. W.; Kollman, P. A.; Case, D. A. Development and Testing of a General Amber Force Field. *J. Comput. Chem*. **2004**, *25* (9), 1157–1174. DOI: 10.1002/jcc.20035.

20. Jakalian, A.; Jack, D. B.; Bayly, C. I. Fast, Efficient Generation of High-Quality Atomic Charges. AM1-BCC Model: II. Parameterization and Validation. *J. Comput. Chem*. **2002**, *23* (16), 1623–1641. DOI: 10.1002/jcc.10128.

21. Zahorska, E.; Kuhaudomlarp, S.; Minervini, S.; Yousaf, S.; Lepsik, M.; Kinsinger, T.; Hirsch, A. K. H.; Imberty, A.; Titz, A. A Rapid Synthesis of Low-Nanomolar Divalent LecA Inhibitors in Four Linear Steps from d-Galactose Pentaacetate. *Chem. Commun*. **2020**, *56* (62), 8822–8825. DOI: 10.1039/d0cc03490h.

22. Bussi, G.; Donadio, D.; Parrinello, M. Canonical Sampling through Velocity Rescaling. *J. Chem. Phys*. **2007**, *126* (1), 014101. DOI: 10.1063/1.2408420.

23. Bernetti, M.; Bussi, G. Pressure Control Using Stochastic Cell Rescaling. *J. Chem. Phys*. **2020**, *153* (11), 114107. DOI: 10.1063/5.0020514.

24. Essmann, U.; Perera, L.; Berkowitz, M. L.; Darden, T.; Lee, H.; Pedersen, L. G. A Smooth Particle Mesh Ewald Method. *J. Chem. Phys*. **1995**, *103* (19), 8577. DOI: 10.1063/1.470117.

25. Miyamoto, S.; Kollman, P. A. Settle: An Analytical Version of the SHAKE and RATTLE Algorithm for Rigid Water Models. *J. Comput. Chem*. **1992**, *13* (8), 952–962. DOI: 10.1002/jcc.540130805.

26. Hess, B.; Bekker, H.; Berendsen, H. J. C.; Fraaije, J. G. E. M. LINCS: A Linear Constraint Solver for Molecular Simulations. *J. Comput. Chem*. **1997**, *18* (12), 1463–1472. DOI: 10.1002/(SICI)1096-987X(199709)18:12<1463::AID-JCC4>3.0.CO;2-H.

27. Hess, B. P-LINCS: A Parallel Linear Constraint Solver for Molecular Simulation. *J. Chem. Theory Comput*. **2008**, *4* (1), 116–122. DOI: 10.1021/ct700200b.

28 Abraham, M. J.; Murtola, T.; Schulz, R.; Páll, S.; Smith, J. C.; Hess, B.; Lindahl, E. GROMACS: High Performance Molecular Simulations through Multi-Level Parallelism from Laptops to Supercomputers. *SoftwareX*. **2015**, *1–2*, 19–25. DOI: 10.1016/j.softx.2015.06.001.

29. Michaud-Agrawal, N.; Denning, E. J.; Woolf, T. B.; Beckstein, O. MDAnalysis: A Toolkit for the Analysis of Molecular Dynamics Simulations. *J. Comput. Chem*. **2011**, *32* (10), 2319–2327. DOI: 10.1002/jcc.21787.

30. Schrödinger, LLC. *The PyMOL Molecular Graphics System*, Version 1.8; Schrödinger, LLC: New York, **2015**.
